# Biocompatible piezoionic eutectogel sensor for self-powered physiological monitoring

**DOI:** 10.1038/s41467-026-77390-7

**Published:** 2026-09-23

**Authors:** Bo Wu, Xuejie Liu, Zhiheng Zeng, Bolong Li, Haoyu Fang, Derek Ho

**Affiliations:** 1 Department of Materials Science and Engineering, City University of Hong Kong, Kowloon, Hong Kong, China; 2 Hong Kong Centre for Cerebro-cardiovascular Health Engineering, Hong Kong Science Park, Hong Kong, China; 3 Institute of Digital Medicine, City University of Hong Kong, Kowloon, Hong Kong, China

**Keywords:** Composites, Gels and hydrogels, Sensors and biosensors

## Abstract

Piezoionics holds promise for self-powered bioelectronics, yet existing hydrogel and elastomer systems are often limited by poor biocompatibility, environmental instability, and low output. Here, we report a biocompatible deep eutectic solvent-polyvinyl alcohol (DES-PVA) eutectogel featuring a robust piezoionic response and practical wearable sensing capabilities. By incorporating a choline chloride-glycerol DES into a PVA network, we engineer tailored hydrogen bonds that selectively constrain choline cations while enabling rapid chloride anion transport, thereby amplifying the piezoionic response to achieve a sensitivity of 60 mV kPa^–1^. In vitro assays verify the excellent biocompatibility of the eutectogel. Furthermore, practical application demonstrations underscore its capability for precise and continuous monitoring of diverse physiological signals, including pulse waveform and electrocardiograms (ECG), with fidelity comparable to commercial sensors. Overall, this work establishes a self-powered, bio-compliant epidermal platform for advanced iontronics and bioelectronics.

## Abstract (web summary)

Piezoionics enables effective mechanical-to-electrical conversion in self-powered bioelectronics. Here, the authors develop a biocompatible eutectogel with tailored hydrogen-bonded networks, achieving excellent piezoionic properties for high-fidelity physiological signal monitoring.

## Introduction

The rapid advancement of self-powered technologies is reshaping the landscape of wearable electronics, biomedical diagnostics, and sustainable energy systems, accelerating the shift from externally powered devices to autonomous and adaptive platforms^1–4^. Among various strategies, mechanical-to-electrical energy conversion has attracted widespread attention, as ubiquitous mechanical motions and subtle physiological activities can be directly transformed into electrical signals^5–8^. Unlike conventional sensing modalities that merely modulate signals supplied by external power sources, stimulus-responsive energy generation enables both active sensing and energy autonomy, thereby offering a fundamentally different operational paradigm^9^. Within this context, triboelectric and piezoelectric mechanisms have been extensively explored^10–12^. Triboelectric nanogenerators (TENGs) generate electrical outputs through contact-induced electron transfer between dissimilar materials, offering low-cost fabrication, high voltage output, and favorable performance for high-frequency mechanical excitation^10,11^. However, their limited sensitivity to low-pressure stimuli and susceptibility to environmental variations constrain their utility in precision sensing applications. In contrast, piezoelectric materials convert mechanical stress into electrical charges through polarization, enabling rapid response and high signal stability across a broad range of mechanical amplitudes and frequencies^5,12^. Nevertheless, the high Young’s modulus of most piezoelectric materials constitutes a fundamental mechanical mismatch with soft skin, which limits the realization of conformable and reliable interfaces for bio-integrated applications^13,14^.

**Fig. 1:**
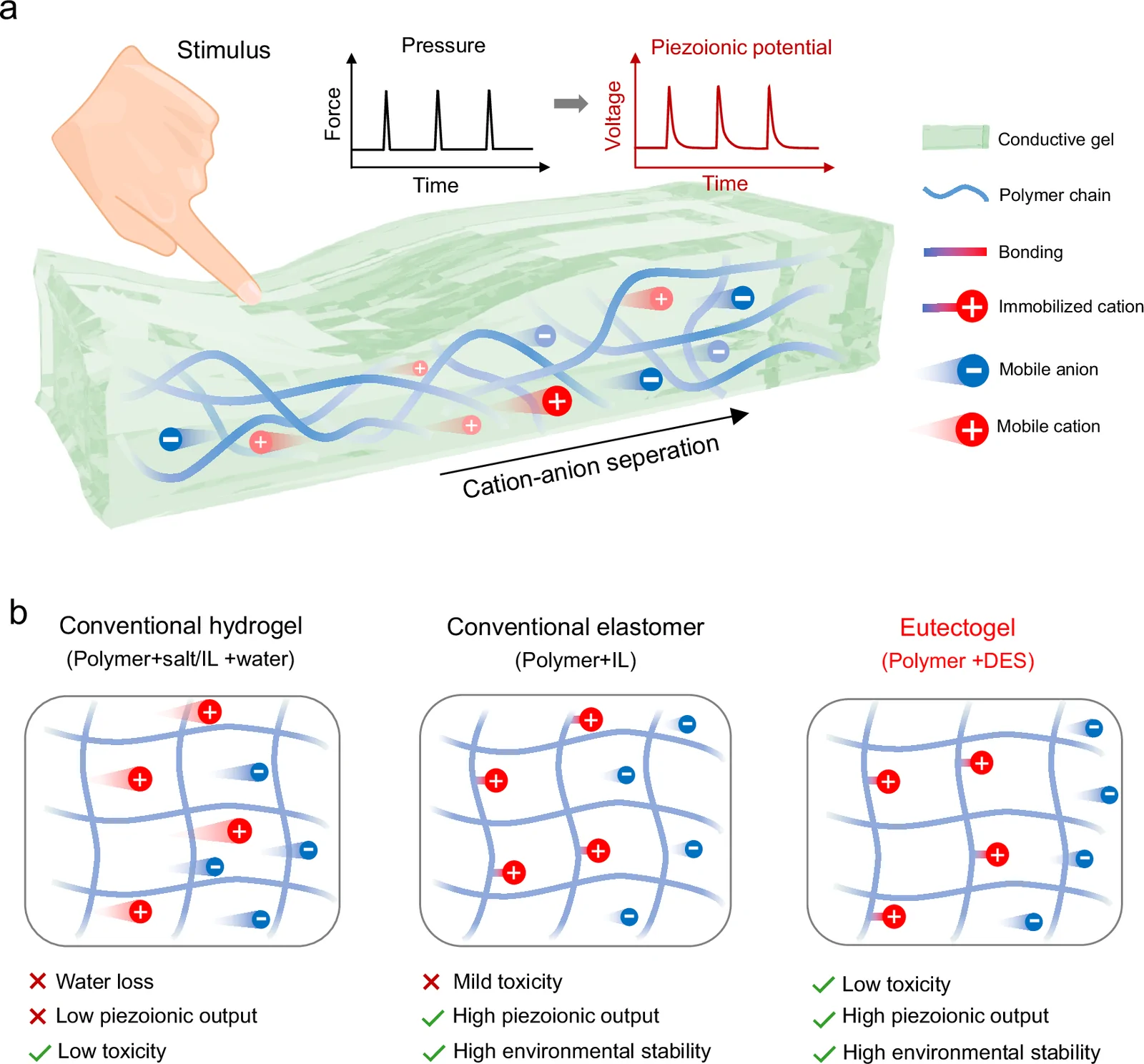
Electromechanical transduction of piezoionic materials. **a** Schematic illustration of the piezoionic mechanism of ionic conductors. **b** Comparison of anion and cation transport mechanisms in hydrogel, elastomer, and eutectogel.

The piezoionic effect introduces an effective means of mechanoelectrical transduction that addresses the aforementioned limitations. Piezoionics converts mechanical stimuli into electrical signals through pressure-induced ionic redistribution within ionically conductive polymer networks^15^, as illustrated in Fig. 1a. Under mechanical deformation, cations and anions are separated. Due to the difference in ion transport, an ionic current is generated^7^. When employed as biosensors, piezoionic devices exhibit a distinct set of advantages. First, piezoionic signal generation typically delivers a high current density relative to its voltage output, which closely matches the signaling characteristics of neural and electrophysiological activities in biology^16^. Second, the piezoionic effect is a generation mechanism. Thus, in the context of sensing, it provides a self-powered, energy-autonomous solution that removes the requirement of an external power source^17^. Third, the realization of piezoionics in soft materials offers intrinsic mechanical compatibility with biology^16,18^. Not only are soft materials comfortable to wear long-term, they also mitigate the serious signal integrity issues due to motion^16,19^.

Existing piezoionic materials can be broadly classified into two categories according to the polymer matrix: hydrogels and elastomers, as illustrated in Fig. 1b. Ionic hydrogels consist of a polymer network swollen with aqueous salt solutions, which are highly biocompatible. Thus, piezoionic hydrogels have been widely applied to biosensing, as exemplified by our previous crown ether-grafted PVA hydrogel used as a piezoionic skin for pressure sensing^20^. Despite these merits, the practical deployment of hydrogels is intrinsically limited by water evaporation and limited mechanical performance. In contrast, elastomers incorporating ionic liquids (ILs) as ion-conductive phases offer an alternative by eliminating water-related instability^17,21,22^. The anhydrous nature of ILs endows ionic elastomers with enhanced environmental robustness, while the wide compositional diversity of ILs enables high piezoionic responses through precisely tunable ionic interaction and generally superior mechanical performance, such as strength and toughness^17^. Nevertheless, many commonly employed ILs exhibit cytotoxicity, which has been shown to irritate human skin, which severely limits their safe adoption as wearable or epidermal bio-integrated devices^23–25^.

Deep eutectic solvents (DESs), formed through dynamic hydrogen-bonding interactions between hydrogen-bond donors and acceptors, have recently emerged as a promising, truly biocompatible alternative to ILs^26^. DESs retain many of the favorable physicochemical properties of ILs while offering superior biosafety, high environmental stability, and tunable solvation capability. Owing to these hydrogen-bonding interaction, DESs can effectively regulate polymer network interactions, chain conformations, and microstructure, thereby enabling the realization of eutectogels that combine high ionic conductivity with biocompatibility^27,28^. Beyond these general advantages, the presence of abundant and a tunable amount of hydrogen-bond acceptors within DES components allows selective interactions with ionic species, leading to the preferential immobilization of ions of one polarity while allowing enhanced transport of the counterions^27,29,30^. Such selective ionic interaction dynamics provide an effective means for amplifying piezoionic outputs. However, despite these appealing attributes, prior investigations of eutectogels have primarily focused on resistive strain sensing, while their potential as biocompatible and self-powered piezoionic sensors remains largely unexplored.

Herein, we present a soft piezoionic eutectogel biosensor with excellent biocompatibility for physiological signal monitoring. The sensor is fabricated by incorporating a choline chloride (ChCl)-glycerol deep eutectic solvent (DES) into a polyvinyl alcohol (PVA) polymer matrix. Through engineering the hydrogen-bond network, selective ion transport enhances piezoionic transduction. This is a demonstration of a piezoionic eutectogel biosensor utilized in physiological signal monitoring with excellent biocompatibility, stability and signal fidelity. Experimental and computational investigations both reveal that mechanical stimulation induces a deliberate asymmetry in cation and anion mobility, where choline cations (Ch⁺) are selectively immobilized through hydrogen-bond interactions with PVA chains, while chloride anions (Cl⁻) retain high mobility. This mechanism enables a pronounced piezoionic voltage of up to 72 mV and a current output of 3.5 μA under a low pressure of 15 kPa, highlighting clear advantages in sensitivity and power generation. In parallel, in vitro cell viability assays confirm the favorable biocompatibility of the eutectogel. Building on the robust piezoionic transduction, the biocompatible eutectogel sensor enables stable and repeatable monitoring of physiological pressure signals, including arterial pulse waveforms and vocalization-induced vibrations. Moreover, its high intrinsic ionic conductivity supports faithful bioelectrical signal acquisition, achieving electrocardiographic (ECG) recordings with performance comparable to that of commercial hydrogel electrodes. This piezoionic eutectogel sensor integrates biocompatibility, mechanical adaptability, and self-powered signal transduction, providing a potential route toward soft iontronics and bioelectronics for physiological monitoring.

## Results

### Molecular and structural design of DES-PVA eutectogels

**Fig. 2:**
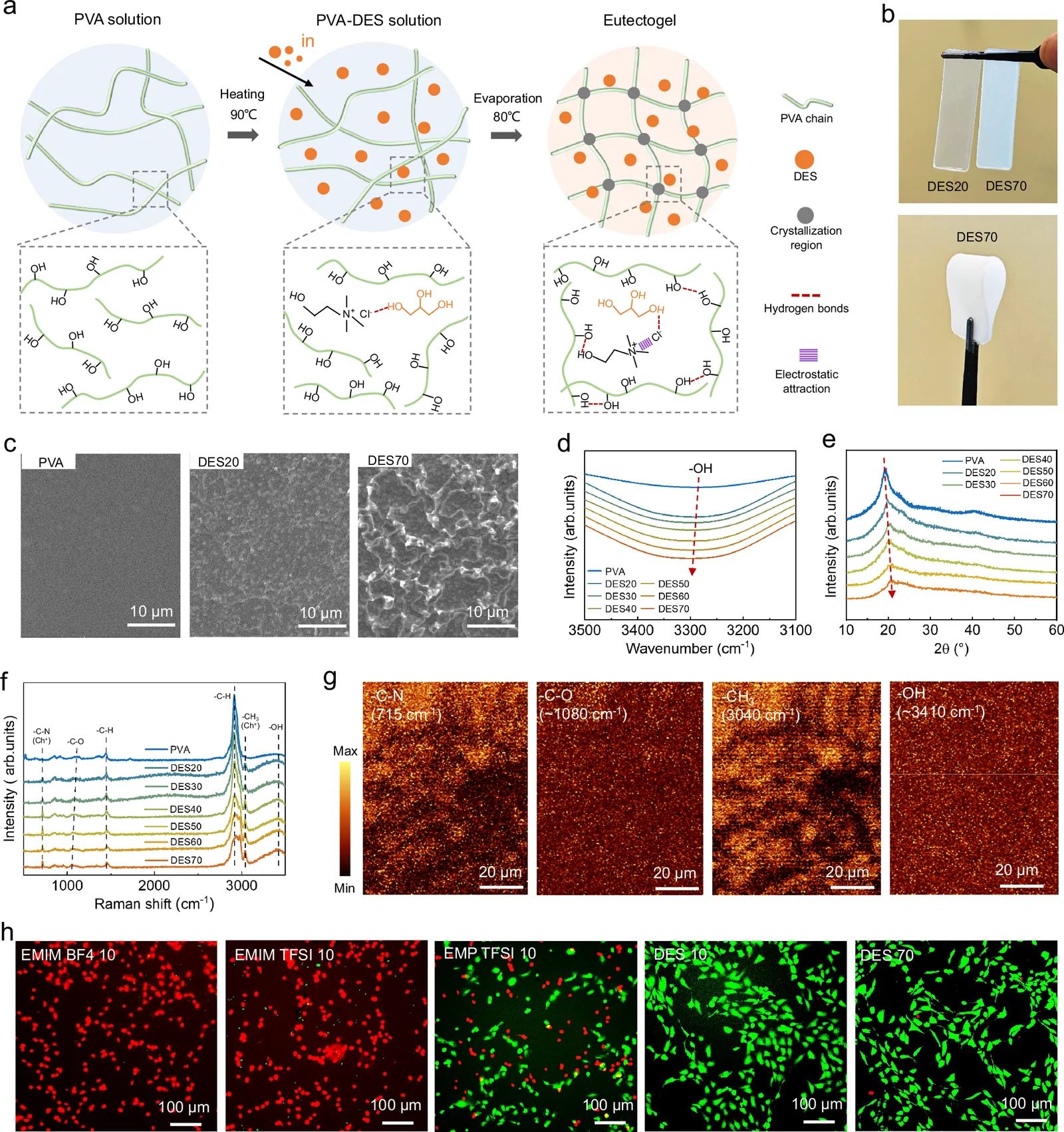
Structural and characterization of DES-PVA eutectogels. **a** Schematic illustration of the preparation of eutectogels. **b** Photographs of the DES20 and DES70 eutectogels, where DES20 and DES70 denote eutectogels containing 20 wt% and 70 wt% DES, respectively. **c** Scanning electron microscopy (SEM) images of PVA film, DES20, and DES70. *n* = 3 independent samples. **d** Fourier transform infrared (FTIR) spectra of eutectogels with different DES contents. **e** X-ray diffraction (XRD) patterns of the eutectogels with different DES contents. **f** Raman spectra of eutectogels with different DES contents. **g** Raman mapping images corresponding to characteristic functional groups in the eutectogels. *n* = 3 independent samples. **h** Cell viability comparison between PVA-IL gels and DES-PVA eutectogels. *n* = 3 independent samples.

To combine high piezoionic efficiency with interfacial biocompatibility, we developed a eutectogel platform by incorporating a ChCl-glycerol DES into a PVA matrix. This design exploits tailored hydrogen-bonding interactions between the DES and PVA chains to selectively immobilize Ch⁺ cations while enabling rapid Cl⁻ anion transport under mechanical deformation, thereby amplifying the piezoionic output through enhanced charge asymmetry. The design rationale and resultant network topology of the DES-PVA eutectogels are illustrated in Fig. 2a and Supplementary Fig. 1. The eutectogels were fabricated through a two-step process, involving the formation of a homogeneous DES-PVA solution via dropwise addition of DES into heated PVA, followed by thermal treatment to induce gelation (Supplementary Fig. 2 and Supplementary Note 1). In addition, the miscibility between PVA and glycerol remains limited, restricting the maximum DES content that can be homogeneously incorporated (Supplementary Fig. 3). Eutectogels obtained after thermal treatment likewise exhibit phase separation, which is manifested as a translucent white appearance when the DES content exceeds 20 wt% (Fig. 2b). Specifically, DES20 and DES70 denote the eutectogels containing with 20 wt% and 70 wt% DES, respectively. This macroscopic transition suggests the development of microphase-separated domains within the PVA network^31–33^.

Multiple characterization, including scanning electron microscopy (SEM), Fourier transform infrared (FTIR) spectra, X-ray diffraction (XRD) patterns, consistently support this structural evolution. SEM images reveal distinct microphase-separated domains in DES70 compared to pristine PVA film and DES20 samples (Fig. 2c). FTIR spectroscopy shows that the O-H stretching band around 3280 cm^–1^ progressively shifts toward higher wavenumbers with increasing DES content, indicating a weakening of average hydrogen-bond strength and a reduced contribution from strongly hydrogen-bonded hydroxyl groups (Fig. 2d and Supplementary Fig. 4). XRD analysis further reveals a progressive reduction in overall crystallinity with increasing DES content (Fig. 2e). Meanwhile, the characteristic PVA diffraction peak exhibits a blue shift, indicating contraction of interchain spacing within the remaining ordered PVA domains. Further, Raman spectroscopy was employed to directly visualize the spatial distribution of specific ionic components associated with this structural heterogeneity. In the Raman spectra, Ch^+^-related vibrational bands at approximately 715 cm^–1^ (C-N stretching in Ch⁺) and 3040 cm^–1^ (N-CH₃ stretching in Ch⁺) evolve systematically with increasing DES content, reflecting variations in Ch⁺ concentration (Fig. 2f and Supplementary Fig. 5). Raman mapping based on these Ch^+^-specific bands reveals pronounced spatial intensity heterogeneity, visualizing the non-uniform distribution of Ch^+^ ions and providing direct evidence for DES phase separation (Fig. 2g, Supplementary Note 2, and Supplementary Fig. 6).

To assess cytocompatibility, in vitro cell viability assays were conducted on conventional PVA-IL gels and the DES-PVA eutectogels (Fig. 2h). PVA-IL gels incorporating commonly used ILs, including EMIMBF₄, EMIMTFSI, and EMPTFSI, induced pronounced cellular damage, characterized by poor adhesion and markedly reduced viability. In contrast, DES-PVA eutectogels supported dense and uniform cell adhesion, maintaining good cell viability at both 10 wt% and 70 wt% DES loadings. The well-spread cell morphology observed even at high DES contents highlights the superior cytocompatibility of the proposed eutectogels, underscoring their suitability as epidermal bioelectronics and biointerfaces.

### Mechanical and piezoionic performance of eutectogels

**Fig. 3:**
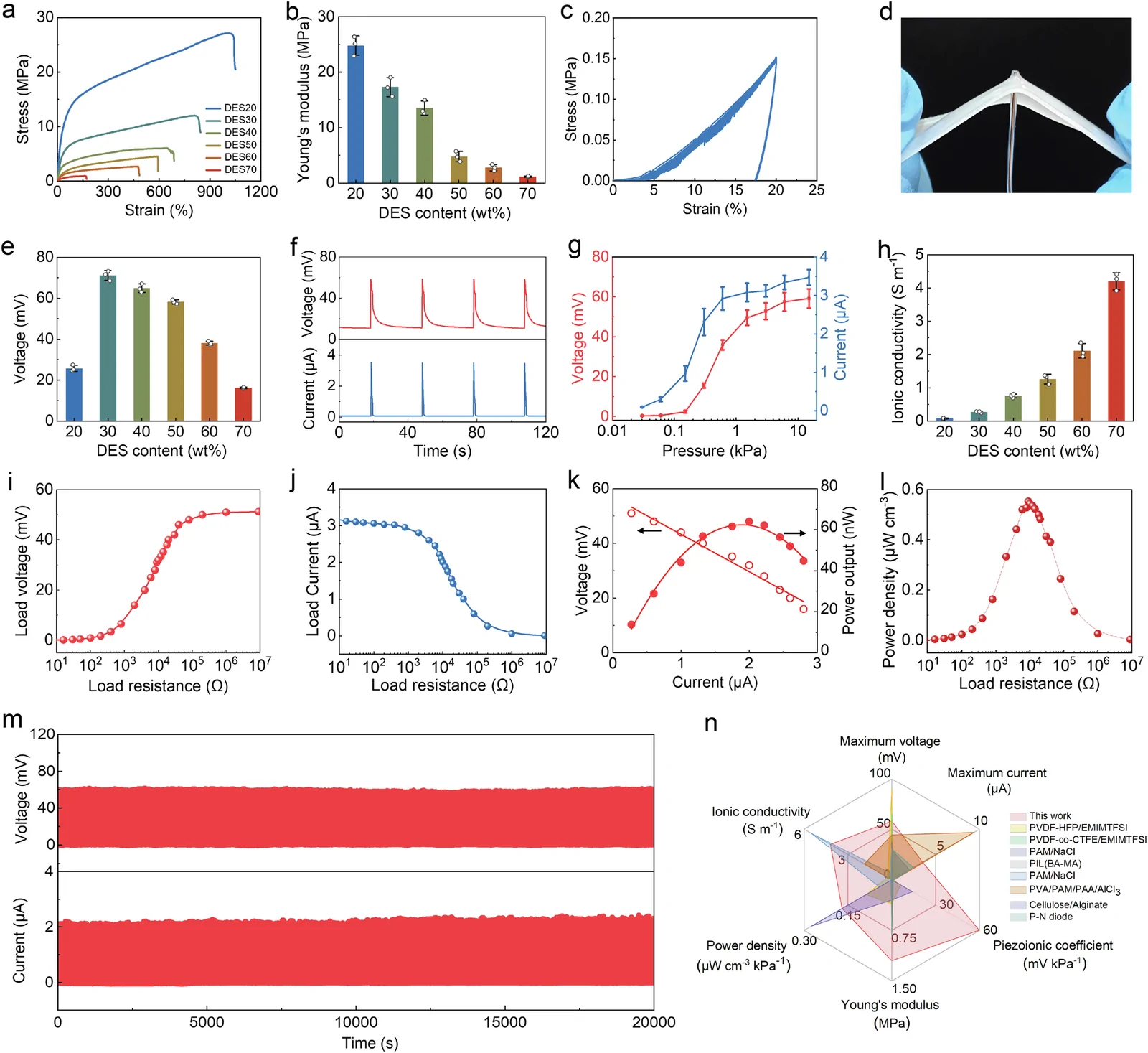
Mechanical and piezoionic performance of DES-PVA eutectogels. **a** Stress-strain curves of eutectogels across DES contents. **b** Young’s modulus of eutectogels as a function of DES content. *n* = 3 independent samples. **c** Compression cyclic testing of the eutectogel under 20% compressive strain. **d** Puncture resistance of eutectogel. **e** Piezoionic voltage of eutectogels with different DES contents under an applied pressure of 15 kPa. *n* = 3 independent samples. **f** Voltage and current responses at a pressure of 15 kPa. **g** Piezoionic voltage and current within a pressure range of 15 kPa. *n* = 3 independent samples. **h** Ionic conductivity of eutectogels with different DES content. *n* = 3 independent samples. **i** Load voltage and (**j**) current as a function of load resistance under 3 kPa. (**k**) Voltage and corresponding power output as a function of current under 3 kPa. **l** Power density with different load resistances under 3 kPa. **m** Repeated voltage and current output cycle under a pressure of 3 kPa, obtained from independent voltage and current measurements. **n** Performance comparison of our eutectogels with recently reported piezoionic materials, highlighting superior power density, output voltage, output current, ionic conductivity, Young’s modulus, and piezoionic coefficient. All statistical analyses were performed using one-way ANOVA, and Data are presented as mean value ± standard deviation (SD).

The mechanical properties of the eutectogel are investigated. As shown in Fig. 3a, the stress-strain curves of the eutectogels exhibit pronounced dependence on DES content, reflecting the progressive restructuring of the polymer network. As a result, the Young’s modulus decreases markedly with increasing DES content (Fig. 3b). The mechanical robustness of the eutectogels was further evaluated through cyclic tensile loading-unloading tests (Supplementary Fig. 7). Cyclic compression tests conducted under stresses below 0.15 MPa show highly stable responses with minimal hysteresis (Fig. 3c), confirming that the eutectogel maintains mechanical integrity under compressive loading. In addition, puncture resistance tests demonstrate that the eutectogels can withstand localized mechanical stress without structural failure (Fig. 3d). These results establish that the eutectogels exhibit excellent mechanical properties for piezoionic operation.

The effect of DES content on piezoionic performance was then examined under a constant applied pressure of 15 kPa (Fig. 3e and Supplementary Figs. 8–10). The piezoionic voltage strongly depends on DES content, reaching a maximum of 72 mV at 30 wt% DES. However, a further increase in DES content introduces an excessive amount of liquid so as to cause phase separation and weakened mechanical properties, thereby resulting in piezoionic voltage degradation. Considering the simultaneous requirements for mechanical robustness and efficient piezoionic transduction, DES50, defined as the eutectogel containing 50 wt% DES, was selected for piezoionic output evaluation. Figure 3f shows the piezoionic voltage and piezoionic current of the DES50 eutectogel under 15 kPa, yielding the values of approximately 60 mV and 3.5 μA, respectively. To comprehensively evaluate the pressure-dependent piezoionic response, the eutectogel was tested over a wide pressure range from 30 Pa–15 kPa, as shown in Fig. 3g and Supplementary Figs. 10, 11. In the pressure range of 0.1–1 kPa, both the piezoionic voltage and current increase rapidly with increasing pressure, indicating high sensitivity to subtle mechanical stimuli. When the applied pressure exceeds 1 kPa, the output gradually approaches saturation. The high current output can be attributed to the high ionic conductivity of the eutectogels (Fig. 3h and Supplementary Fig. 12). According to the formula $\nabla V=\alpha \nabla P$, where *α* denotes the piezoionic coefficient and ∇*P* represents the pressure gradient, the extracted piezoionic coefficient reaches a maximum at ~ 0.6 kPa, attaining a maximum value of 60 mV kPa^–1^ (Supplementary Fig. 13). This coefficient exceeds those reported for representative piezoionic hydrogels (~ 2 mV kPa^–1^) and ionic elastomers (6.0 mV kPa^–1^) by nearly an order of magnitude, highlighting the exceptional pressure sensitivity enabled by the DES-PVA eutectogels^17,20,21,34^.

To evaluate power output, we further tested the piezoionic device under varying external load resistances. As shown in Fig. 3i, j, the output voltage increases monotonically with respect to load resistance while the current gradually decreases, which is the expected behavior from classic circuit theory. Figure 3k depicts the piezoionic voltage and output power as a function of loading current under 3 kPa, where the voltage-current curve varies linearly with a maximum output power of 64 nW at 32 mV and 2 μA. The calculated power density reaches a maximum when the external load resistance closely matches the internal resistance of approximately 9 kΩ, as shown in Fig. 3l. Under this optimal condition and an applied pressure of 3 kPa, the eutectogel achieves a maximum power density of approximately 0.5 μW cm^–3^. Further, the piezoionic eutectogel exhibits excellent long-term stability and durability (Fig. 3m), maintaining highly consistent electrical outputs after storage for over 6 months (Supplementary Figs. 14, 15). Finally, key performance metrics of the DES-PVA eutectogel, including output voltage, current, piezoionic coefficient, power density, and Young’s modulus, were compared with those of recently reported piezoionic materials^7,17,34–39^. As summarized in Fig. 3n, the proposed eutectogel exhibits a balanced and high-performance profile, achieving a piezoionic voltage of approximately 60 mV, a current of 3.5 μA, a piezoionic coefficient of 60 mV kPa^–1^, a Young’s modulus of 1.2 MPa, an ionic conductivity of 4.2 S m^–1,^ and a normalized power density of 0.17 μW cm^–3^ kPa^–1^.

### Piezoionic diffusion kinetics

**Fig. 4:**
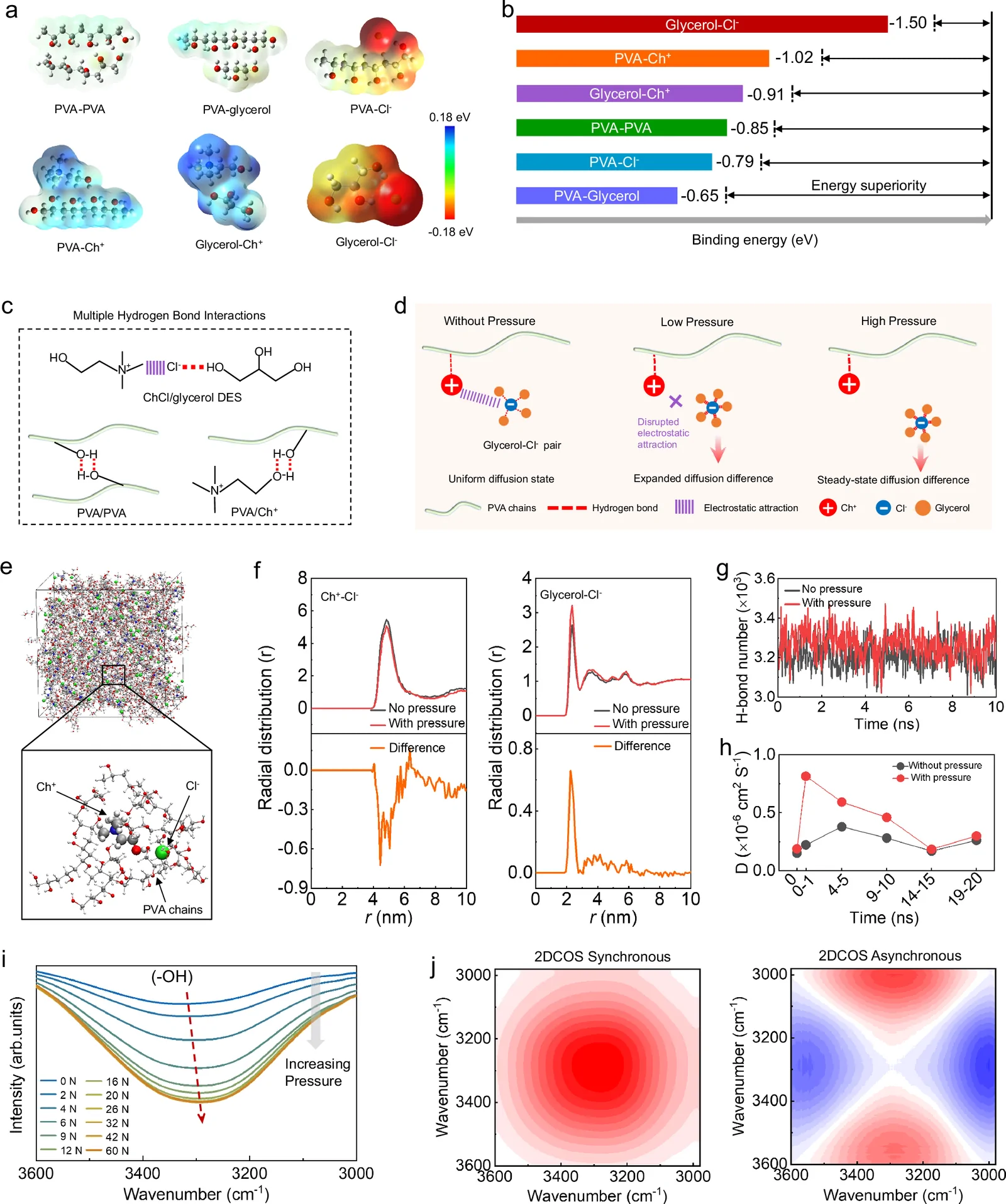
Piezoionic mechanism of DES-PVA eutectogels. **a** Electrostatic surface potential (ESP) of molecular pairs within the eutectogel material system, including PVA-PVA, PVA-glycerol, PVA-Cl⁻, PVA-Ch^+^, glycerol-Ch^+^, and glycerol-Cl⁻. **b** Calculated binding energies of the component pairs derived from density functional theory (DFT), comparing their interaction strengths. **c** Dominant hydrogen-bonding and electrostatic interactions within the eutectogel network. **d** Piezoionic mechanism for this DES-PVA eutectogel, illustrating pressure-induced redistribution of hydrogen bonding, electrostatic interactions, and the resulting asymmetry in anion-cation mobility. **e** Atomistic models of the DES-PVA eutectogel for molecular dynamics (MD) simulations, with a partially enlarged view of the solvation structure. Mechanical compression is applied to the simulation box to enable comparison between unloaded and pressurized states, as shown in Supplementary Fig. 16. **f** Radial distribution functions (RDFs) of Ch^+^-Cl⁻ and glycerol-Cl⁻ pairs under unloaded and pressurized conditions. **g** Temporal evolution of the total number of hydrogen bonds within the eutectogel network under applied pressure. **h** Time-dependent diffusivity difference between Ch⁺ and Cl⁻ ions under applied pressure extracted from MD simulations under mechanical loading. **i** Pressure-depended FTIR spectra acquired under applied forces ranging from 0 to 60 N. **j** 2D correlation spectroscopy (2DCOS) synchronous and asynchronous maps derived from pressure-dependent FTIR data.

To further elucidate the origin of the piezoionic mechanism at the molecular level, the bonding interactions within the DES-PVA eutectogel were systematically investigated. As illustrated in Fig. 4a, b, density functional theory (DFT) calculations show that the glycerol-Cl⁻ pair exhibits substantially higher binding energy than all other molecular pairs within the eutectogel network (Supplementary Note 3). This result indicates that Cl⁻ anions preferentially coordinate with glycerol molecules through strong hydrogen bonding, rather than interacting directly with PVA chains or Ch⁺ cations^40,41^. Meanwhile, PVA chains form an interconnected polymer network via intermolecular hydrogen bonding and simultaneously establish secondary hydrogen-bonding interactions with Ch⁺ cations. The dominant hydrogen-bonding configurations inferred from these DFT results are schematically summarized in Fig. 4c.

Based on these interactions, the piezoionic conduction process was evaluated under three pressure regimes: no pressure, low pressure, and high pressure, as conceptually illustrated in Fig. 4d. We investigated the pressure-dependent ion-polymer interactions underlying the piezoionic behavior through molecular dynamics (MD) simulations (Supplementary Note 4). Atomistic models of the DES-PVA eutectogel were constructed without predefined chemical bonds, allowing hydrogen bonding and electrostatic interactions to emerge spontaneously during equilibration (Fig. 4e). Mechanical pressure was subsequently applied to the simulation box, enabling a comparison between the unloaded and compressed states (Supplementary Fig. 16). Radial distribution functions (RDFs) of Ch^+^-Cl⁻, glycerol-Cl⁻ and PVA-Ch^+^ pairs were analyzed to elucidate pressure-induced changes in local coordination (Fig. 4f and Supplementary Fig. 17a). For the Ch⁺-Cl⁻ pair, the first g(*r*) peak appears at approximately 4.8 Å, characteristic of a solvent-separated ion pair stabilized predominantly by long-range electrostatic interactions, in which the cation and anion remain spatially correlated but are separated by intervening solvent molecules (Fig. 4f). In the uncompressed state, the relatively high peak intensity indicates a substantial probability of such electrostatically correlated Ch^+^-Cl⁻ associations. Upon the application of pressure, the pronounced reduction in peak intensity signifies a decreased likelihood of Ch⁺-Cl⁻ pairing, reflecting a pressure-induced destabilization of this electrostatic correlation. In contrast, the glycerol-Cl⁻ RDF exhibits a sharp first coordination peak centered at 2.0–2.2 Å, corresponding to strong short-range hydrogen bonding between Cl⁻ and glycerol hydroxyl groups. Notably, this peak becomes significantly more intense upon compression, reflecting an increased coordination of glycerol around Cl⁻. This behavior indicates the formation of a continuous glycerol-mediated transport pathway that facilitates rapid Cl⁻ migration under pressure. Meanwhile, the PVA-Ch⁺ RDF calculated between the hydroxyl oxygen atoms of PVA and the quaternary ammonium nitrogen of Ch^+^ shows a first coordination peak at approximately 4.4 Å, reflecting spatially extended ion-polymer interactions. As shown in Supplementary Fig. 17a, the increased peak intensity under compression indicates an elevated probability of Ch^+^ being transiently coordinated to the PVA network, accompanying the pressure-induced weakening of Ch⁺-Cl⁻ electrostatic pairing. At the higher pressure, the RDF peaks corresponding to Ch⁺-Cl⁻, Cl⁻-glycerol, and Ch⁺-PVA interactions remain essentially unchanged (Supplementary Figs. 17b–d), indicating that the local chemical structure becomes insensitive to further pressure increases. This behavior is consistent with the experimental observations (Fig. 3g). The increase in hydrogen bond population is in agreement with the strengthened glycerol-Cl⁻ coordination revealed by the RDF profiles (Fig. 4g). Collectively, these RDF evolutions reveal a pressure-driven redistribution of ionic coordination preferences rather than a uniform modulation of interaction strength.

To further elucidate the effect of mechanical pressure on ionic transport, mean squared displacement (MSD) analysis was employed to evaluate the diffusivities of Ch⁺ and Cl⁻ ions using Einstein relation^20^: ${D}_{t}=\frac{1}{6}{{\mathrm{lim}}}_{t\to {\infty }}\frac{d}{{dt}}\langle | {r}_{t}^{c}\left({{\rm{t}}}\right)-{r}_{t}^{c}\left(0\right){| }^{2}{{\rangle }}$, where ${r}_{t}^{c}\left({{\rm{t}}}\right)$ denotes the center-of-mass position of an ion at time *t*. When the material is in the uncompressed state (*t* = 0 ns), both cations and anions undergo thermally driven Brownian motion with isotropic diffusion and zero net directional velocity, resulting in comparable diffusivities of Ch⁺ and Cl⁻ (Fig. 4h). Upon compression, a clear divergence between anionic and cationic transport behavior emerges. Specifically, the diffusivity of Cl⁻ increases more markedly than that of Ch⁺ at moderate pressures (Supplementary Figs. 18, 19). To quantify the ionic gradient, the diffusivity difference between cations and anions, ${D}_{d}={D}_{{{Cl}}^{-}}-{D}_{{{Ch}}^{+}}$, is computed. In the absence of pressure, Cl⁻ and Ch⁺ exhibit nearly identical diffusivities over the entire simulation time, indicating symmetric ionic transport behavior without selectivity. In contrast, upon compression, a pronounced ionic mobility asymmetry emerges immediately. During the initial response window (*t* = 0–1 ns), the maximum ionic diffusivity of Cl⁻ and Ch⁺ in the compressed system reach 1.92 × 10^–6^ and 1.10 × 10^–6 ^cm^2 ^s^–1^, respectively, yielding a maximum diffusivity difference Δ*D* of 0.82 cm^2^ s^–1^. This result demonstrates that mechanical loading instantaneously breaks the symmetry of ionic transport. Together, the RDF and MSD analyses reveal that external pressure regulates the balance between ion-dipole coordination and hydrogen bonding within the eutectogel network. Strengthened glycerol-Cl⁻ coupling promotes rapid Cl⁻ migration within the glycerol phase, whereas the PVA-Ch⁺ interaction more effectively immobilizes the Ch^+^ within the PVA framework. The combined effects of ionic size disparity and selective hydrogen-bond immobilization form the dynamic basis for the pressure-driven piezoionic behavior of the DES-PVA eutectogels.

To elucidate the evolution of hydrogen bonding interactions under compression, pressure-dependent Fourier-transform infrared (FTIR) spectra were collected over a loading range of 0–60 N (Fig. 4i and Supplementary Fig. 20). In the O-H stretching region (3000–3600 cm^–1^), progressive compression induces a systematic redshift accompanied by a pronounced increase in peak intensity within the band, indicating the strengthening of hydrogen-bond interactions involving hydroxyl groups. Due to the broad O-H stretching envelope comprises multiple overlapping hydroxyl sub-bands, two-dimensional correlation spectroscopy (2DCOS) was further employed to resolve the pressure-dependent responses of distinct hydrogen-bonding species (Fig. 4j and Supplementary Note 5). Based on Noda’s judging rule^42–44^, the pressure-responsive sequence follows 3278 cm^–1^ → 3291 cm^–1^ → 3544 cm^–1^ → 2998 cm^–1^, where the arrow denotes an earlier response, corresponding respectively to *ν*(-OH, glycerol-Cl⁻), *ν*(-OH, PVA-Ch^+^), *ν*(-OH, free), *ν*(-CH_2_, stretching), respectively^45–49^. This sequence indicates that hydrogen-bond configurations associated with glycerol-Cl⁻ interactions are more responsive to mechanical compression than those involving PVA-Ch⁺ coordination, revealing a differentiated pressure response among ionic environments. Notably, no pronounced 2DCOS cross-peaks are observed in the 2800–3000 cm^–1^ region (Supplementary Fig. 21), confirming that the C-H stretching modes of the backbone do not undergo significant pressure-correlated changes. Together, the pressure-dependent FTIR and 2DCOS analyses demonstrate that mechanical compression selectively perturbs the hydrogen-bond network while leaving the covalent backbone largely intact. This spectroscopic distinction is fully consistent with the ion-specific coordination and transport behavior inferred from MD simulations and the observed piezoionic response of the DES-PVA eutectogels.

### Applications of piezoionic epidermal sensors

**Fig. 5:**
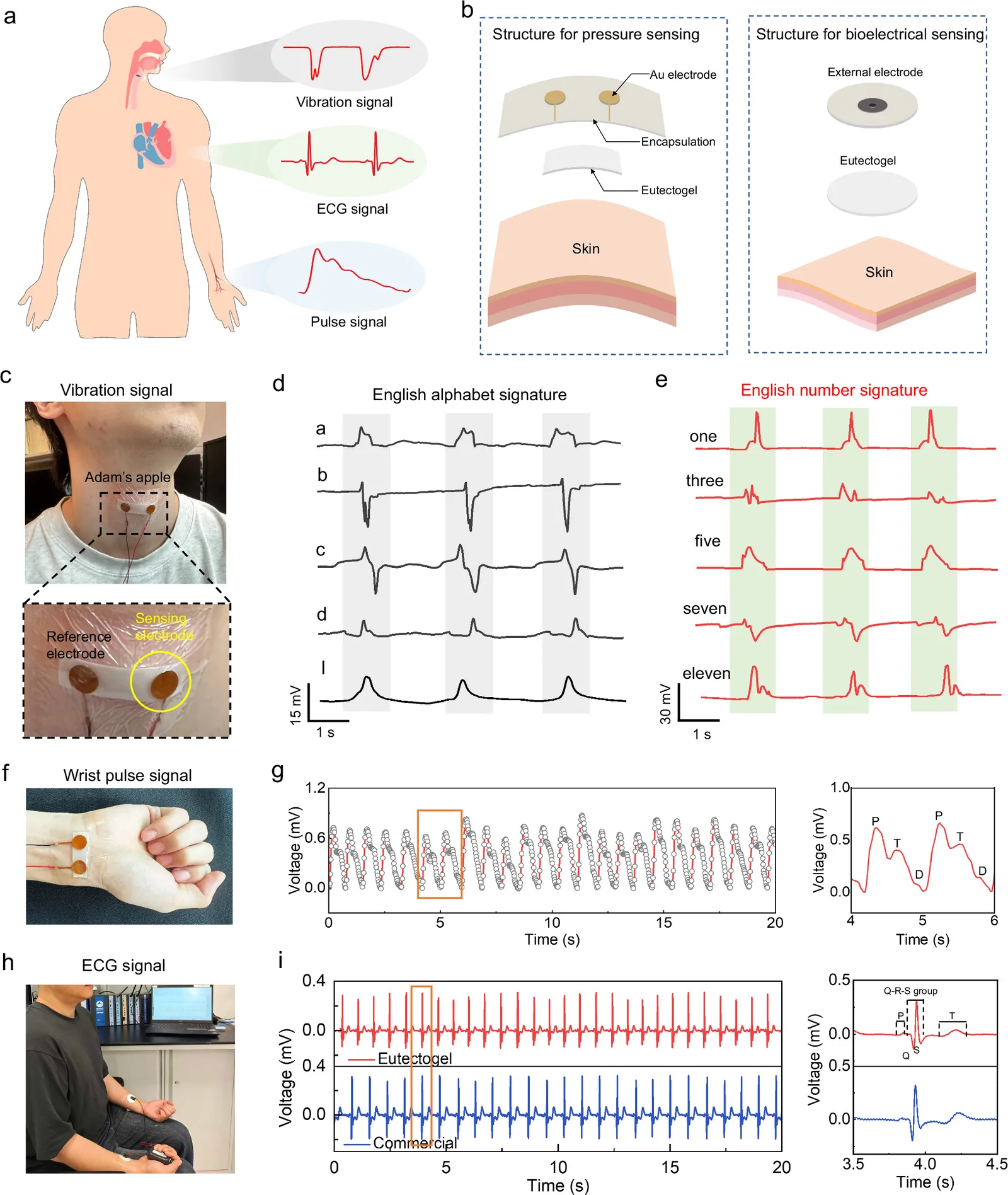
Practical application of piezoionic eutectogel sensors as epidermal human physiological signal diagnostic devices. **a** Mechanical and bioelectric signals from the human body. **b** Two piezoionic device architectures for mechanical and bioelectric signal acquisition respectively. **c** Piezoionic sensor attached to the throat for vocalization recognition. **d** Sensor voltage output signatures measuring vocal cord vibration when pronouncing English alphabet and (**e**) words. **f** Piezoionic sensor attached to the wrist for pulse signal monitoring. **g** Pulse wave signals generated by the piezoionic sensor. **h** Piezoionic eutectogel electrodes attached to the wrists for electrocardiographic (ECG) signal acquisition. **i** Comparison of ECG signals recorded using DES70 as electrodes against commercial hydrogel electrodes, showing comparable signal strength and fidelity.

A variety of physiological processes generate both mechanical and bioelectrical signals, including subtle pressure stimuli such as vocal cord vibrations and arterial pulse waves, as well as electrical signals exemplified by ECG, which are widely used for human signal monitoring (Fig. 5a). Benefiting from the intrinsic piezoionic response and favorable electrical properties of the eutectogels, two flexible device configurations were designed to enable the detection of both mechanical and bioelectrical signals in a skin-attachable platform (Fig. 5b).

Owing to their high piezoionic sensitivity, the eutectogel-based epidermal sensors are capable of resolving weak mechanical inputs. As a representative example, the sensor was attached to the throat with the aid of a skin-adhesive PU film to monitor vocal cord vibrations during speech (Fig. 5c). When different English alphabets and numbers were articulated, the eutectogel sensor produced distinct voltage waveforms corresponding to the vibration patterns of the vocal cords. As shown in Fig. 5d, e and Supplementary Fig. 22, each alphabet and number exhibit a characteristic waveform signature, enabling reliable subsequent classification. Furthermore, the highly similar signal waveforms obtained for the same word spoken by different individuals, including two male and two female participants, confirm the reliability of the sensor in identifying vocal cord vibration patterns (Supplementary Figs. 23, 24). This high-fidelity capture of subtle vibrational features highlights the potential of the piezoionic sensors for speech-related human-machine interfaces. In addition to vocal signal monitoring, the epidermal sensor was applied to arterial pulse diagnostics by attachment to the wrist (Fig. 5f). The resulting voltage waveforms resolve key features of the arterial pulse, including the percussion (P) wave, tidal (T) wave, and dicrotic (D) wave (Fig. 5g). These results demonstrate that piezoionic eutectogels represent a promising platform for pulse signal monitoring.

In addition to the aforementioned self-powered sensing applications, the eutectogels can be further employed for electrophysiological monitoring due to their excellent ionic conductivity. Furthermore, DES70, combining mechanical compliance with high ionic conductivity, was employed as soft, skin-compatible electrodes for ECG acquisition (Fig. 5h). As shown in Fig. 5i, the recorded ECG signals display well-defined P, Q, R, S, and T waves, with a stable heart rate of approximately 85 beats per minute under resting conditions. The signal quality and signal-to-noise ratio are comparable to those obtained using commercial hydrogel electrodes (Supplementary Fig. 25), and long-term stability has also been confirmed (Supplementary Fig. 26).

Collectively, these demonstrations verify that the DES-PVA eutectogel biosensor provides multifunctional capabilities for the accurate monitoring of diverse mechanical and bioelectrical physiological signals. The combination of high sensitivity, mechanical compliance, and stable bioelectrical interfacing indicates strong potential for the piezoionic eutectogel sensor as a versatile platform for wearable health monitoring systems.

## Discussion

This work demonstrates a piezoionic eutectogel sensor capable of effective mechanoelectrical signal generation and the epidermal sensing of human physiological signals. The proposed DES-PVA eutectogel exhibits excellent biocompatibility as confirmed by in vitro cytotoxicity assays. Under mechanical stimulation, the eutectogel sensor delivers a piezoionic voltage of up to 72 mV with a highly stable baseline through repeated load cycles. This level of piezoionic output and mechanical robustness is achieved by hydrogen-bond regulated selective ion transport. Multiscale analyses, including DFT, MD, and 2DCOS, elucidate that immobilization of Ch^+^ while allowing Cl^–^ to diffuse enhances piezoionic performance. For demonstrating practical applications, the eutectogel sensor in the form of a high-performance epidermal device monitors both mechanical and bioelectrical human physiology, with a high signal quality. Overall, this work presents a compliant eutectogel sensor that combines biological safety, piezoionic transduction, and reliable physiological signal acquisition, offering a practical approach toward self-powered epidermal bioelectronics.

## Methods

### Materials

Polyvinyl alcohol (PVA 1799) and choline chloride (ChCl, 98%) were purchased from Macklin Biochemical Co., Ltd. Glycerol (≥ 99.5%) was obtained from Aladdin Industrial Corp. All chemicals were used as received without further purification. The Commercial gel patch was purchased from Guangzhou Zierkang Health Industry Co., Ltd. Ultra-thin PU film with single-sided adhesive was purchased from 3 M Company. Deionized water with a resistivity of 18.2 MΩ·cm was used throughout all experiments. Human prostate stroma myofibroblast (WPMY-1) cells were obtained from American Type Culture Collection (ATCC).

### Preparation of PVA solution and ChCl-glycerol DES

The aqueous PVA solution with 15 wt% PVA content was prepared as follows: 15 g of PVA1799 was added to a round-bottom flask with 85 mL deionized water, followed by continuous stirring at 90 °C for 6 h until a homogeneous and transparent solution was obtained. For the preparation of DES with of 1:2 ChCl-glycerol molar ratio, 8.62 g of ChCl and 11.38 g of glycerol were mixed at a round-bottom flask and stirred vigorously at 40 °C for 30 min until a clear and uniform liquid was formed. Both the PVA solution and the ChCl-glycerol DES were allowed to cool to room temperature and reserved for subsequent use.

### Preparation of DES-PVA eutectogel

The as-prepared PVA solution was reheated to 90°C under static conditions for 30 min, followed by continuous stirring for 30 min. Subsequently, varying amounts of the DES were slowly added dropwise to the resulting PVA solution, and then the mixture was further stirred for 1 h to ensure that a homogeneous DES-PVA solution had formed. The resulting solution was transferred into a vacuum chamber to remove residual air. Then, the obtained solution was subsequently injected into glass molds and dried in an oven at 80 °C for 48 h.

### Characterizations and measurements

The surface morphology of the eutectogels was observed using a field emission scanning electron microscope (FESEM, Thermo Fisher Apreo 2). The structure of as-prepared samples was examined by X-ray diffraction (XRD, Bruker Discover 8 diffractometer) with Cu Kα irradiation (λ = 1.5406 Å) at 300 mA and 40 kV. The Raman spectroscopy was performed on a laser Raman spectrometer (WITec, Alpha 300 R) with a 633 nm laser and recorded in the range of 4000-400 cm^–1^. Fourier transform infrared spectroscopy (FT-IR) and corresponding mapping were conducted using a Bruker VECTOR-22 spectrometer. Mechanical characterization was carried out using a universal testing machine (Instron 68SC). Tensile behavior was evaluated by recording stress-strain responses and cyclic loading and unloading curves at a constant crosshead speed of 50 mm min^–1^. Monotonic compression tests were conducted at a loading rate of 0.5 mm min^–1^, while cyclic compression tests were performed at 50 mm min^–1^. All measurements were performed under ambient laboratory conditions. Rectangular specimens with dimensions of 10 × 50 mm and a thickness of 1 mm were used for all mechanical tests.

For the piezoionic measure, the pressure was obtained and monitored using a homemade Mark 10, which was connected and controlled by a PC using MATLAB software. The electrical current, open circuit voltage, and load voltage were detected by using a data acquisition instrument (Keithley 2700, USA). Power output was calculated by multiplying current and voltage across the external load, which was specifically matched to the internal resistance of the eutectogel measured under direct current. Electrochemical impedance spectroscopy (EIS) was collected at room temperature using an electrochemical workstation (PARSTAT2273) in a 0.1 Hz–1 MHz frequency range with a 10 mV AC perturbation to evaluate the intrinsic ionic conductivity relevant to both piezoionic conduction and ECG monitoring. The ionic conductivity (*σ*) is calculated according following equation: *σ* = *L/RS*, where *L* and *S* are the thickness and cross-section area of the cell, *R* is the resistance from EIS. For recording ECG signals, a BIOPAC monitoring system (MP160) was employed, and the commercial gel patch used was the reference patch. For physiological monitoring, each participant gave informed written consent.

### Cytotoxicity tests

To assess the biocompatibility of the eutectogels, WPMY-1 cells were seeded in 6 well plates with the initial density of 1 × 10^6^ cell per well. For comparison, three additional ionic gels were prepared by combining PVA with three commonly used ILs, including EMIMBF₄, EMIMTFSI, and EMPTFSI. Eutectogels containing 10 wt% and 50 wt% DES contents, together with control PVA-IL gels containing 10 wt% EMIMBF₄, EMIMTFSI, and EMPTFSI, were prepared as standardized discs with a radius of 5 mm and a thickness of 1 mm. A cell culture well without gel sample served as the negative control for subsequent comparative analysis. After 24 h of incubation, cell viability was evaluated by confocal laser scanning microscopy after dual fluorescence Calcein AM/PI staining.

### Reporting summary

Further information on research design is available in the Nature Portfolio Reporting Summary linked to this article.

## Supplementary information

Supplementary Informatiion

Reporting Summary

Peer Review File

## Source data

Source Data

## Data Availability

All data supporting the findings of this study are available within the article and its supplementary file. Any additional requests for information can be directed to, and will be fulfilled by, the corresponding authors. Source data are provided in this paper.
