# Supplementary material for: Biocompatible piezoionic eutectogel sensor for self-powered physiological monitoring
Source: Nat Commun. 2026 Sep 23;17:11053. doi: 10.1038/s41467-026-77390-7 (PMC13691130; doi:10.1038/s41467-026-77390-7)
Supplement: Supplementary file 1 [file 41467_2026_77390_MOESM1_ESM.pdf]

Supplementary Information

Biocompatible Piezoionic Eutectogel Sensor for Self-Powered Physiological Monitoring

*Bo Wu[1,2], Xuejie Liu[1,2], Zhiheng Zeng[1,2], Bolong Li[1,2], Haoyu Fang[1,2], Derek Ho[1,2,3]**

[1] Department of Materials Science and Engineering, City University of Hong Kong, Kowloon 999077, Hong Kong, China.

[2] Hong Kong Centre for Cerebro-cardiovascular Health Engineering, N.T. 999077, Hong Kong, China.

[3] Institute of Digital Medicine, City University of Hong Kong, Kowloon 999077, Hong Kong, China.

* Corresponding author: Derek Ho (derekho@cityu.edu.hk)

Content List:

1. Supplementary Note
2. Supplementary Figures
3. References

Supplementary Notes

Supplementary Note 1. Necessity of dropwise addition during the preparation of the DES–PVA composite solution

A more detailed analysis reveals that the DES–PVA system exhibits pronounced pathway-dependent behavior during mixing, rather than being determined solely by its final composition. Experimental observations indicate that both direct premixing of the DES with the aqueous PVA solution followed by heating and one-step addition of the DES into the PVA solution at 90 °C consistently led to the formation of abundant semitransparent aggregates (Supplementary Fig. 2). Once formed, these aggregates cannot be eliminated by prolonged heating or intensified stirring, indicating that the system becomes kinetically trapped in an aggregated nonequilibrium state. This behavior highlights the strong sensitivity of the DES–PVA system to the rate and manner of solvent environment evolution, which is characteristic of pathway-dependent polymer solution systems.

From a mechanistic perspective, direct mixing or abrupt addition introduces the DES locally at a high effective concentration. Under these conditions, chloride anions (Cl^-) and glycerol molecules engage in strong competitive hydrogen bonding with PVA hydroxyl groups, rapidly disrupting the original water-dominated solvation shell of the polymer chains[1–3]. This sudden deterioration in solvent quality drives the system across the solubility stability boundary of PVA on a very short timescale. At elevated temperature, where polymer chains exhibit high conformational mobility, this transition induces rapid chain contraction accompanied by enhanced intermolecular association and hydrogen-bond reorganization, leading to the emergence of localized PVA-rich amorphous domains[4]. Although these aggregates are not thermodynamically favored equilibrium structures, their formation under strongly nonequilibrium conditions renders them kinetically stabilized and difficult to redisperse, thereby impeding the development of a continuous and homogeneous eutectogel network.

In contrast, dropwise addition of the DES solution into the PVA solution at 90°C effectively suppresses instantaneous spatial and temporal concentration gradients, transforming an abrupt solvent-quality transition into a gradual and controllable process. This regulated introduction enables polymer conformational relaxation and hydrogen-bond reorganization to proceed concurrently, thereby preventing rapid chain collapse and macroscopic phase separation[5]. As a result, the system follows a well-defined kinetic pathway toward a homogeneous mixed state, yielding a transparent and uniform precursor solution that is essential for constructing eutectogels with a well-integrated polymer framework and continuous, percolated ionic transport networks.

Supplementary Note 2. Basic principle of Raman mapping for identifying phase separation

The fundamental basis of Raman mapping for identifying phase separation lies in its ability to spatially resolve chemically specific information with high spatial resolution across heterogeneous polymer-ionic systems. In a typical Raman mapping experiment, a predefined two-dimensional grid is established over a selected region of the sample, and Raman spectra are acquired point by point at each spatial coordinate within the grid. During subsequent data analysis, characteristic Raman bands with well-defined chemical assignments are selected, and the intensity of a given band at each position is taken to reflect the local enrichment or depletion of the corresponding component. In the resulting mapping images, regions of higher band intensity appear as brighter contrast, whereas areas of lower intensity are represented by darker contrast. By comparing the spatial distributions of characteristic Raman bands across the mapped area, compositional heterogeneities within the polymer network and ionic domains can be directly visualized. When phase separation occurs, the distinct Raman signatures of different phases manifest as spatially segregated domains or continuous intensity gradients. Accordingly, Raman mapping enables not only the identification of phase separation but also the characterization of its morphology, characteristic length scale, and spatial continuity at the microscale.

In this work, Raman mapping was further employed to resolve the spatial distribution of individual DES components within the PVA network, thereby enabling a mechanistically informed assessment of potential phase separation. Relative to glycerol and PVA, choline cations (Ch^+) exhibit distinct Raman fingerprints that permit unambiguous differentiation. Specifically, the C-N vibrational mode associated with the choline group gives rise to characteristic Raman bands in the low-wavenumber region, typically centered around ~715 cm^{-1}, while the C-H stretching feature of the N-CH_3 groups appears in the range of approximately 3030–3040 cm^{-1} and can be used as a clear spectral marker for choline-containing species[6, 7]. In contrast, neither PVA nor glycerol shows noticeable Raman signals in this high-wavenumber region, allowing this band to be confidently assigned to Ch^+. These two features therefore serve as chemically specific Raman markers for tracking the spatial distribution of choline-containing DES within the gel matrix. Therefore, intensity maps constructed from the integrated C-N and N-CH_3 bands directly visualize the local enrichment or depletion of choline ions across the selected region.

Supplementary Note 3. Density functional theory (DFT) calculation

All DFT calculations were performed using the Gaussian 16 software package. The molecular geometries of all isolated species and combined complexes were fully optimized in their electronic ground states at the B3LYP/6-311G+(d, p) level of theory, with the Grimme D3 dispersion correction combined with Becke Johnson damping applied to account for long-range van der Waals interactions that are critical for describing hydrogen-bonded and weakly coordinated ionic environments. Geometry optimizations were conducted without symmetry constraints, and the convergence criteria were set to the default tight thresholds implemented in Gaussian. Vibrational frequency analyses were performed at the same level of theory to verify that all optimized structures correspond to true local minima on the potential energy surface, as confirmed by the absence of imaginary frequencies. Following geometry optimization, single-point energy calculations were carried out to obtain accurate total electronic energies and electrostatic potential distributions for each molecular system.

The molecular ESP, $V(r)$, was calculated on the van der Waals surface of each molecule according to the standard expression[8]:

$$V(r) = \sum_{A} \frac{Z_A}{|R_A - r|} - \int \frac{\rho}{|r' - r|} dr'$$

where Z_A and R_A denote the nuclear charge and position of atom A, respectively, and $\rho(r')$ represents the electronic density. The electrostatic potential values were calculated in atomic units and mapped onto the molecular van der Waals surface to visualize regions of electrostatic enrichment and depletion, thereby providing a molecular-level descriptor for ion-specific coordination preferences and hydrogen-bond-regulated electrostatic asymmetry.

The binding energy (ΔE) was determined according to the formula[9]:

$$\Delta E = E_{AB} - (E_A + E_B)$$

where E_{AB} is the total electronic energy of the optimized complex, and E_A and E_B correspond to the total electronic energies of the isolated components in their respective optimized geometries. A negative value of ΔE indicates a thermodynamically favorable interaction. All reported binding energies were derived from dispersion-corrected total energies to ensure a consistent and reliable description of noncovalent interactions governing the stability of hydrogen-bonded and ion-associated configurations within the DES–PVA system.

Supplementary Note 4. Molecular dynamics (MD) simulation

MD simulations were performed using the GROMACS simulation package in conjunction with the OPLS-AA all-atom force field. Bonded parameters and Lennard Jones interaction parameters were generated using the Autoff toolkit, while restrained electrostatic potential atomic charges were derived from quantum chemical calculations and assigned using the Multiwfn 3.8 program[10]. This charge derivation scheme provides a physically consistent and transferable description of electrostatic interactions within the hydrogen-bonded and ionic environments characteristic of the eutectogel system, thereby enabling reliable representation of both ion-specific coordination and long-range Coulombic effects that underpin mobility differentiation. Nonbonded interactions were described using the Lennard Jones potential[11], expressed as:

$$V(r) = 4\epsilon\left[\left(\frac{\sigma}{r}\right)^{12} - \left(\frac{\sigma}{r}\right)^{6}\right]$$

where r denotes the interatomic separation, ϵ represents the depth of the potential well, and σ corresponds to the finite distance at which the interparticle interaction energy is zero. The cutoff distance for the Lennard Jones potential was set to 12 Å. Long-range Coulombic interactions were treated using the particle mesh method to ensure accurate evaluation of electrostatic forces beyond the real-space cutoff.

Initial configurations of the periodic simulation boxes were constructed using the PACKMOL package[12], in which all polymer chains, ions, and solvent molecules were randomly packed into a cubic simulation cell with a side length of 10 nm. Periodic boundary conditions were applied in all three spatial directions. Prior to production runs, the system was subjected to energy minimization using the conjugate gradient algorithm to remove unfavorable atomic contacts and to relax the initial structure. The system was subsequently equilibrated under an isothermal isobaric ensemble for 10 ns at a temperature of 300 K and a pressure of 1 atm. Temperature and pressure were regulated using standard thermostat and barostat coupling schemes to maintain stable thermodynamic conditions.

To construct the mechanically deformed eutectogel model, the equilibrated system was replicated three times along the axial direction to generate an elongated simulation cell. A time-dependent external force was then applied to the central region of the model to mimic compressive mechanical loading, with a force constant of 1000 kJ mol^{-1} m^{-2}. Following mechanical perturbation, the simulation was continued under a canonical ensemble for an additional 20 ns, during which structural and dynamical trajectories were collected for subsequent analysis. Radial distribution functions, g(r), were computed to quantify the local coordination environments and hydrogen-bond-regulated interactions between polymer functional groups and ionic species under both undeformed and mechanically loaded conditions, thereby establishing a direct correlation between mechanical deformation, molecular-scale reorganization, and ion transport behavior.

Supplementary Note 5. Two-dimensional correlation spectroscopy (2DCOS) analysis

2DCOS was employed to analyze the pressure-dependent FTIR spectra and to resolve overlapping O-H stretching vibrations[13, 14]. In our eutectogel, multiple hydrogen-bonding interactions among PVA, Cl^-, Ch^+, and glycerol molecules give rise to a broad and highly overlapped spectral envelope within the range of 2950–3600 cm^{-1}. As a result, conventional one-dimensional (1D) FTIR spectra do not enable the direct identification of individual spectral components or their responses to external perturbation. To address this limitation, 2DCOS analysis was performed using the 2Dshige software, which processes a series of dynamic spectra collected under controlled pressure variation. The method generates synchronous and asynchronous correlation maps based on the Hilbert transform of the spectral dataset[15]. The synchronous map reflects the overall intensity variations of spectral features with respect to the external perturbation, whereas the asynchronous map provides information on the sequential order of spectral changes, enabling the resolution of overlapped bands[16, 17].

In the present study, the characteristic wavenumbers were not assigned based on visual inspection of the 1D spectra, but were objectively identified from the 2DCOS results. Specifically, the diagonal autopeaks in the synchronous map were used to determine the spectral regions that exhibit the highest sensitivity to pressure. In addition, prominent cross-peaks observed in the asynchronous map were used to establish correlations between different vibrational modes. Based on these criteria, four representative wavenumbers at 2998, 3278, 3291, and 3544 cm^{-1} were extracted directly from the correlation maps. These spectral positions correspond to distinct molecular environments within the DES-PVA network. The bands at 3278 and 3291 cm^{-1} are attributed to hydrogen-bonded O-H stretching modes, corresponding to glycerol-Cl^- and PVA-Ch^+ interactions, respectively. The band at 3544 cm^{-1} is assigned to relatively free O-H groups, while the band at 2998 cm^{-1} originates from C-H stretching vibrations. Although some of these features are not clearly distinguishable in the original 1D spectra due to band overlap, their presence is revealed through their strong correlation responses in the 2DCOS maps. The sequential response of these vibrational modes to mechanical perturbation was further analyzed using Noda's rule[18]. When the synchronous and asynchronous cross-peaks share the same sign, the change at v_1 precedes that at v_2; otherwise, the reverse sequence applies. This analysis enables identification of the relative sensitivity of different functional groups to pressure and provides insight into the evolution of hydrogen-bonding interactions within the eutectogel.

Overall, the 2DCOS analysis demonstrates that pressure induces a redistribution of hydrogen-bonding interactions within the DES-PVA network, thereby modulating the local ionic environment. This process plays a critical role in generating the asymmetric ion transport behavior responsible for the observed piezoionic response.

Supplementary Figures

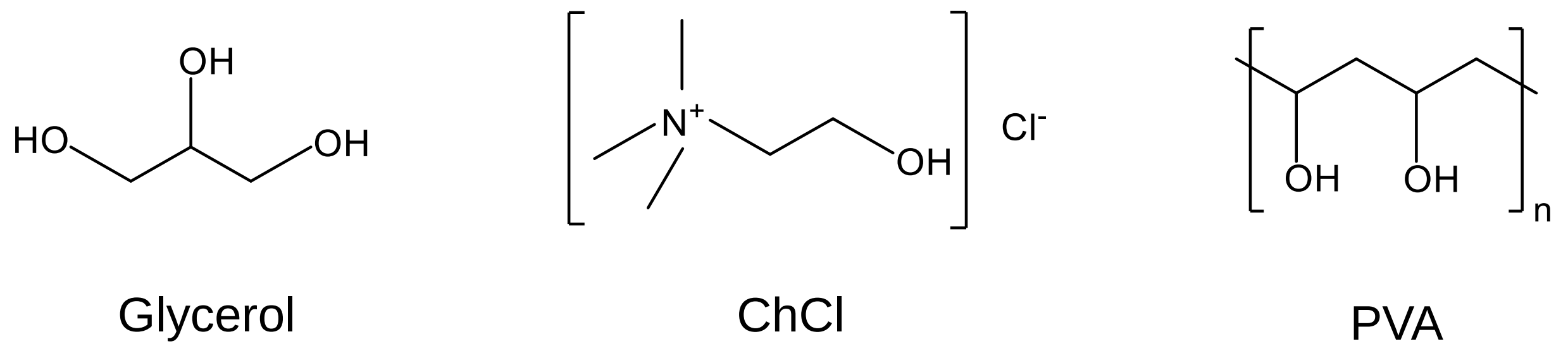

Supplementary Fig. 1 The chemical structure of glycerol, ChCl, and PVA chain.

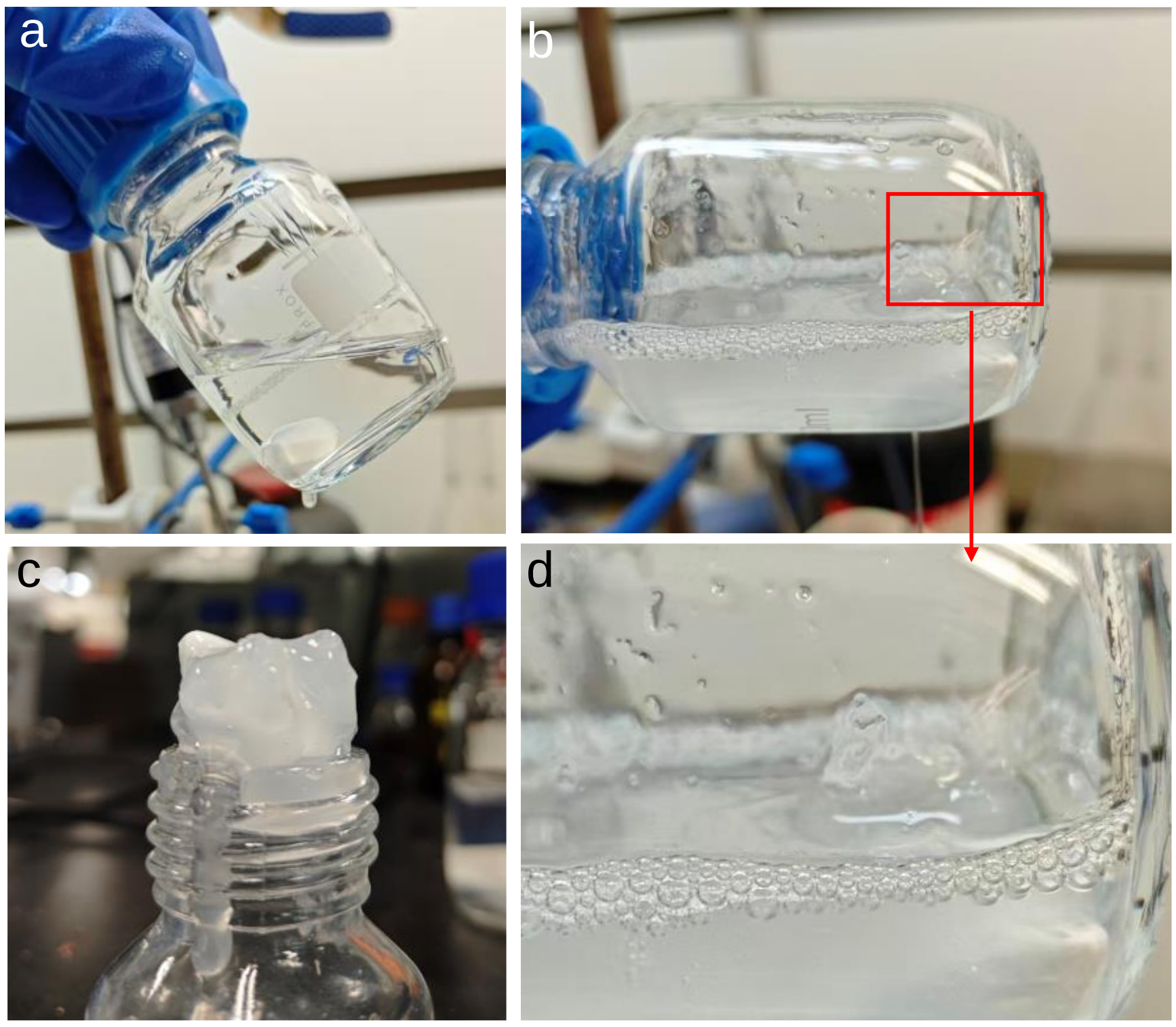

Supplementary Fig. 2 (a) Photograph of the composite solution obtained by the dropwise addition of DES, demonstrating good compatibility. (b-d) Photographs of the mixture obtained by mixing DES and PVA at room temperature followed by co-heating under 90℃, with the agglomeration indicating poor compatibility.

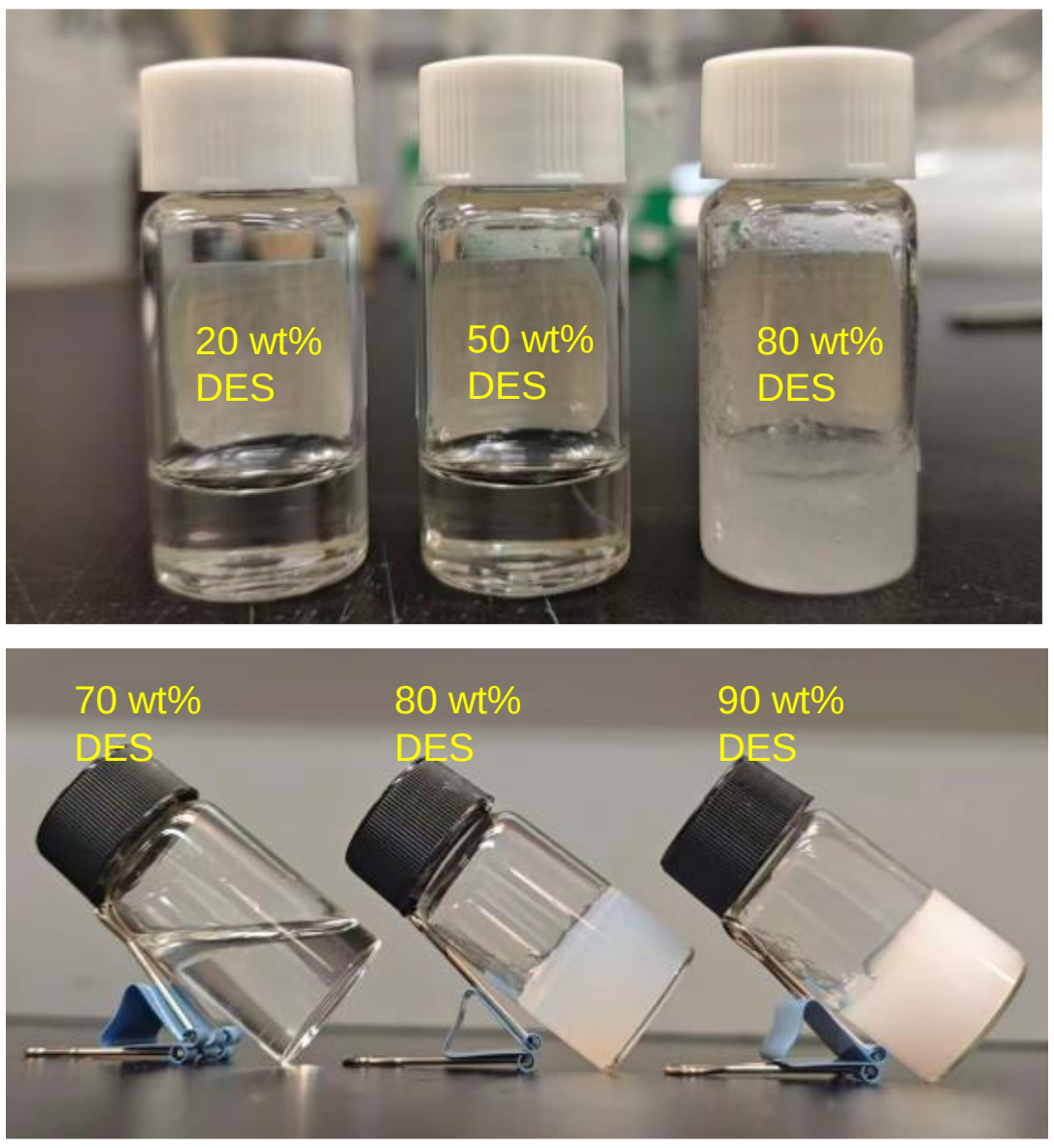

Supplementary Fig. 3. Photograph of PVA-DES precursor solutions with different DES contents after dissolving at 90℃.

It should be noted that the DES-PVA solutions exhibit distinct phase behaviors depending on both ChCl content and temperature. At a high DES content of 80 wt%, the system showed pronounced microphase separation. This phase separation is accompanied by rapid gelation of the solution, which is unfavorable for the subsequent film-forming process. In contrast, a transparent and homogeneous solution can be obtained at a DES content of 70 wt%.

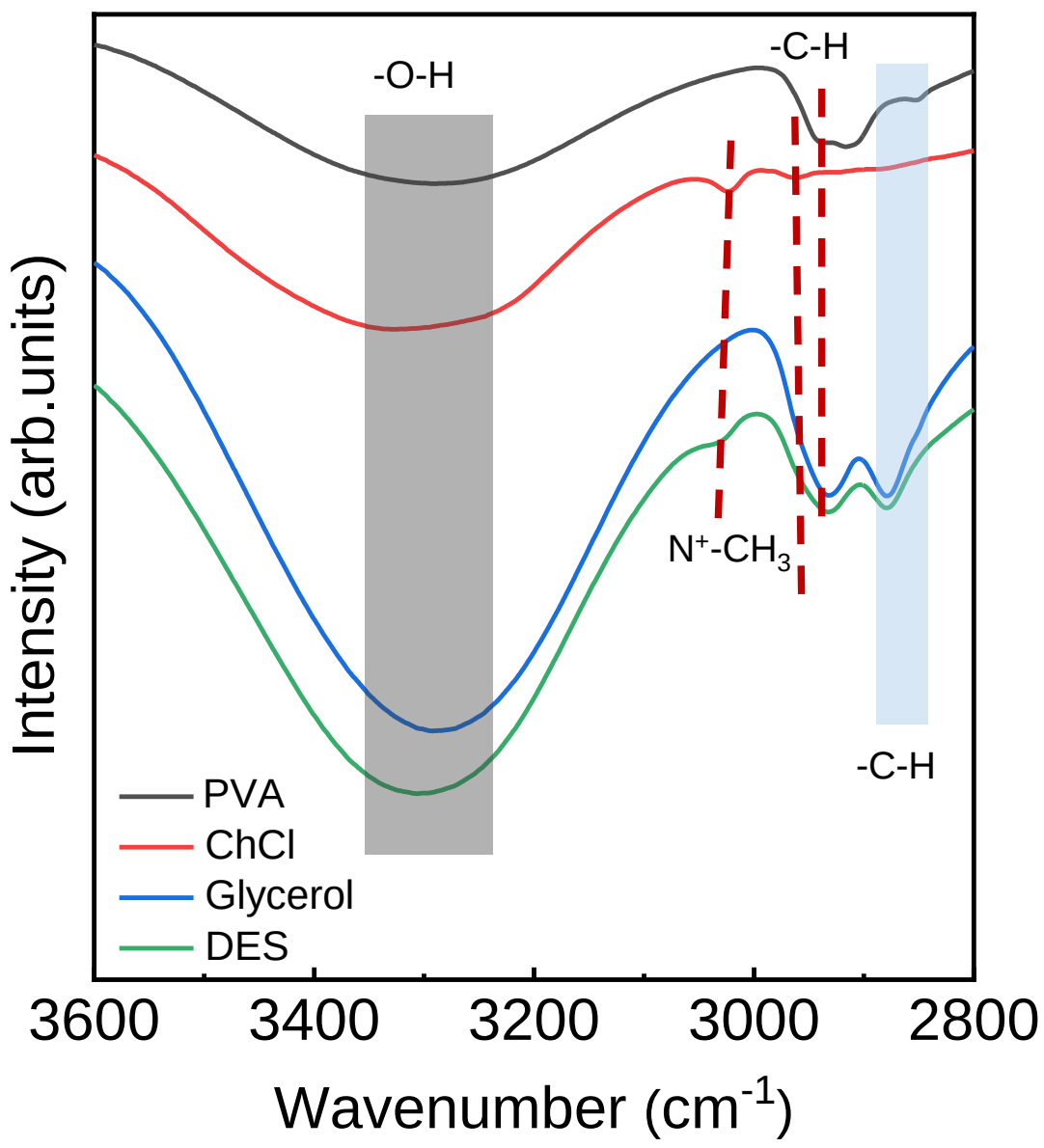

Supplementary Fig. 4 FTIR spectra of PVA, ChCl, glycerol and the DES.

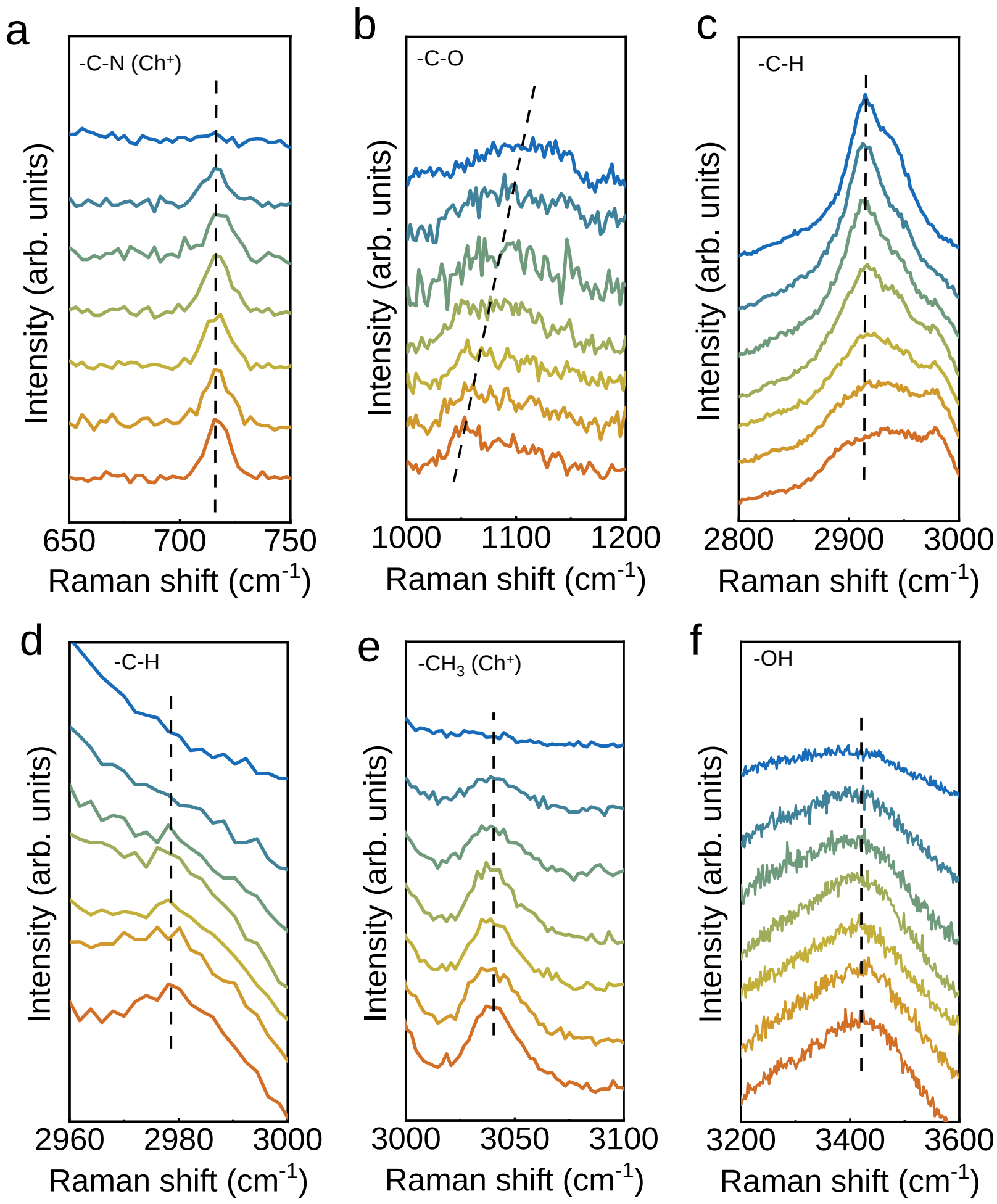

Supplementary Fig. 5 Raman spectra corresponding to the characteristic vibrational bands of the selected functional groups. (a) C-N groups of Ch^+. (b) C-O groups. (c) -C-H groups. (d) -C-H groups. (e) $-CH_3$ groups of Ch^+. (f) -OH groups.

As the DES content increases, the Raman spectra display systematic and correlated variations across multiple spectral regions, indicating progressive incorporation of DES components accompanied by the reorganization of the PVA network. The characteristic bands associated with choline become progressively more prominent, including the C-N vibration near 715 cm^{-1} and the $N-CH_3$ stretching mode around 3040 cm^{-1} (Supplementary Figs. 5a and 5e). Notably, the positions of these peaks remain essentially unchanged, indicating that the incorporation of Ch^+ does not perturb its local covalent configuration. In parallel, the band centered at approximately 1100 cm^{-1}, assigned to C-O vibrations, exhibits both increased intensity and spectral broadening (Supplementary Fig. 5b). This behavior is consistent with a broader distribution of hydrogen-bonding interactions and solvation environments involving PVA chains and glycerol molecules. In the C-H stretching region, the band at approximately 2920 cm^{-1}, associated with pristine PVA environments, gradually decreases with

increasing DES content, whereas the band near 2980 cm^{-1} can be attributed to $-CH_3$ groups from ChCl (Supplementary Figs. 5c and 5d). These opposite trends indicate that the increasing DES fraction not only alters the relative spectral contributions of the constituent components, but also reflects enhanced chain relaxation and segmental mobility within the eutectogel network. At higher wavenumbers, the broad -OH stretching envelope centered around 3400 cm^{-1} becomes both more intense and significantly wider (Supplementary Fig. 5f), suggesting an expanded distribution of hydrogen-bonding configurations.

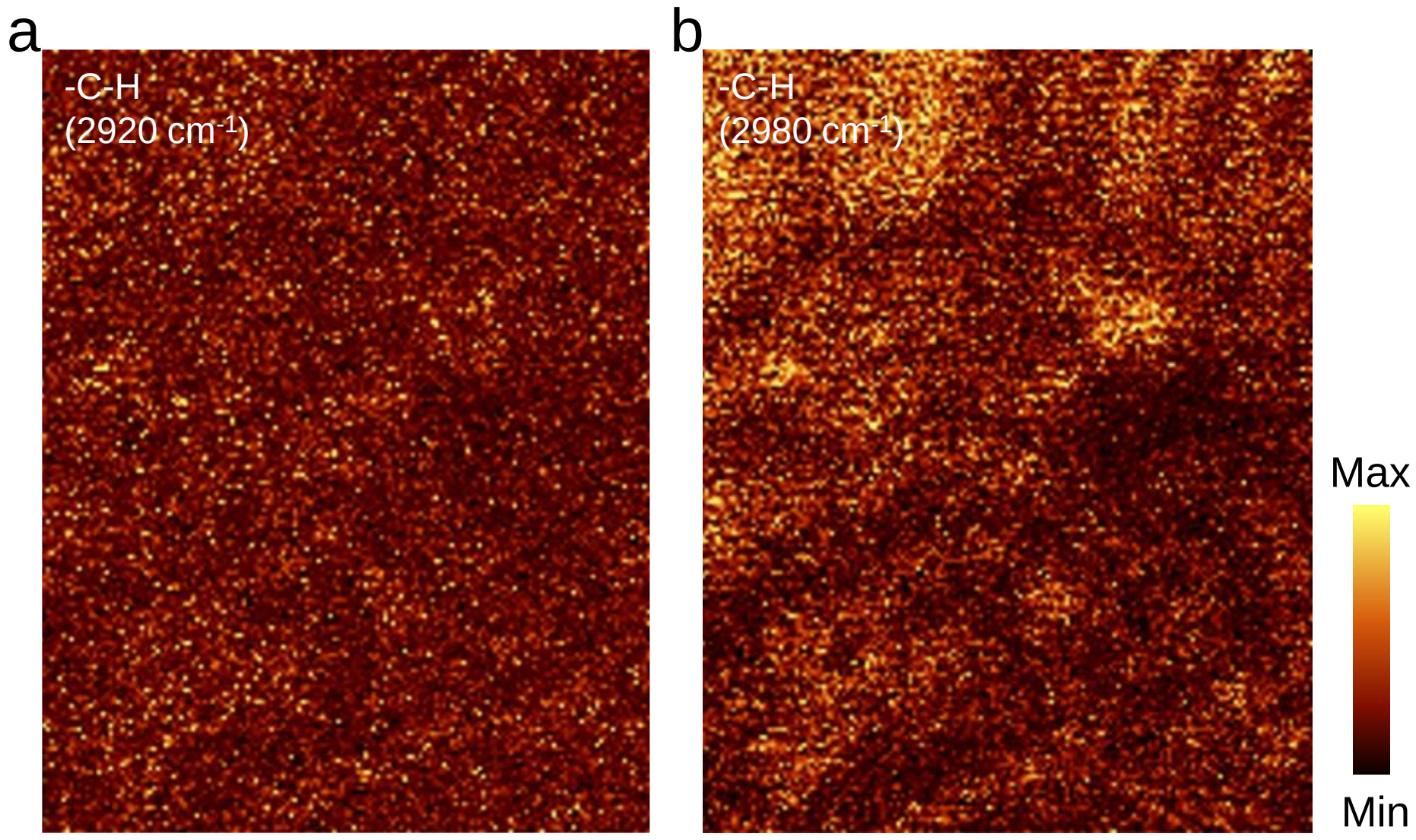

Supplementary Fig. 6 Raman mapping images corresponding to characteristic functional groups (C-H) in the eutectogel. n = 3 independent samples.

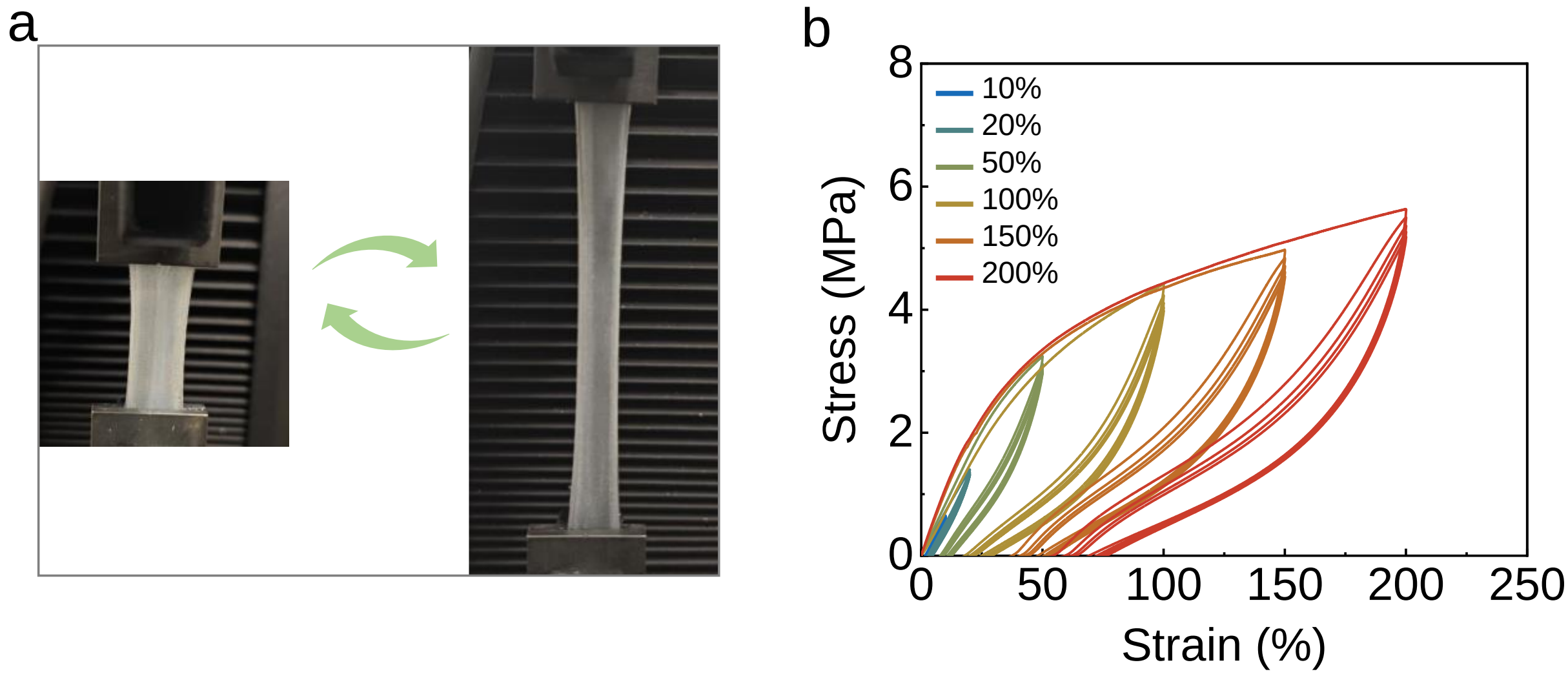

Supplementary Fig. 7 (a) Photographs of the mechanical testing. (b) Cyclic mechanical testing of the eutectogels under different tensile strain.

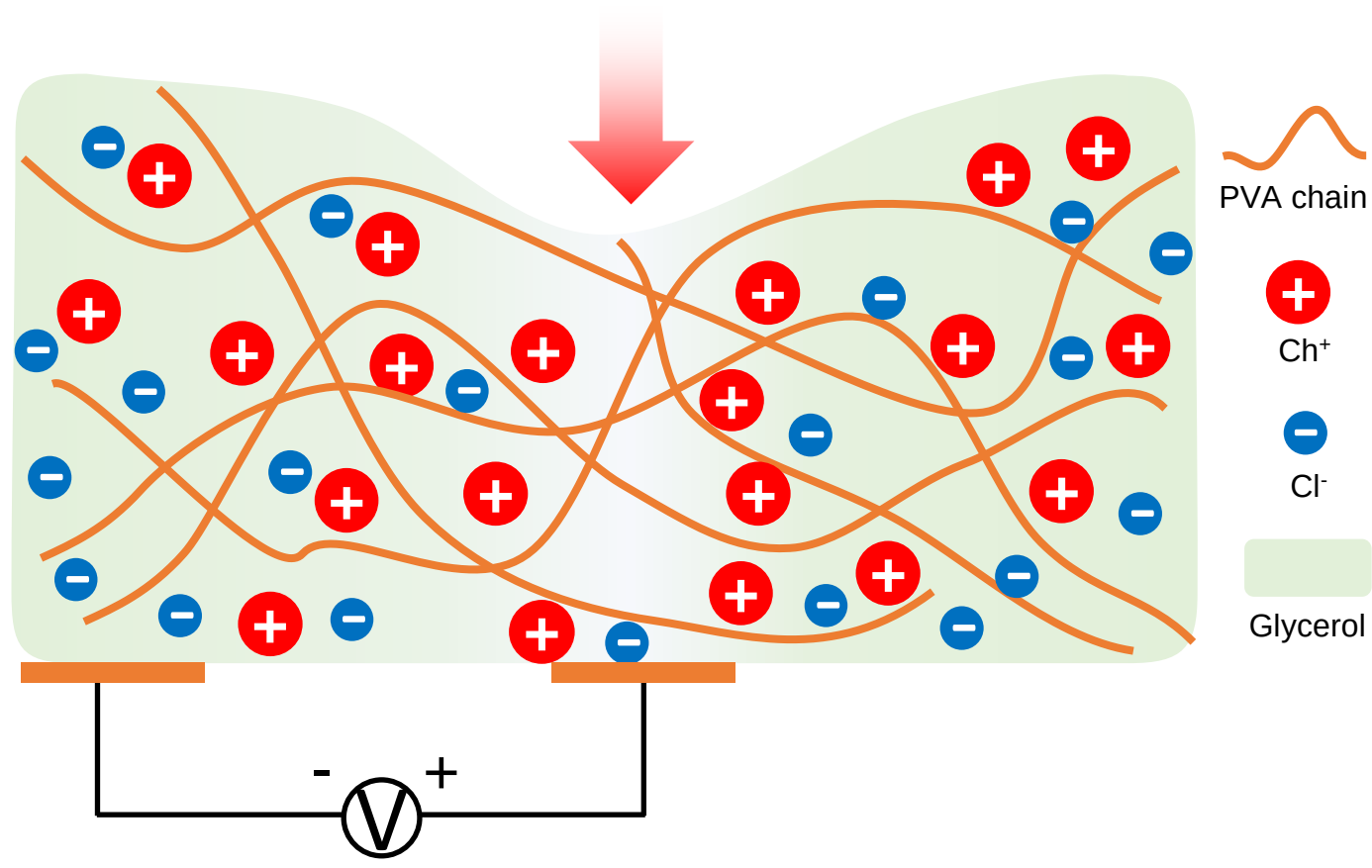

Supplementary Fig. 8 Schematic of the eutectogel under pressure from an indentation test.

During piezoionic voltage and current measurements, two surface electrodes were placed on the eutectogel with an interelectrode distance of 3 cm. Gold-plated copper foil electrodes with a radius of 5 mm were employed. During testing, the loading head of the Mark-10 force gauge was precisely aligned with the center of one electrode, and the loading rate was maintained at 50 mm min^{-1} throughout the measurements.

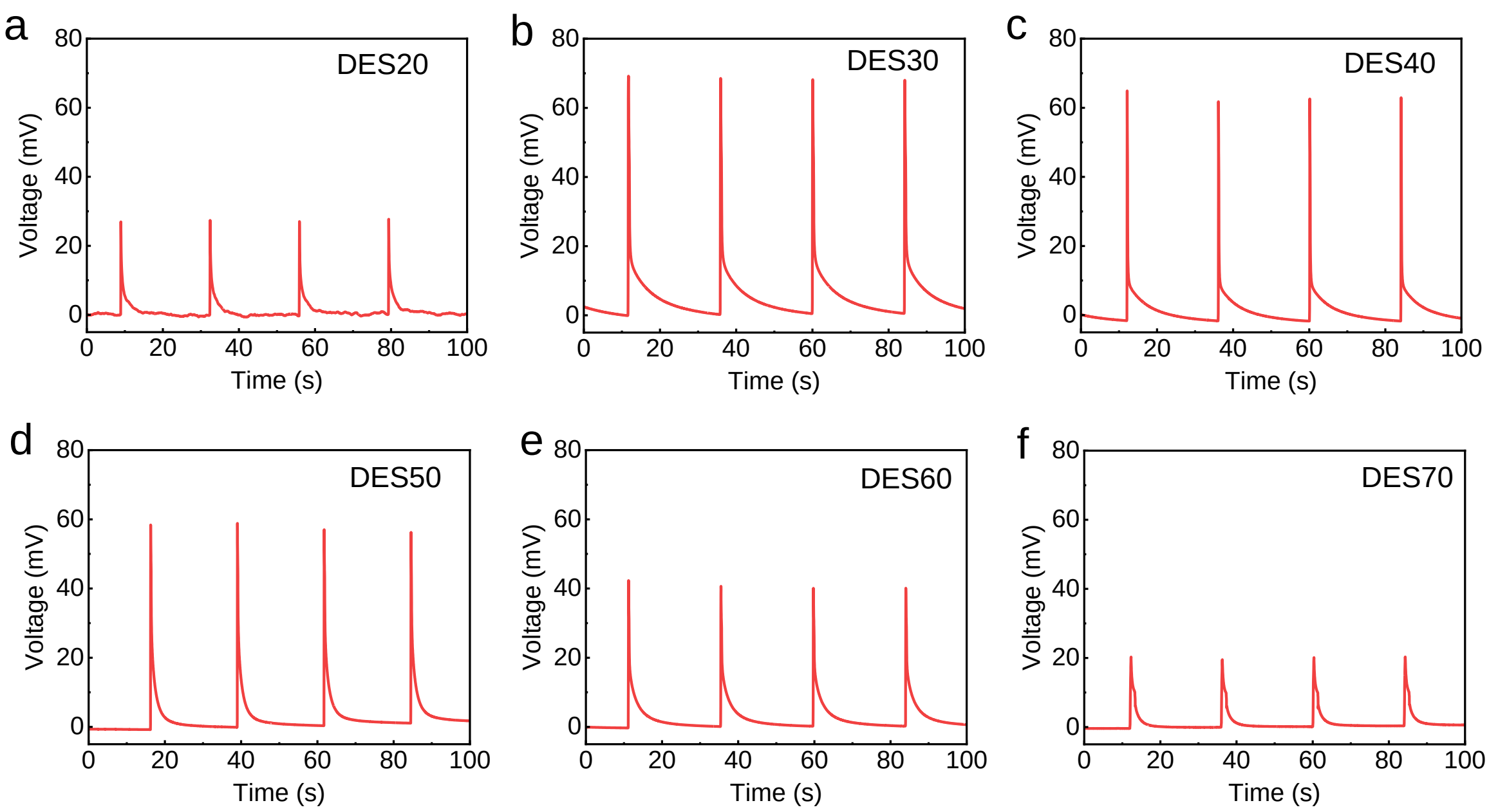

Supplementary Fig. 9 Piezoionic voltage responses of eutectogels with different DES contents.

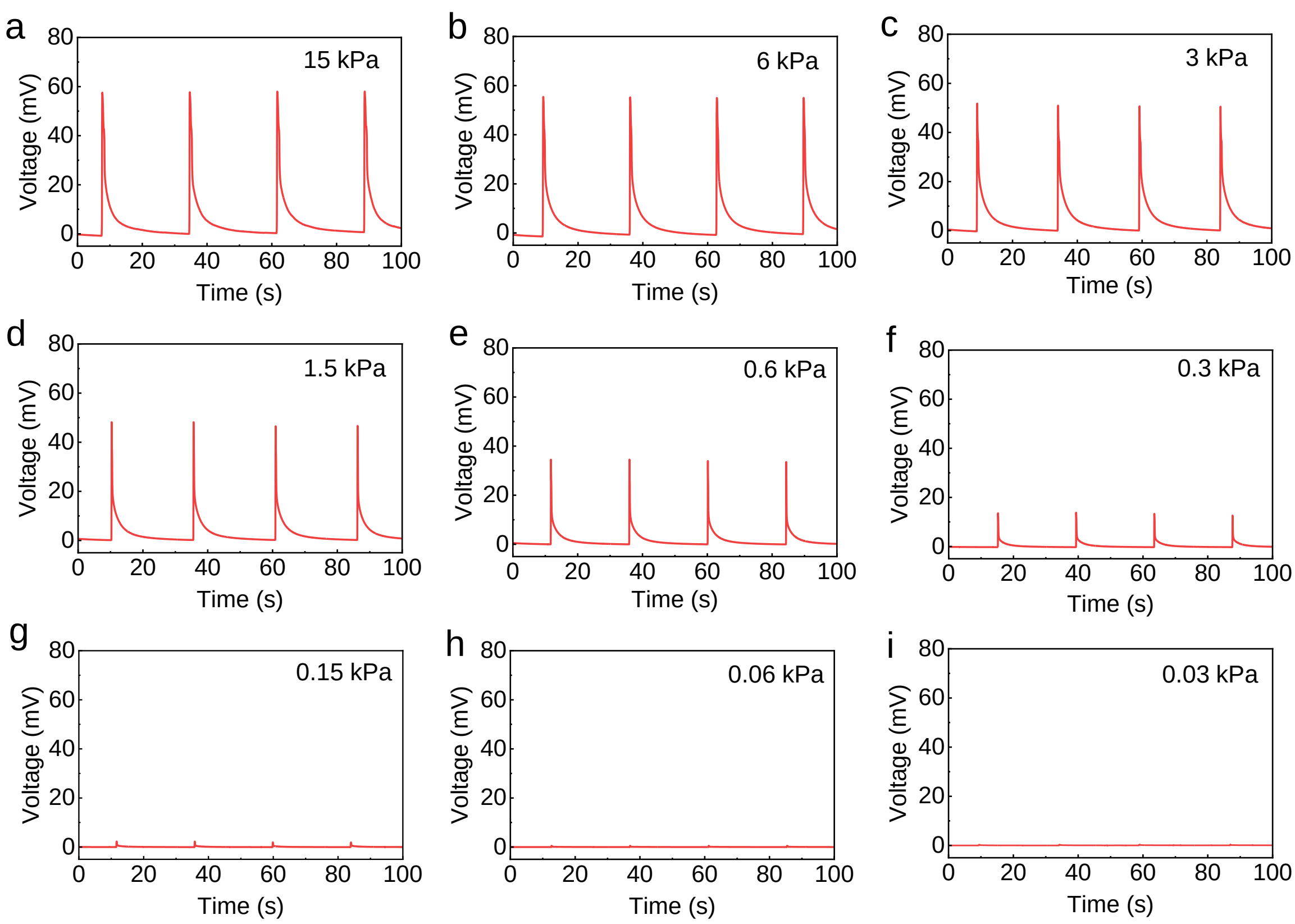

Supplementary Fig. 10 Piezoionic voltage responses under different pressure.

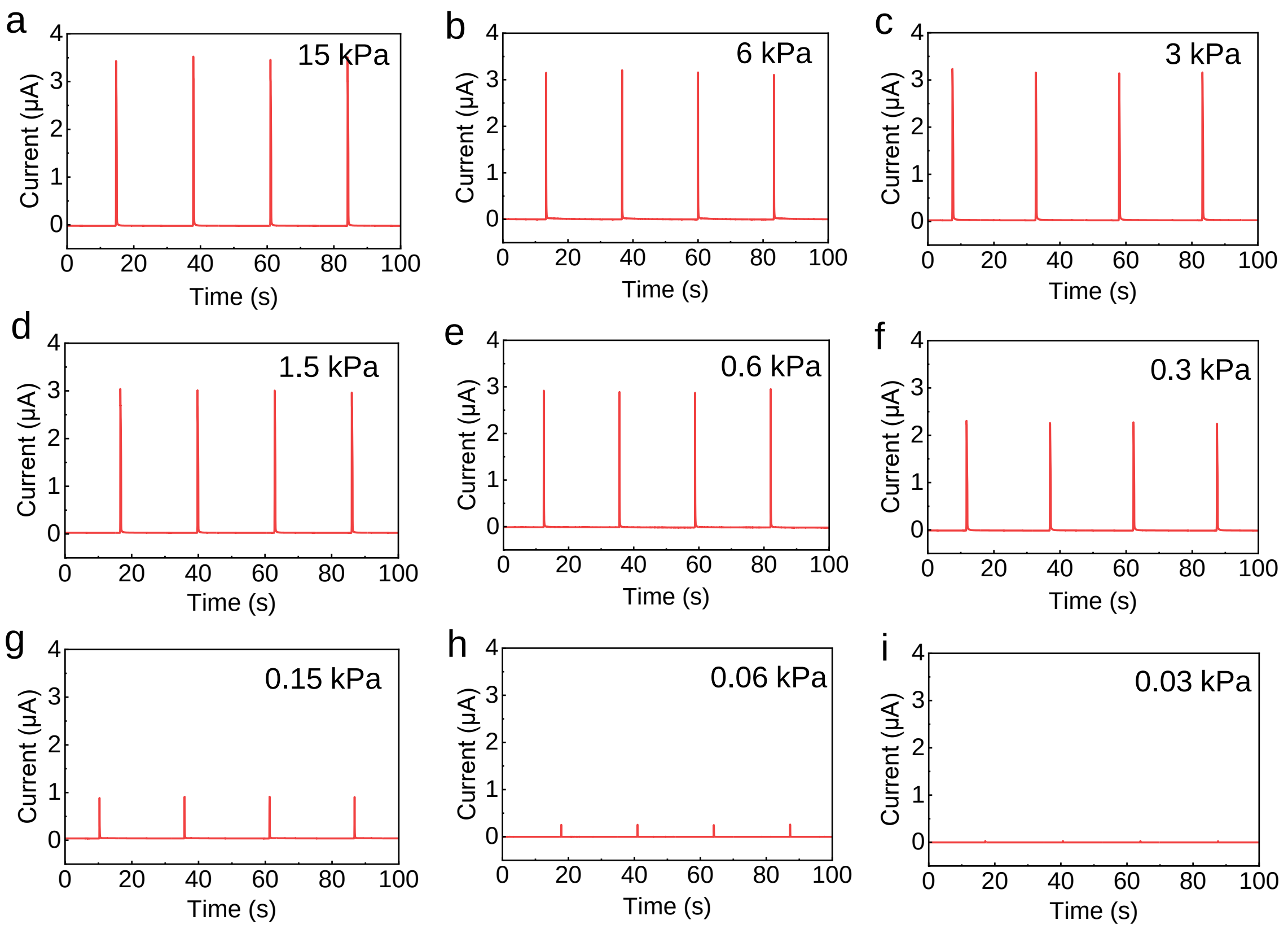

Supplementary Fig. 11 Piezoionic current responses under different pressures.

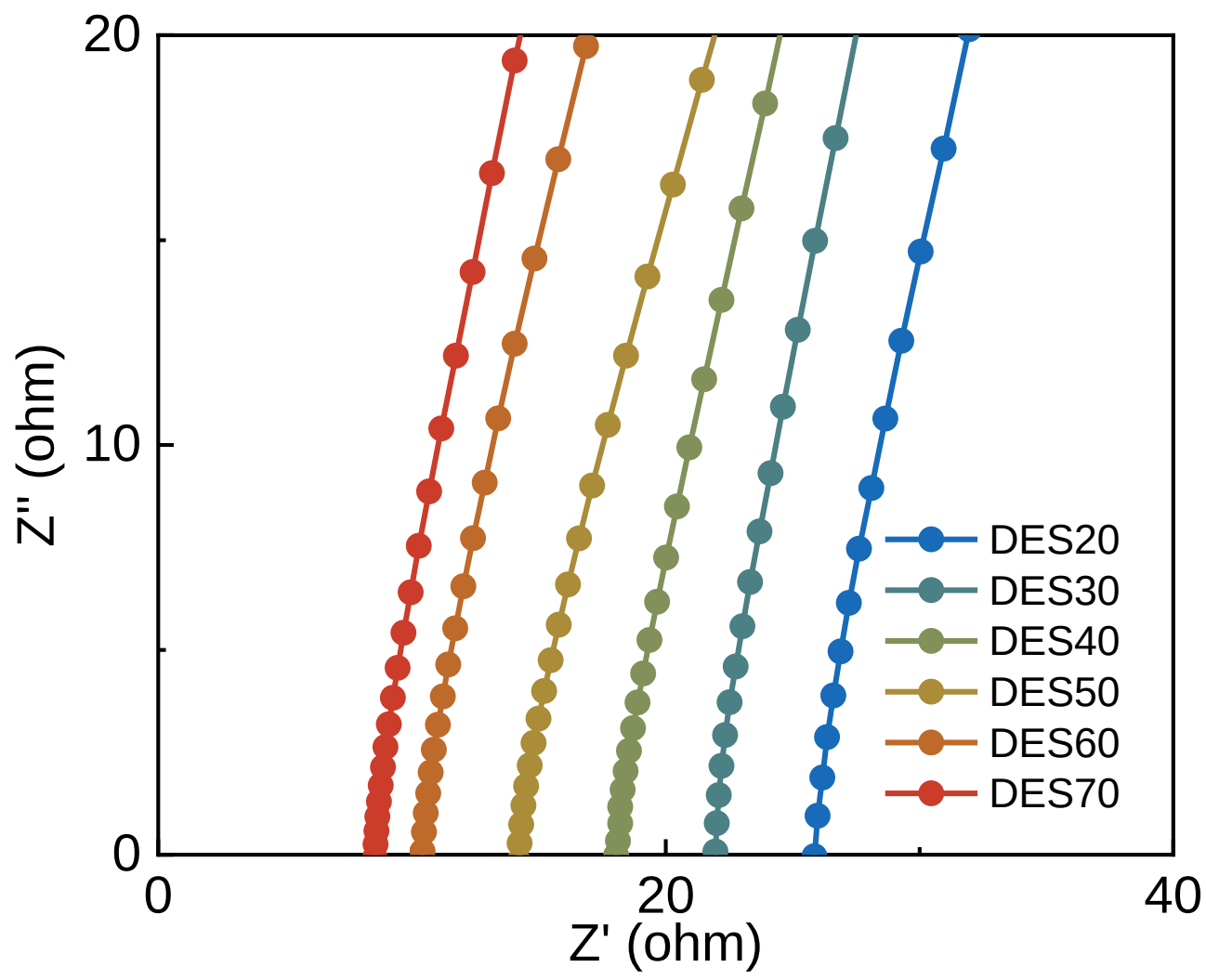

Supplementary Fig. 12 Electrochemical impedance spectra with different DES contents.

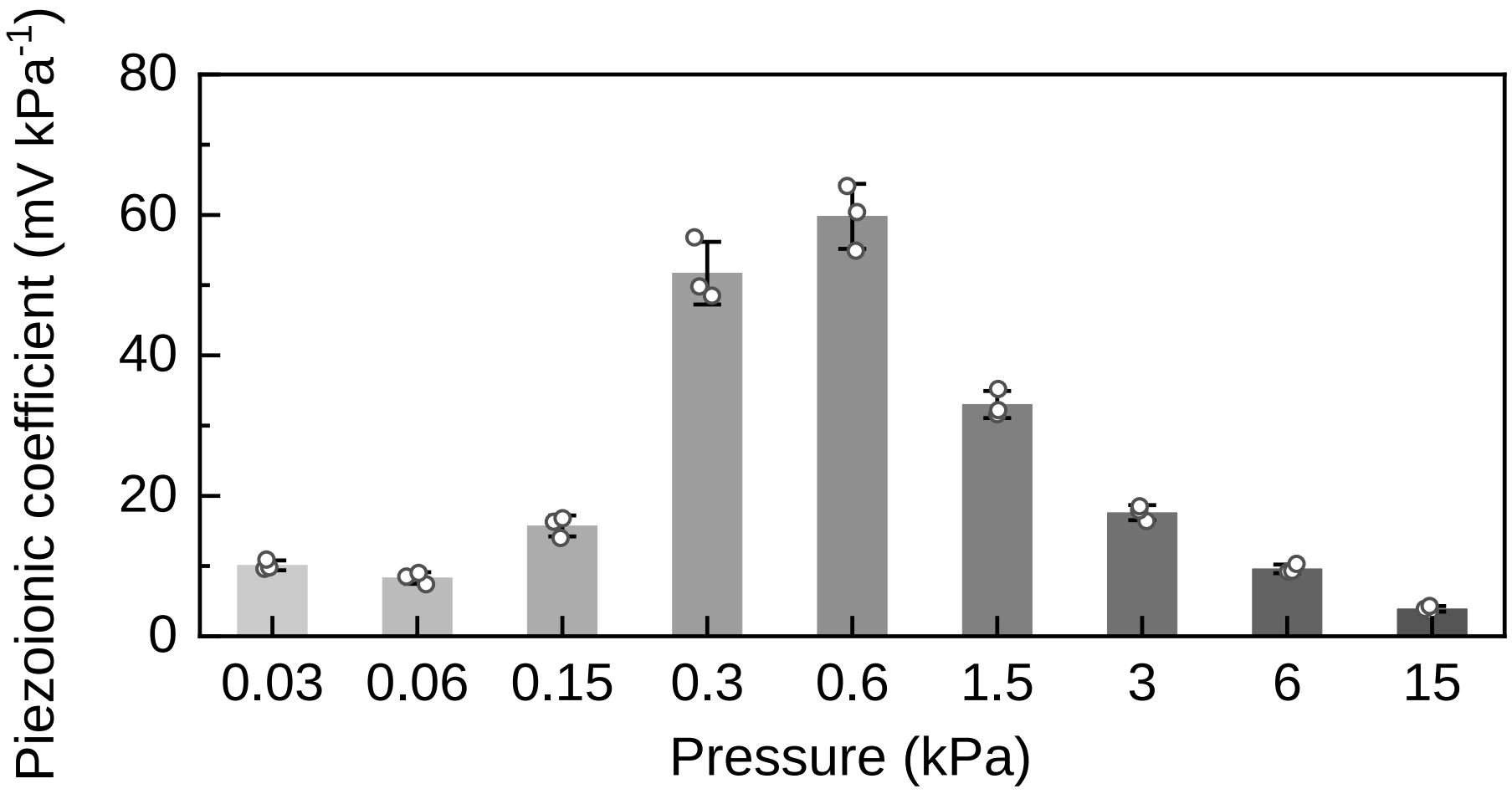

Supplementary Fig. 13 Piezoionic coefficients at different pressures. n = 3 independent samples. All statistical analyses were performed using one-way ANOVA, and Data are presented as mean value ± standard deviation (SD).

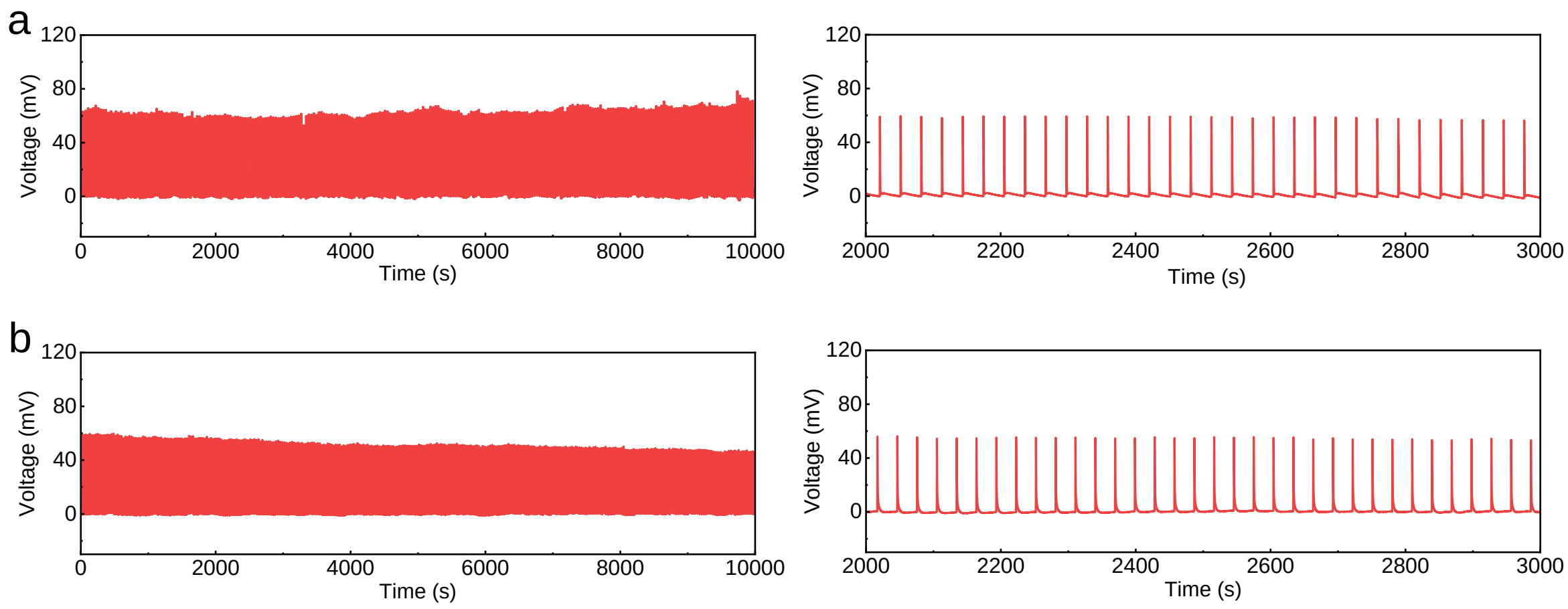

Supplementary Fig. 14 Long-term voltage stability and cyclic response (a) after 3 months and (b) after 6 months of storage, showing minimal performance and stability reduction.

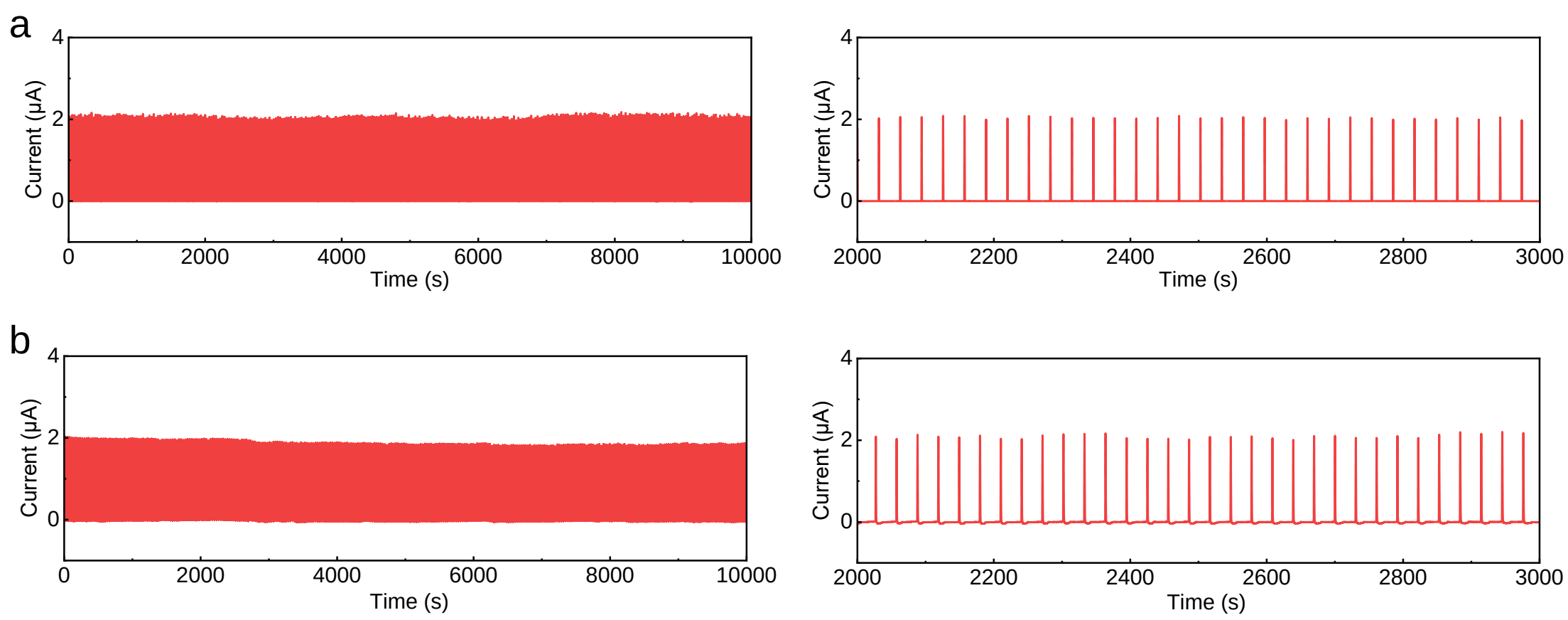

Supplementary Fig. 15 Long-term current stability and cyclic response (a) after 3 months and (b) after 6 months of storage, showing minimal performance and stability reduction.

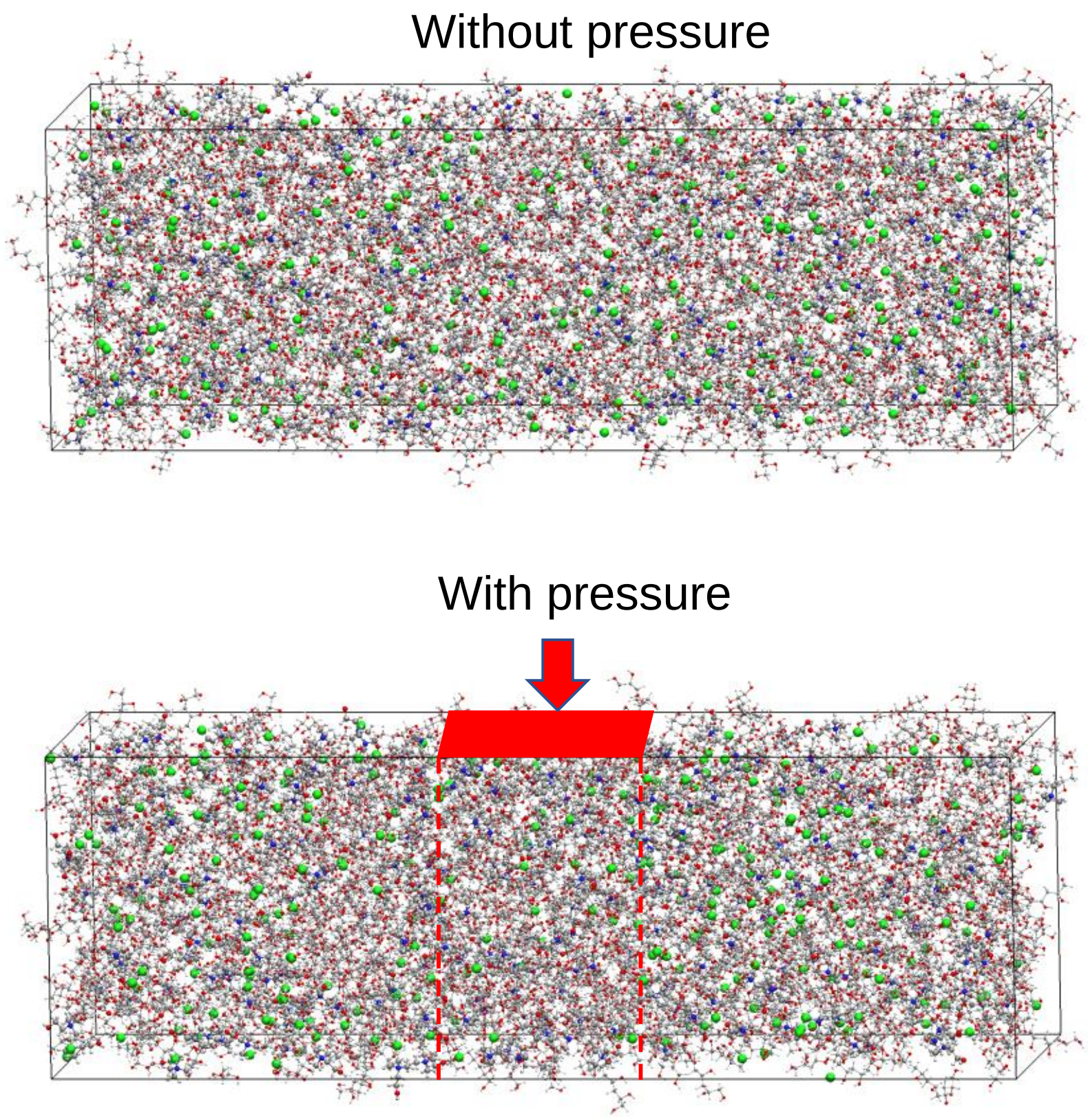

Supplementary Fig. 16 Models of the eutectogel under unstrained and compressed states.

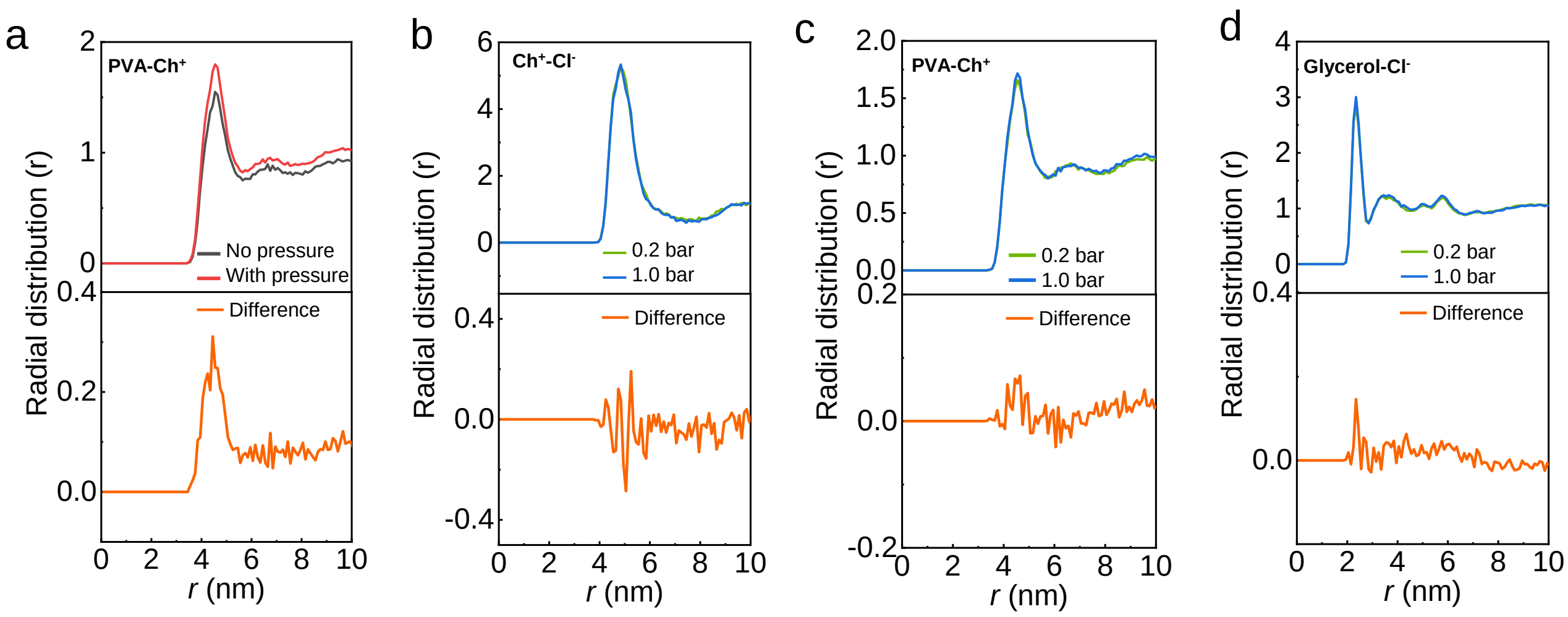

Supplementary Fig. 17 (a) RDF and calculated difference of PVA-Ch^{+} under unloaded and pressurized conditions. (b–d) RDF and calculated difference of Ch^{+}-Cl^{-}, PVA-Ch^{+} and glycerol-Cl^{-} pairs under the two relatively high pressures.

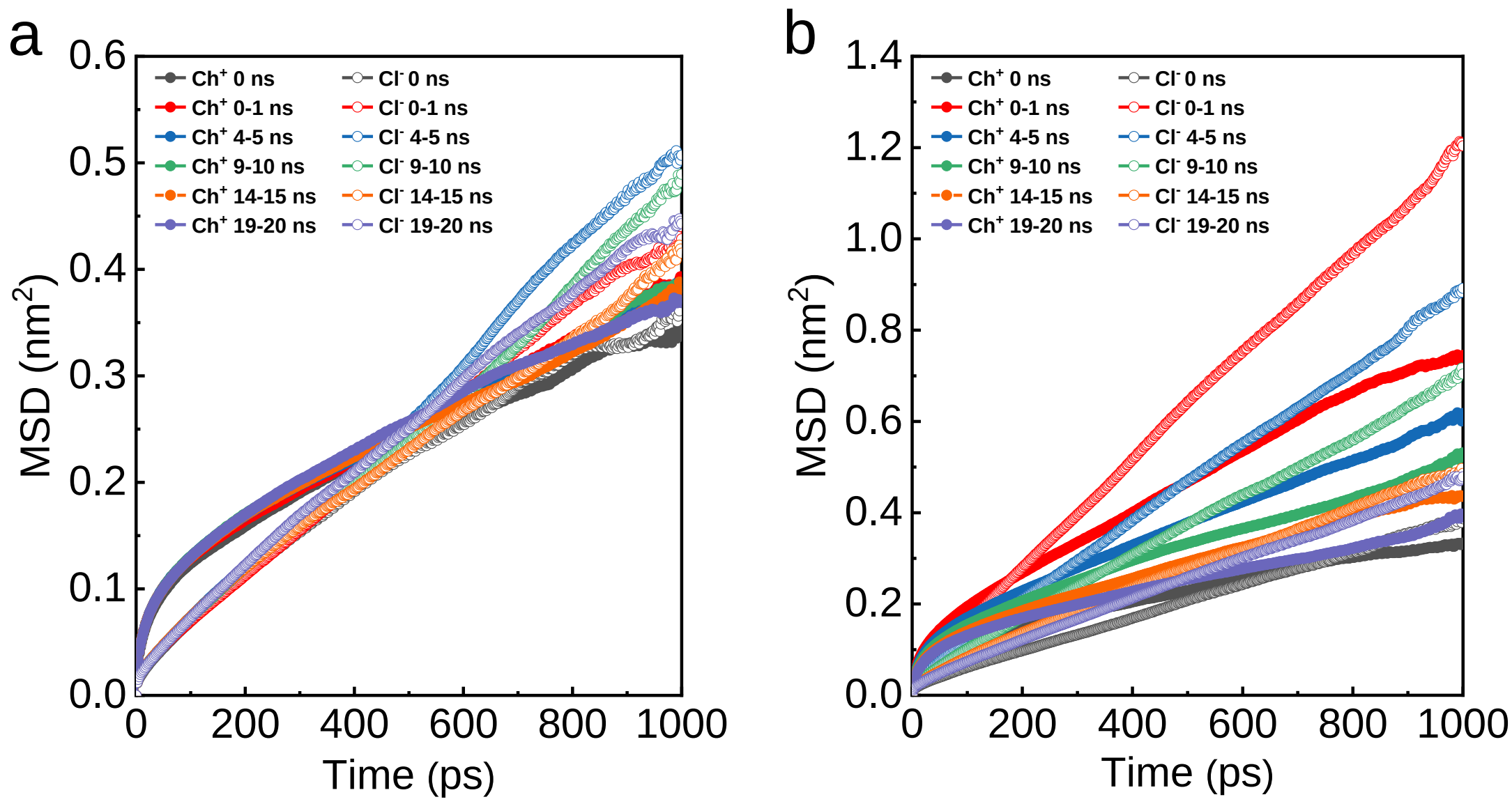

Supplementary Fig. 18 MSD of eutectogel under (a) without applied pressure and (b) with pressurized conditions.

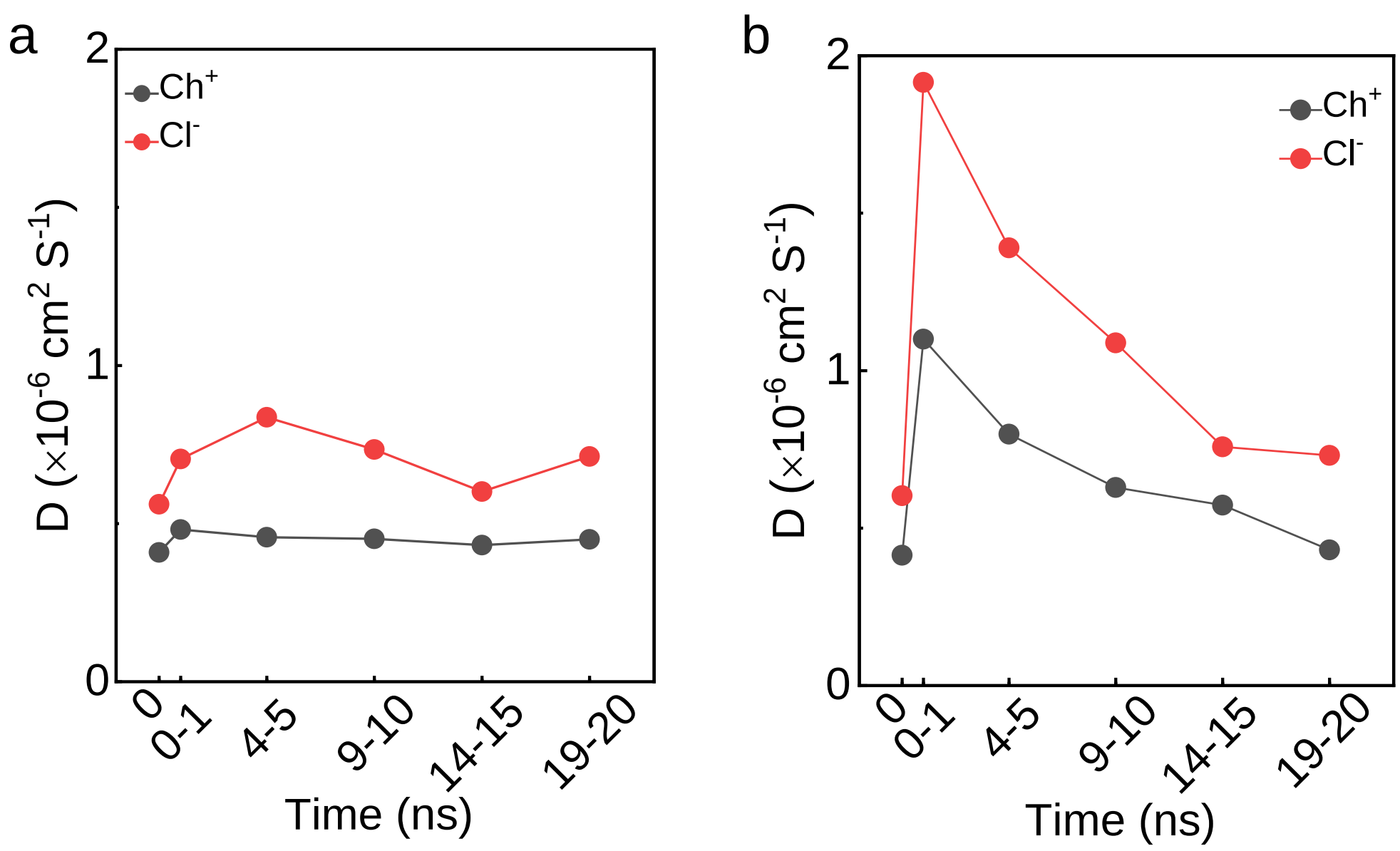

Supplementary Fig. 19 Calculated diffusivity of (a) without applied pressure and (b) with pressurized conditions.

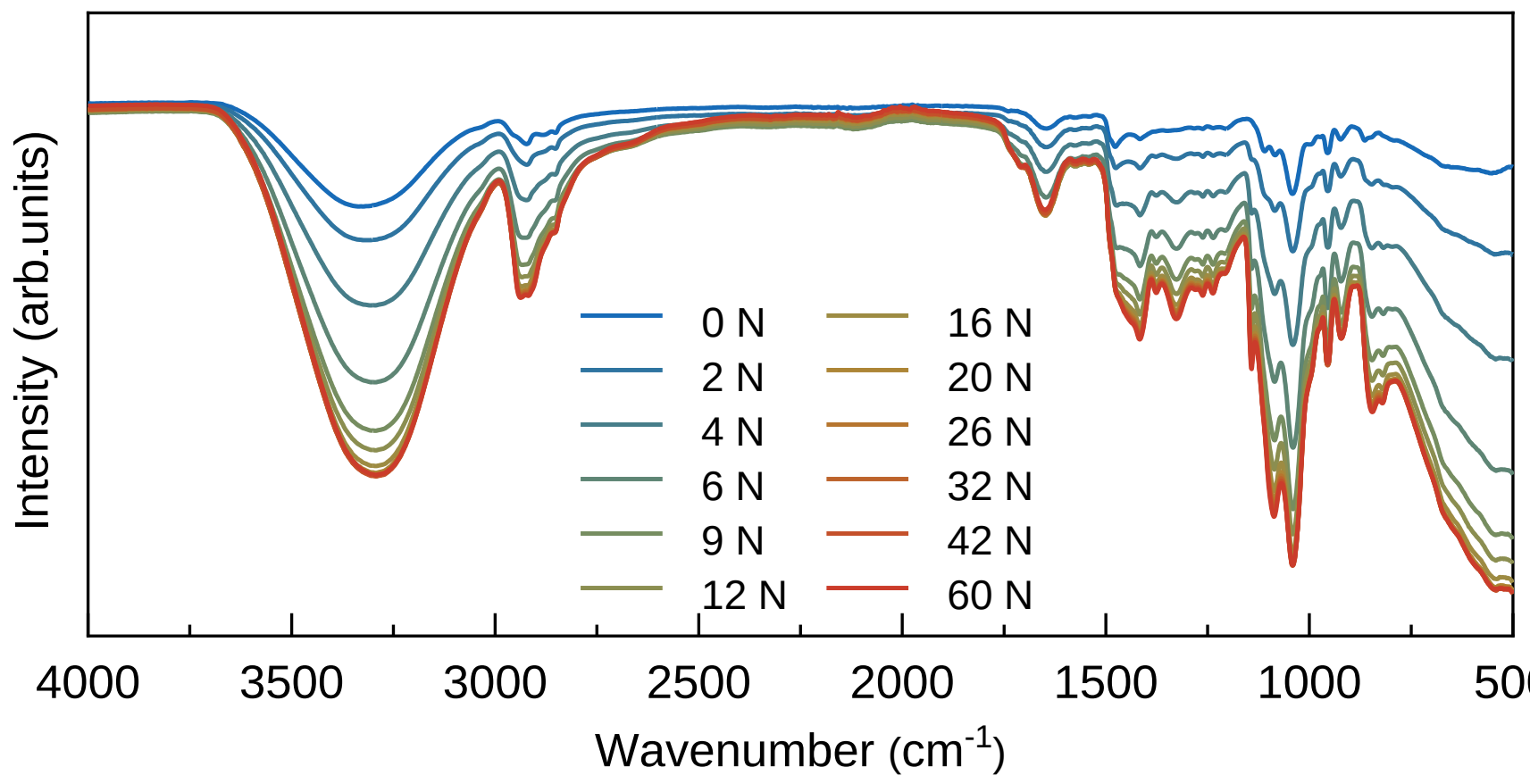

Supplementary Fig. 20 FTIR spectra of the eutectogel under different applied pressures.

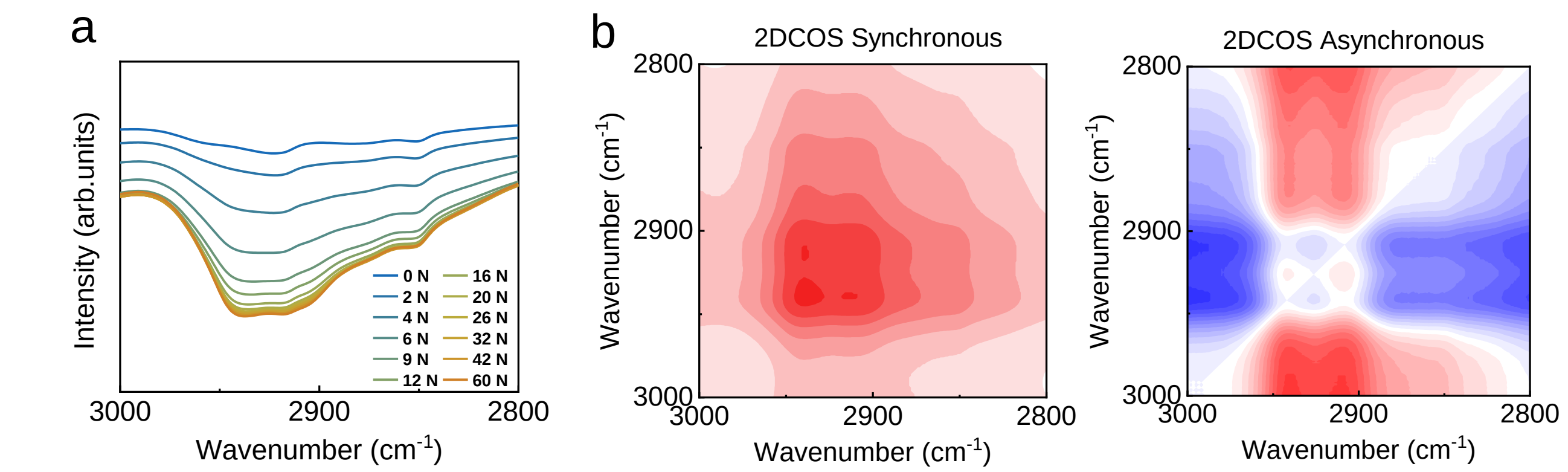

Supplementary Fig. 21 (a) FTIR spectra of the -CH groups under different applied pressures. (b) The corresponding 2D synchronous and asynchronous spectra.

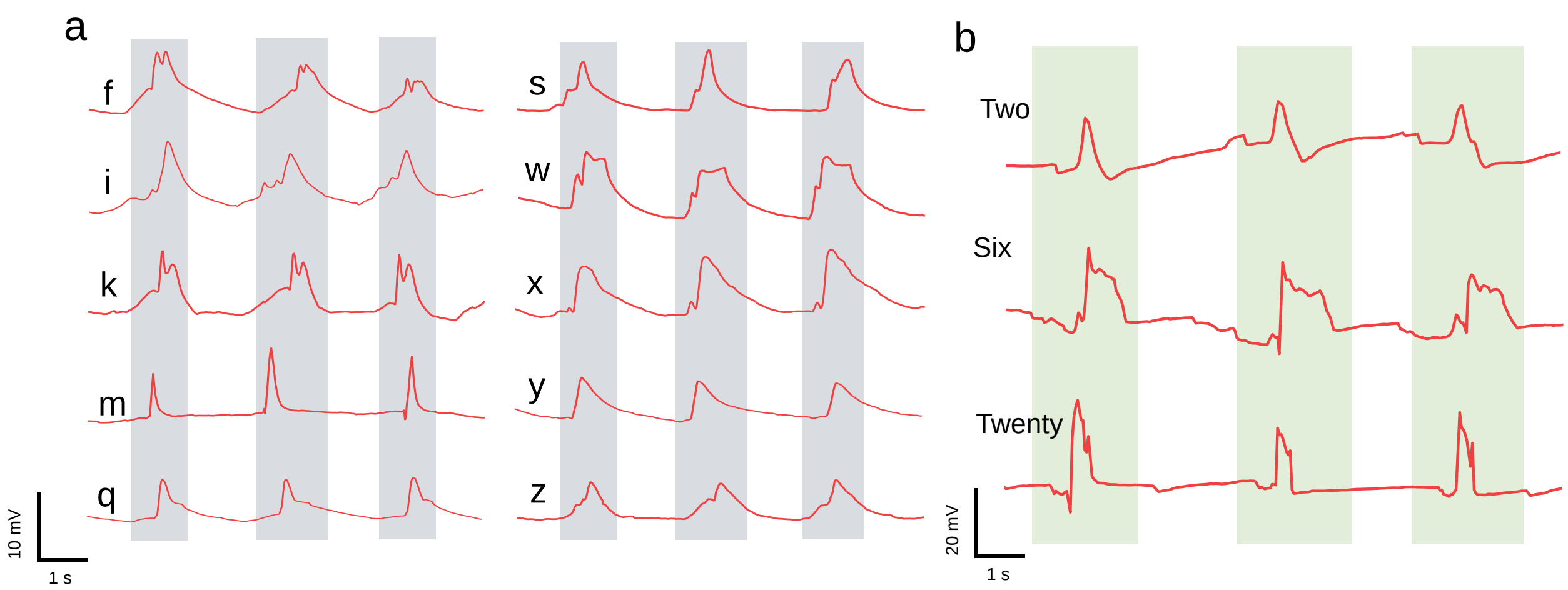

Supplementary Fig. 22 Throat vocalization recognition with different (a) English alphabet signature and (b) English number signature.

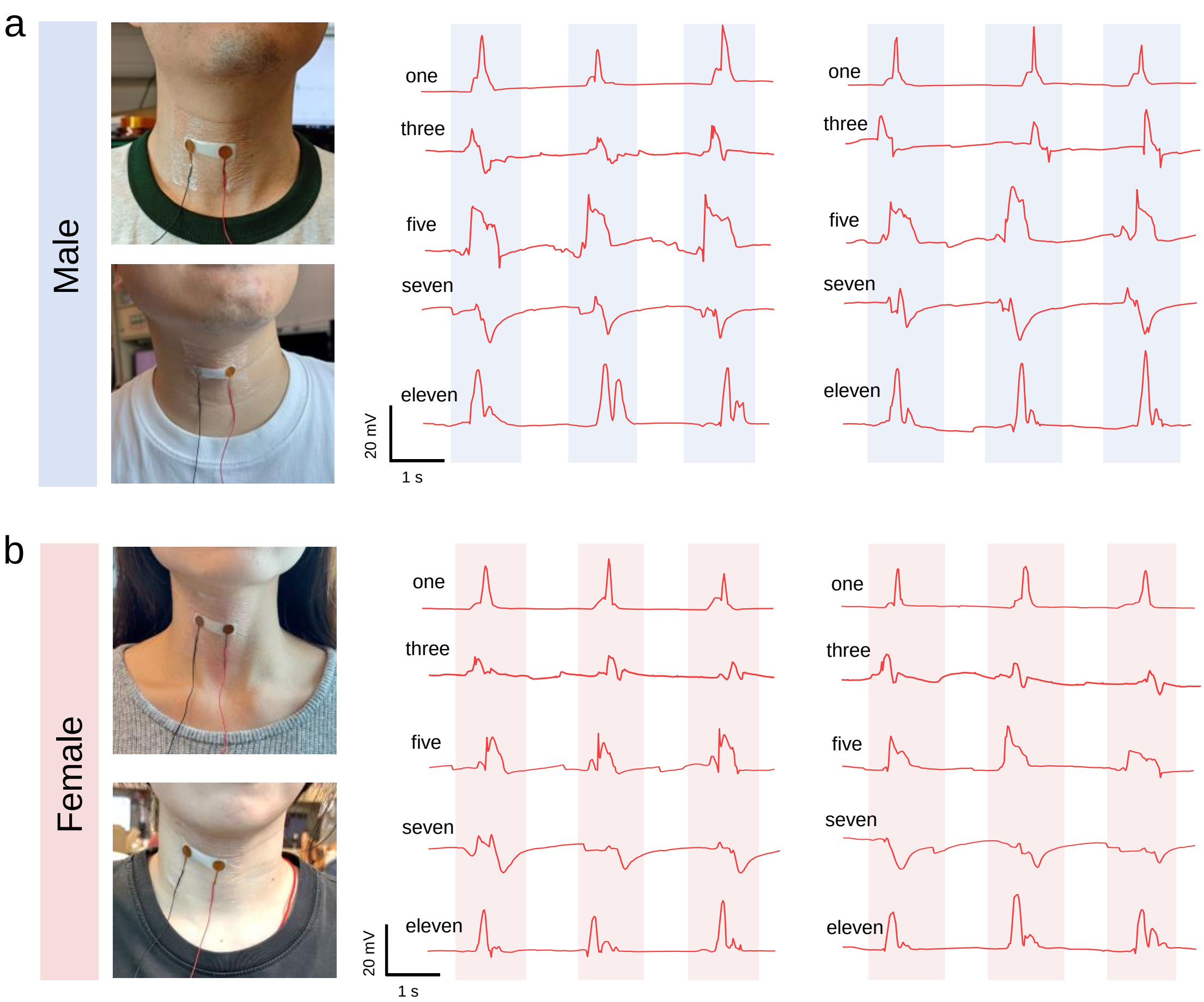

Supplementary Fig. 23 Piezoionic voltage signals generated by vocal cord vibrations during English word articulation captured by the eutectogel electrodes from (a) two male and (b) two female test participants.

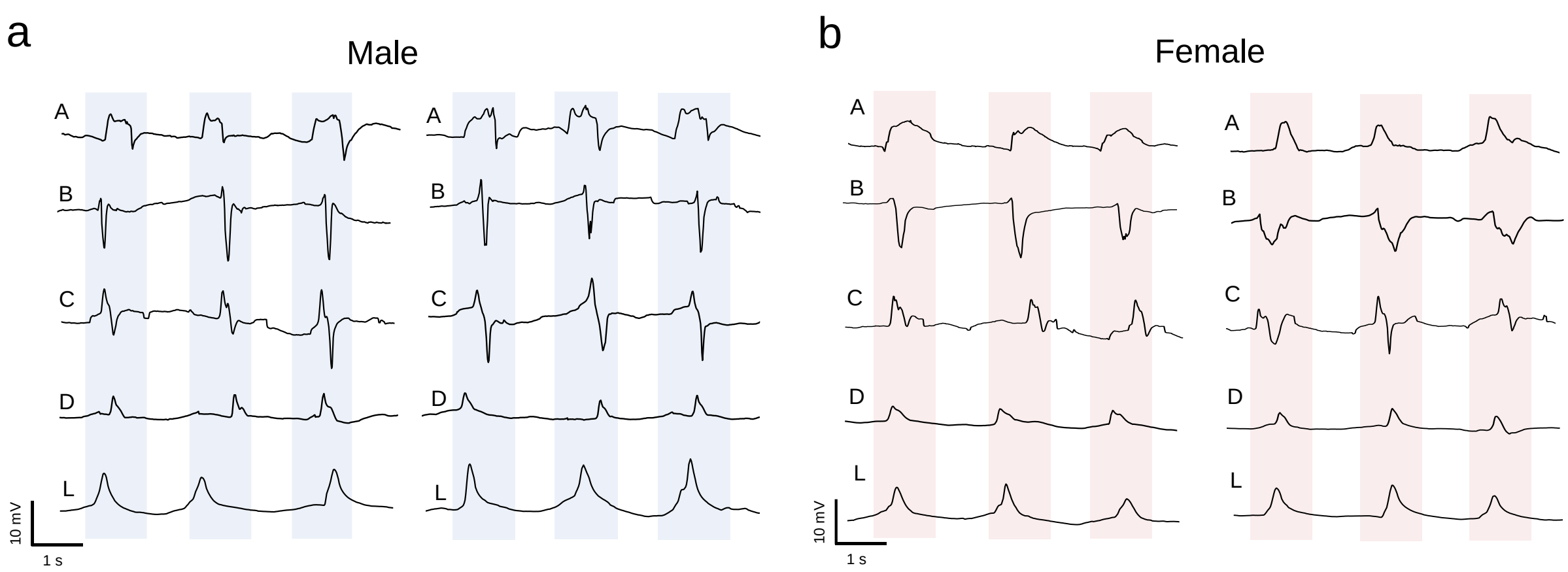

Supplementary Fig. 24 Piezoionic voltage signals generated during English alphabet articulation captured by the eutectogel electrodes from (a) two male and (b) two female participants.

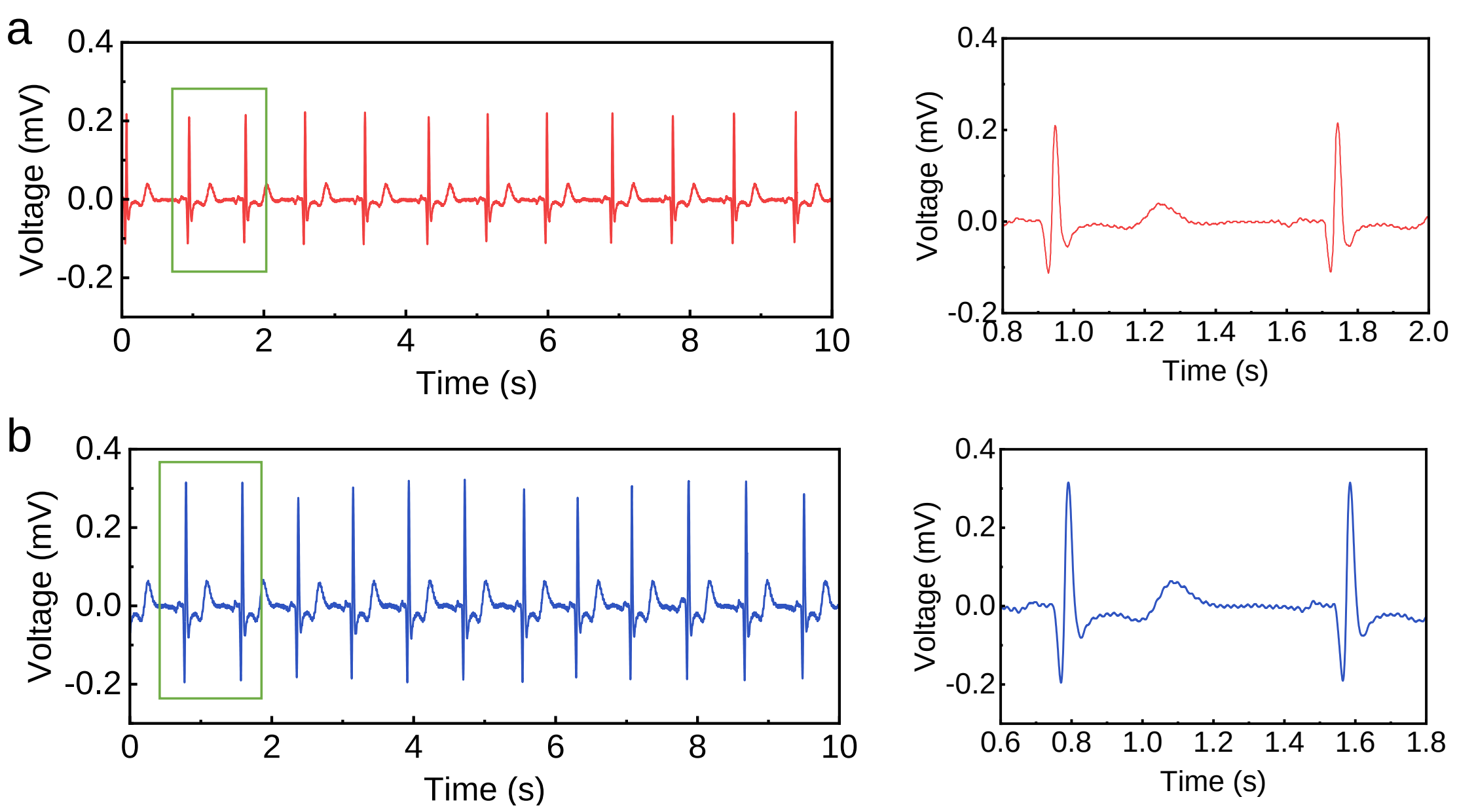

Supplementary Fig. 25 (a) ECG signal acquisition using eutectogels with DES50 as electrodes. (b) ECG signal acquisition using commercial electrodes, which is consistent with the data shown in Fig. 4i.

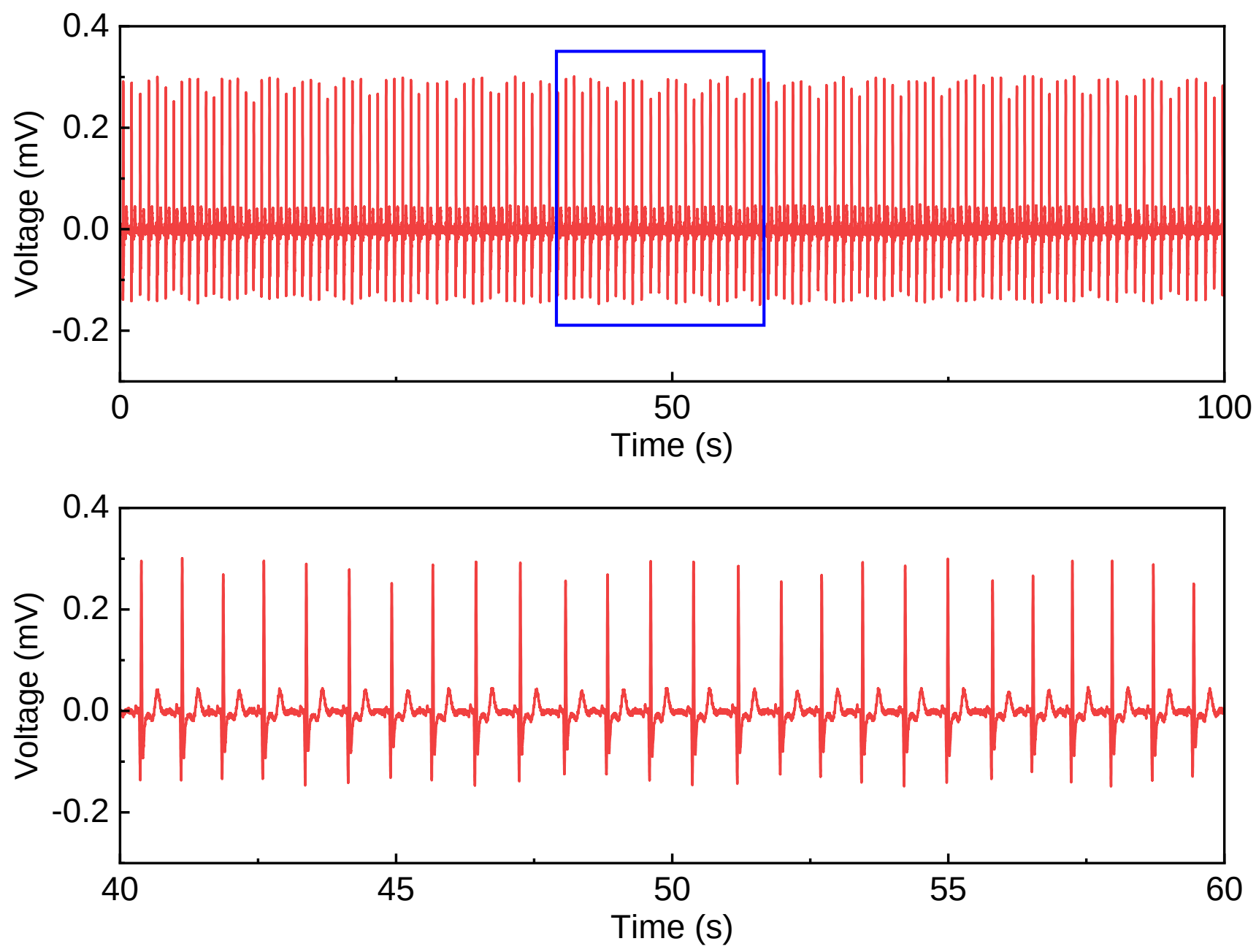

Supplementary Fig. 26 ECG signals recorded using DES70 after 6 months of storage.

Reference

1. Chen, S. et al. Facile Solvent regulation for highly strong and tough physical eutectogels with remarkable strain sensitivity. *ACS Appl. Mater. Interfaces* **15**, 44752–44762 (2023).
2. Wang, Y. et al. Deep eutectic solvent-induced microphase separation and entanglement of PVA chains for tough and reprocessable eutectogels for sensors. *Langmuir* **38**, 12189–12197 (2022).
3. Vo, TH. et al. One-step, additive-free fabrication of highly stretchable and ultra-tough physical polyvinyl alcohol-based eutectogels for strain sensors. *Chem. Eng. J.* **493**, 152877 (2024).
4. Du, S. et al. Ultratough and highly conductive supramolecular poly (Vinyl Alcohol) eutectogels via a sequentially enhanced strategy. *Adv. Funct. Mater.* **35**, 2409726 (2025).
5. Jiang, Y. et al. Mechanically robust eutectogels enabled by precisely engineered crystalline domains. *Nat. Commun.* **16**, 7417 (2025).
6. Akutsu, H. Direct determination by Raman scattering of the conformation of the choline group in phospholipid bilayers. *Biochemistry* **20**, 7359–7366 (1981).
7. Paschoal, VH. et al. Phase transitions of choline dihydrogen phosphate: A vibrational spectroscopy and periodic DFT study. *J. Chem. Phys.* **160**, 094507 (2024).
8. Murray, JS. et al. A revival of molecular surface electrostatic potential statistical quantities: ionic solids and liquids. *Crystals* **14**, 2073–4352 (2024).
9. Shi, Z. et al. Coordination-escorted organo-interhalogen conversion enables durable dual-deposition Zn||I_2 batteries with high areal capacities. *Energ. Environ. Sci.* **19**, 561–573 (2026).
10. Lu, T. et al. Multiwfn: A multifunctional wavefunction analyzer. *J. Comput. Chem.* **33**, 580–592 (2012).
11. Schwerdtfeger, P. et al. 100 years of the Lennard-Jones potential. *J. Chem. Theory Comput.* **20**, 3379–3405 (2024).
12. Martínez, L. et al. PACKMOL: A package for building initial configurations for molecular dynamics simulations. *J. Comput. Chem.* **30**, 2157–2164 (2009).
13. Cao, Z. *et al.* Multifunctional eutectic hydrogel with antifreezing and self-powered capabilities for object recognition sensing. *Adv. Funct. Mater.* **36**, e2904 (2025).
14. Cai, C. *et al.* Harnessing the hofmeister effect for simultaneous strengthening and toughening of cellulosic triboelectric materials. *Angew. Chem. Int. Ed. Engl.* **65**, e21123 (2026).
15. Shi, X. *et al*. Mapping the structural evolution in supercooled polysiloxane liquids: a combined temperature-resolved WAXD and 2D-COS study. *J. Mater. Chem. C*, **14**, 2847–2853 (2026).
16. Wang, X. *et al.* Identification of camellia oil adulteration by using near infrared spectroscopy combined with two dimensional correlation spectroscopy (2DCOS) analysis. *Infrared Phys. Techn.* **149**, 105902 (2025).
17. Muratova, M. *et al.* Application of two-dimensional correlation spectroscopy to chemical

analysis. *J. Anal. Chem.* **79**, 339–365 (2024).
18. Park, Y. *et al*. Novel developments and progress in two-dimensional correlation spectroscopy (2D-COS). *Appl. Spectrosc.* **79**, 13–35 (2025).
