# Supplementary material for: Biocompatible piezoionic eutectogel sensor for self-powered physiological monitoring
Source: Nat Commun. 2026 Sep 23;17:11053. doi: 10.1038/s41467-026-77390-7 (PMC13691130; doi:10.1038/s41467-026-77390-7)
Supplement: Supplementary file 3 [file 41467_2026_77390_MOESM3_ESM.pdf]

nature portfolio

Peer Review File

Biocompatible Piezoionic Eutectogel Sensor for Self-Powered Physiological Monitoring

Corresponding Author: Professor Derek Ho

Parts of this Peer Review File have been redacted as indicated to maintain the confidentiality of unpublished data.

This file contains all reviewer reports in order by version, followed by all author rebuttals in order by version.

Version 0:

Reviewer comments:

Reviewer #1

(Remarks to the Author)
This manuscript reports a biocompatible piezoionic eutectogel biosensor based on a choline chloride–glycerol deep eutectic solvent incorporated into a PVA network. By engineering hydrogen-bond interactions to selectively immobilize cations while maintaining high anion mobility, the authors achieve enhanced piezoionic output, good mechanical compliance, and excellent biocompatibility. The work further demonstrates self-powered epidermal sensing of pressure and bioelectrical signals, including arterial pulse waves, vocalization, and ECG signals.

Overall, the study is technically rigorous, well-supported by multiscale characterization (DFT, MD, spectroscopy), and addresses an important challenge in piezoionic and ionotronic bioelectronics: simultaneously achieving high output, environmental stability, and biosafety. The concept of using DES-based eutectogels for piezoionic sensing is timely and, to the best of my knowledge, novel in the context of physiological diagnostics.

However, the manuscript in its current form is overly long, repetitive, and at times difficult to follow, particularly in the Results and Discussion. Several claims should be sharpened or moderated, and the narrative structure could be significantly improved to meet the standards of a high-impact journal.

1. The results and discussion starts with a Figure and the initial paragraph is somehow confusing. This should be improved (transition from introduction to results).
2. Line 155. "DES70" it is not clear what this is. Please clarify. A table with the DES composition MUST be included.
3. Results from eutectogel characterization and biocompatibility should be subchapters since they are two completely different subjects.
4. no sup information file was available.
5. Figure 2 has too many images. It makes the reading very difficult. Figures must be separated and introduced everytime they are mentioned to make the reading easier.
6. cytotoxicity assay (methodology). needs further details such as cell density.

I believe that at this stage the manuscript does not show the level of quality to be submitted in Nature Communications.

Reviewer #2

(Remarks to the Author)
This paper fabricated a piezoionic hydrogel by using hydrogen bonds to selectively immobilize cations and enable rapid transport of anions. The piezoelectric effect was explained through DFT simulations and FTIR. However, the proposed gel and exhibited piezoionic mechanism seem not novel enough. Some declamations and interpretations did not provide reasonable discussions. In addition, the demonstrated physiological monitoring did not show new points. As a result, this paper should be rejected.
1. In the Introduction, the authors argue that conventional piezoelectric materials suffer from limitations such as the lack of seamless integration, signal stability, and long-term reliability. However, the present study does not convincingly

demonstrate that these challenges have been resolved. In particular, long-term signal stability, which is central to the authors' motivation, is neither rigorously evaluated nor substantiated by sufficient experimental evidence.
2. Figure 2c indicates pronounced creep behavior during the first tensile cycle, suggesting substantial viscoelastic relaxation. In contrast, Figure 2m reports highly stable piezoionic signals under cyclic loading. The manuscript does not reconcile this apparent inconsistency. Without a mechanistic explanation or additional controls, the stability of the electrical response remains questionable.
3. Figure 3j is incorrectly cited as Figure 4j. More critically, the selection of the four characteristic wavenumbers (2998 cm^{-1}, 3278 cm^{-1}, 3291 cm^{-1}, and 3544 cm^{-1}) used in the 2D-COS analysis lacks justification. These spectral features are not clearly identifiable in Figure 3j, and the 2998 cm^{-1} band appears outside the displayed spectral window. The authors must clarify how these bands were determined and why they were chosen.
4. The photograph in Figure 4c suggests that the strap used for encapsulation and assembly provides negligible back pressure. Given that the proposed piezoionic generation mechanism fundamentally relies on mechanically induced deformation, the experimental configuration appears insufficient to generate meaningful gel strain. The manuscript fails to adequately explain how effective deformation was achieved.
5. The pulse waveforms presented in Figure 4g do not exhibit recognizable tidal wave characteristics, which are typically expected in physiological pulse signals. Additionally, the authors' claim that "This is the first demonstration of a piezoionic eutectogel ever reaching such high level of precision" is not supported by quantitative analysis or benchmarking.
6. The realization of ECG acquisition in Figure 5i is insufficiently explained. According to the mechanical characterization (Figures 2a–c), the gel exhibits a Young's modulus significantly higher than that of human skin, implying poor conformability and suboptimal electrode-skin coupling. The authors do not address how reliable ECG signals were obtained despite this apparent mechanical incompatibility.
7. As we know, the ionic piezoelectric effects in gels usually occurs due to the relatively rapid movement of cations compared with anions. The cations have smaller sizes which the anions have larger sizes. However, in the manuscript, the authors proposed a different model.
8. In Fig. 2i, the gel shows a larger resistance (~9 kΩ). It is known to all that low internal resistance of gels can facilitate the ECG measurement. Why can this gel be used in ECG experiments? How did the authors think about this?
9. ECG tests do not require power supply at all. They merely collect the signals of the heart's beats. How did the authors interpret the concept of self-powered?
10. In Fig. 3g, the difference between two states seems not distinct. Could the simulations support the declaimed result?
11. In Fig. 3e, how did the authors obtain the result between unloading and pressurized states?

Reviewer #3

(Remarks to the Author)
in this study titled "Biocompatible Piezoionic Eutectogel Biosensor for Soft Self-Powered Human Physiological Diagnostics", the authors performed some attractive and valuable research. The developed eutectogels were characterized and applied successfully as Piezoionic biosensors. Few comments for further improvement are here;

1. The current advanced bioelectonic devices require a high adhesive strength, self-healing behavior, etc., the authors performed no such study.
2. The vocal cord vibrational sensitivity was elaborated towards different alphabets. It will be better to check the same words articulated by different people. To confirm the selectivity of the sensor towards vibration patterns of the vocal cords exactly.
3. In supplementary Fig. 5, the notations (a, b, c,..g) are not described.
4. The authors stressed in introduction that before this study no Eutectogels are used for sensing purpose. However, such sentences should be revised. Because the literature is available on Eutectogels used for strain sensing applications.

Version 1:

Reviewer comments:

Reviewer #1

(Remarks to the Author)
The authors submitted a revised version of their manuscript. This version is substantially improved and the authors have made a genuine effort to respond to reviewer comments. The added long-term stability data, the clarification of tensile versus compressive behavior, and the more detailed mechanistic interpretation strengthen the paper considerably. That said, the manuscript still feels somewhat overpacked, and some claims remain more ambitious than the evidence fully supports. Despite the effort I still have some concerns, namely:
1. The manuscript would benefit from further tightening, especially in the Results and Discussion, where the narrative remains dense and occasionally repetitive. The scientific content is strong, but the presentation still reads as a heavily annotated revision rather than a fully streamlined final article.
2. Several claims should be moderated slightly so they match the data more precisely, particularly claims about novelty and the extent to which long-term stability and device integration have been "solved." The data supports a strong proof-of-concept, but not a universal resolution of the field's limitations.
3. A few figure-related issues should be checked one more time before acceptance, including consistency of labels, nomenclature, and cross-references between the main text and Supplementary Information. These are minor on their own, but in aggregate they affect readability and confidence.
4. Improve sentence-level language for clarity and concision in several sections, especially where the revision currently repeats the rebuttal logic inside the manuscript.

5. Ensure all descriptors for DES20, DES50, DES70, etc. are defined clearly at first use and used consistently throughout the manuscript and captions.
6. Consider making the abstract slightly shorter and more direct, emphasizing the central message: a biocompatible DES-PVA eutectogel with strong piezoionic response and practical wearable sensing performance.

Therefore, I believe that these minor details would greatly improve the quality of the manuscript.

Reviewer #2

(Remarks to the Author)
Although the authors have revised the manuscript to some extent, the following issues remain unresolved. Hence, i do not think this paper can reach Nat. Commun.'s scientific level, and should be transferred to other journals.
1.In Fig. 1, the schematic representation of the "Piezoionic potential" is incorrect. The authors should treat this with greater rigor.
2.In Fig. 3j, please explicitly list relevant references for the chemical bonds indicated at different wavelength positions, particularly for "PVA-Ch+" and "glycerol-Cl^-".
3.The authors state, "During speech or swallowing, vocal cord vibration induces localized deformation of the overlying skin, generating compressive stresses on the order of 0.5–1.5 kPa." It seems unlikely that such high pressure can be achieved without back pressure. How was this value determined? Furthermore, the PU membrane mentioned by the authors appears to vibrate synchronously with the sensor and does not seem to provide a supporting function.
4.The authors did not conduct any experiments for DES content greater than 70%. How was it determined that DES70 is the optimal concentration? Moreover, the authors did not use DES70 in the majority of experiments throughout the manuscript, including "biocompatibility, voltage and current under cyclic compression." Why was DES70 switched to only for the application demonstration?
5.In Fig. 2n, when performing the comprehensive performance comparison, the authors combined the best advantages of samples with different DES concentrations. This does not represent a single sample. For instance, how were the values for "maximum voltage 80, ionic conductivity 3, Young's modulus 24" achieved simultaneously?

Reviewer #3

(Remarks to the Author)
The manuscript has been revised appropriately and now can be accepted.

Open Access This Peer Review File is licensed under a Creative Commons Attribution 4.0 International License, which permits use, sharing, adaptation, distribution and reproduction in any medium or format, as long as you give appropriate credit to the original author(s) and the source, provide a link to the Creative Commons license, and indicate if changes were made.
In cases where reviewers are anonymous, credit should be given to 'Anonymous Referee' and the source.
The images or other third party material in this Peer Review File are included in the article’s Creative Commons license, unless indicated otherwise in a credit line to the material. If material is not included in the article’s Creative Commons

license and your intended use is not permitted by statutory regulation or exceeds the permitted use, you will need to obtain permission directly from the copyright holder.
To view a copy of this license, visit https://creativecommons.org/licenses/by/4.0/

Point-by-Point responses to Reviewers' Comments

We sincerely thank the reviewers for their careful and thorough review, which are indeed very helpful to make the paper more solid and smooth. We have revised our manuscript very carefully in light of their suggestions and comments.

The following responses have been prepared to address all of the reviewers' comments in a point-by-point fashion. (Comments in black, response in blue, and revisions highlighted in yellow):

Response to Reviewer #1

General comment: This manuscript reports a biocompatible piezoionic eutectogel biosensor based on a choline chloride-glycerol deep eutectic solvent incorporated into a PVA network. By engineering hydrogen-bond interactions to selectively immobilize cations while maintaining high anion mobility, the authors achieve enhanced piezoionic output, good mechanical compliance, and excellent biocompatibility. The work further demonstrates self-powered epidermal sensing of pressure and bioelectrical signals, including arterial pulse waves, vocalization, and ECG signals.

Overall, the study is technically rigorous, well-supported by multiscale characterization (DFT, MD, spectroscopy), and addresses an important challenge in piezoionic and ionotronic bioelectronics: simultaneously achieving high output, environmental stability, and biosafety. The concept of using DES-based eutectogels for piezoionic sensing is timely and, to the best of my knowledge, novel in the context of physiological diagnostics. However, the manuscript in its current form is overly long, repetitive, and at times difficult to follow, particularly in the Results and Discussion. Several claims should be sharpened or moderated, and the narrative structure could be significantly improved to meet the standards of a high-impact journal.

Response: We sincerely thank the reviewer for the encouraging and insightful evaluation of our work. We are particularly encouraged by the recognition of our study as "technically rigorous" and "well-supported by multiscale characterization." We also appreciate the acknowledgement of our efforts to address key challenges related to output performance, stability, and biosafety in piezoionic bioelectronics, as well as the novelty of employing eutectogels for physiological signal monitoring.

We have carefully considered the constructive comments regarding the presentation of the manuscript. In response to concerns about length, redundancy, and narrative flow, we have undertaken a comprehensive revision of the manuscript. Specifically, the Results and Discussion section has been streamlined to reduce repetition and emphasize the key mechanisms. The overall structure has been reorganized to improve logical flow and clarity, and all claims have been carefully refined to ensure they are fully supported by the experimental data. We believe that these revisions have significantly improved the clarity and rigor of the manuscript and have addressed your concerns.

1. The results and discussion start with a Figure and the initial paragraph is somehow confusing. This should be improved (transition from introduction to results).

Response: Thank you very much for your valuable suggestion. We concur that introducing Fig. 1 without an immediate textual lead-in may disrupt the narrative flow and lead to potential confusion. Accordingly, Fig. 1 has been repositioned to a more appropriate location that aligns with the corresponding experimental discussion. In addition, the first section title in the Results and Discussion has been revised to "Molecular and structural design of DES-PVA eutectogels," ensuring a smoother transition from the introductory paragraph to Fig. 1 and improving overall logical consistency.

Furthermore, we have carefully reassessed the role of the initial paragraph. In line with common practices in high-impact studies (e.g., ***Nat. Commun.*** 2025, 16, 8129; ***Nat. Commun*.** 2025, 16, 11156; ***Sci. Adv.*** 2025, 11, eadx2628; ***Adv. Mater.*** 2024, 36, 2309821), this paragraph is intended to establish the core design rationale and conceptual framework prior to the presentation of characterization results. By clearly outlining the design strategy and underlying mechanisms at the outset, it provides a logical foundation that facilitates the interpretation of subsequent experimental data and theoretical analyses (DFT and MD). Therefore, this paragraph has been retained to ensure a coherent transition from the Introduction to the Results and Discussion section and to support a clear understanding of the subsequent mechanistic and performance analyses. We believe that this revised structure improves the clarity and readability of the manuscript.

Our revision to the manuscript:

The first section title has been revised to "Molecular and structural design of DES-PVA

eutectogels”. (**Section**: Results and Discussion, **Paragraph**: first title)

2. Line 155. "DES70" it is not clear what this is. Please clarify. A table with the DES composition MUST be included.

Response: Thank you for pointing out this potential confusion. In our manuscript, "DES70" refers to the eutectogel formulated with 70 wt% deep eutectic solvents (DES), and this nomenclature is consistently applied across all samples, where the numerical suffix denotes the DES weight fraction. In the original version, this definition was provided only in the caption of Fig. 1a, which may not have been sufficiently prominent. To improve clarity, we have now explicitly introduced this definition in the main text at its first occurrence. In addition, a comprehensive summary of the compositions of all eutectogel samples has been provided as Supplementary Table 1 to ensure transparency and facilitate comparison.

Supplementary Table R1. DES composition of the prepared eutectogels

**Name**	PVA film	DES20	DES30	DES40	DES50	DES60	DES70
DES (wt%)	[illegible]	[illegible]	[illegible]	[illegible]	[illegible]	[illegible]	[illegible]
PVA (wt%)	100	[illegible]	[illegible]	[illegible]	[illegible]	[illegible]	[illegible]

Our revision to the manuscript:

We have included the definition in the revised manuscript and added Table R1 as Supplementary Table 1 in Supplementary Information.

“Eutectogels obtained after thermal treatment likewise exhibit phase separation, which is manifested as a translucent white appearance when the DES content exceeds 20 wt% (Fig. 1b). Specifically, “DES20” and “DES70” denote the eutectogels containing with 20 wt% and 70 wt% DES (Supplementary Table 1), respectively.” (**Section**: Results and Discussion, **Paragraph**: 2)

3. Results from eutectogel characterization and biocompatibility should be subchapters since they are two completely different subjects.

Response: Thank you for the professional suggestion. We agree that physical characterization and biocompatibility are typically presented as independent subchapters in most reported studies. In our original manuscript, these two aspects were

already discussed in separate paragraphs. Nevertheless, we would like to clarify the rationale for integrating them within Fig. 1.

In our research framework, cell viability is considered a fundamental indicator of the biological performance of the eutectogels. As the materials are designed for epidermal sensing applications, biocompatibility is regarded as an essential material property alongside physicochemical characteristics. Therefore, establishing the biological safety profile at an early stage provides a comprehensive foundation before subsequent device-level demonstrations. We also considered relocating the cytotoxicity results to later sections (e.g., alongside Figs. 2–4). However, these sections are primarily dedicated to piezoionic sensing mechanisms and performance. Introducing biocompatibility data at that stage would disrupt the logical progression of the manuscript. In this context, the cytotoxicity assay serves as a key component of material characterization, complementing the physicochemical analysis presented in Fig. 1. Accordingly, Fig. 1 is designed to provide an integrated overview of the fundamental properties of the eutectogels, including both structural and biological characteristics, which we consider to be the most logical organization of these results.

Following your suggestion, we have further improved the presentation by explicitly separating the cytotoxicity analysis into an independent paragraph. This refinement enhances thematic clarity while preserving the overall logical coherence of the manuscript.

Our revision to the manuscript:

"Biocompatibility is critical for long-term epidermal bioelectronic sensors and interfaces, as sustained skin contact necessitates minimal cytotoxicity and stable cell-material interactions. To assess cytocompatibility, in vitro cell viability assays were conducted on conventional PVA-IL gels and the DES-PVA eutectogels (Fig. 1h). PVA-IL gels incorporating commonly used ILs, including EMIMBF4, EMIMTFSI, and EMPTFSI, induced pronounced cellular damage, characterized by poor adhesion and markedly reduced viability. In contrast, DES-PVA eutectogels supported dense and uniform cell adhesion, maintaining cell viabilities above 90% at both 10 wt% and 50 wt% DES loadings. The well-spread cell morphology observed even at high DES contents highlights the superior cytocompatibility of the proposed eutectogels, underscoring their suitability as epidermal bioelectronics and biointerfaces." (**Section**: Results and Discussion, **Paragraph**: 3)

4. no sup information file was available.

Response: Thank you for raising this question. While our records indicate that the Supplementary Information was included in the initial submission, it may not have been correctly processed or made available during the review stage. To ensure seamless access, we have re-uploaded the Supplementary Information in this revised submission and verified that the file is properly attached to the system.

5. Figure 2 has too many images. It makes the reading very difficult. Figures must be separated and introduced everytime they are mentioned to make the reading easier.

Response: Thank you for the valuable suggestions. We have carefully re-evaluated the layout of Fig. 2 to ensure visual clarity and readability. In our manuscript, Fig. 2 is designed as an integrated unit where mechanical properties and piezoionic performance are presented together. This arrangement facilitates a direct correlation between mechanical deformation and piezoionic output, which is important for understanding the underlying mechanism. To maintain clarity while avoiding visual overcrowding, secondary or supporting data have been relocated to the Supplementary Information.

Furthermore, we have conducted a detailed quality check to ensure that all sub-panels, labels, and font sizes meet high-resolution standards and allow precise identification. The manuscript presents the sub-figures in a sequential manner, providing a structured narrative that guides the reader through the results. This integrated multi-panel format is commonly adopted in high-impact studies (***Nat. Electron.*** 2026, 7, 168; ***Nat. Mater.*** 2026, 25, 463; ***Nat. Commun.*** 2026, 17, 1150; ***Nat. Commun.*** 2026, 17, 2675; ***Nat. Commun.*** 2026, 17, 1150; ***Sci. Adv.*** 2025, 11, eadw6166; ***Adv. Mater.*** 2025, 37, 2412271) to consolidate interrelated evidence and maintain logical continuity.

We believe that this layout effectively highlights the relationship between material properties and device performance, thereby improving the clarity and coherence of the presentation.

6. Cytotoxicity assay (methodology). needs further details such as cell density.

Response: Thank you for the careful and insightful review. To assess the biocompatibility of the eutectogels, human prostate stromal myofibroblast (WPMY-1) cells were seeded in 6-well plates at a density of $1x10^6$ cells per well and incubated for 24 h to ensure adequate attachment. The eutectogel samples were then co-incubated with the cells for an additional 24 h. After incubation, cell viability was assessed by

confocal laser scanning microscopy using Calcein AM/PI dual fluorescence staining. The experimental groups included eutectogels containing 10 wt% and 50 wt% DES, while the control groups consisted of PVA-based IL (PVA–IL) gels containing 10 wt% EMIMBF4, EMIMTFSI, and EMPTFSI, respectively. The eutectogel with 50 wt% DES was specifically selected to evaluate cytotoxicity at elevated DES content, and the results confirm its excellent cytocompatibility. In addition, a blank control without any gel material was included to provide a baseline for comparison.

Our revision to the manuscript:

"To assess the biocompatibility of the eutectogels, human prostate stroma myofibroblast (WPMY-1) cells were seeded in 6 well plates with the initial density of $1x10^6$ cell per well." (**Section**: Methods, **Paragraph**: Cytotoxicity tests)

"Eutectogel samples containing 10 wt% and 50 wt% DES contents, together with control PVA-IL gels containing 10 wt% EMIMBF4, EMIMTFSI, and EMPTFSI, were prepared as standardized discs with a radius of 5 mm and a thickness of 1 mm." (**Section**: Methods, **Paragraph**: Cytotoxicity tests)

We sincerely thank the reviewer again for the insightful suggestions and the time devoted to evaluating our work. By refining the experimental description and improving the logical organization, we have enhanced the clarity and rigor of the manuscript. We believe that the revisions have effectively addressed the concerns raised.

Response to Reviewer #2

General comment: This paper fabricated a piezoionic hydrogel by using hydrogen bonds to selectively immobilize cations and enable rapid transport of anions. The piezoelectric effect was explained through DFT simulations and FTIR. However, the proposed gel and exhibited piezoionic mechanism seem not novel enough. Some declamations and interpretations did not provide reasonable discussions. In addition, the demonstrated physiological monitoring did not show new points. As a result, this paper should be rejected.

Response: We sincerely thank you for the critical evaluation and the constructive comments on the mechanism novelty and physiological applications of this work. In response, we have carefully revised the manuscript to strengthen the mechanistic discussion and improve the presentation of the physiological sensing results.

To address the novelty of the proposed mechanism, we have incorporated additional experimental data and established a more rigorous theoretical and experimental framework. As detailed in our responses to Comment 3 and Comment 7, we provide a systematic elucidation of the molecular-level ion-decoupling mechanism enabled by the synergistic hydrogen-bonding network within the PVA-DES matrix. This mechanism leads to a pronounced disparity in ionic mobility, resulting in a high piezoionic coefficient of 59.8 mV kPa^{-1}, which is higher than most reports (***Chem. Eng. J.*** 2026, 530, 173003; ***Angew. Chem. Int. Ed.*** 2025, 64, e202415000; ***Nat. Commun.*** 2024, 15, 10876; ***Adv. Mater.*** 2024, 36, 231317; ***Science*** 2022, 376, 502).

Regarding the physiological monitoring applications, we have expanded the discussion in our responses to Comments 4–6 to further clarify the signal fidelity and practical relevance of the epidermal sensors. Additional experimental results demonstrate that the eutectogels can maintain stable performance under various physiological mechanical stimuli, including arterial pulse and vocal cord vibrations, supporting their potential for reliable human signal monitoring.

We believe that these revisions, including the addition of new data and improved scientific descriptions, have addressed the concerns raised and strengthened the overall quality of the manuscript. The point-by-point responses to all comments are provided below.

1. In the Introduction, the authors argue that conventional piezoelectric materials suffer from limitations such as the lack of seamless integration, signal stability, and long-term

reliability. However, the present study does not convincingly demonstrate that these challenges have been resolved. In particular, long-term signal stability, which is central to the authors' motivation, is neither rigorously evaluated nor substantiated by sufficient experimental evidence.

Response: Thank you very much for the careful and insightful comment. We agree that the statement in the Introduction may have overgeneralized the resolution of these fundamental challenges. Accordingly, we have revised the corresponding sentence in the **Introduction** to more accurately reflect that our work specifically addresses the long-term signal stability of eutectogels for mechanical signal perception.

To further clarify the concept of seamless integration, we have incorporated additional mechanical analysis in the revised manuscript. The substantial modulus mismatch between conventional piezoelectric materials and soft biological tissues is effectively mitigated by the tissue-mimetic Young's modulus of our eutectogels. Conventional piezoelectric materials can generally be categorized into inorganic and organic systems. Rigid inorganic piezoelectric materials, such as PZT and BaTiO3, typically exhibit a high Young's modulus exceeding 1 GPa (***J. Phys. D: App!. Phys.*** 2022, 55, 423002; ***Adv. Eng. Mater.*** 2023, 25, 2201387; ***J. App!. Phys.*** 2018, 123, 094103), which severely limits their conformability to soft tissues. Organic piezoelectric materials, such as electrospinning of PVDF fibers, have achieved improved flexibility through advanced processing strategies, offer improved flexibility. However, their Young's modulus remains above 100 MPa (***Adv. Funct. Mater.*** 2025, 35, e15157; ***Adv. Funct. Mater.*** 2025, 35, 2509283; ***Adv. Fiber Mater.*** 2024, 6, 133). Although these organic materials have been explored for wearable electronics, their relatively high stiffness restricts conformal contact with skin, particularly under dynamic deformation. As a result, their applications are often focused on mechanical energy conversion rather than conformal bioelectrodes for stable physiological signal monitoring. In contrast, our eutectogels exhibit a Young's modulus comparable to that of human skin. By tuning the DES content, the modulus can be systematically adjusted, reaching approximately 1 MPa at 70 wt% DES, which falls within the typical range of skin (0.1–1 MPa). This mechanical softness, combined with stable elastic deformation up to 50% strain, ensures a conformable and intimate interface contact during dynamic physiological activities. These results highlight the mechanical advantages of the eutectogels compared with conventional piezoelectric materials, which support its suitability for bio-integrated sensing.

Regarding the concern on long-term signal stability, we have conducted additional

experiments to evaluate both operational durability and environmental stability. First, cyclic stability tests exceeding 500 continuous loading-unloading cycles were conducted to assess operational robustness, which was used to replace the original Fig. 2m. The results demonstrate that the piezoionic voltage and current remains highly consistent without observable signal attenuation or fatigue, confirming robust mechanical-to-electrical transduction of the eutectogel. Second, long-term aging tests were performed by re-measuring the same samples after storage under ambient conditions, as shown in Figs. R1 and R2. Benefiting from the non-volatile nature of the DES, the eutectogel avoids dehydration-induced degradation commonly observed in conventional hydrogels. Notably, after 6 months of storage, the signal amplitude shows minimal variation, with less than 5% performance decay and no observable change in cyclic response behavior. The results indicate that both voltage and current outputs remain highly stable, which demonstrate that the ionic network structure are well preserved over extended periods.

These multi-dimensional stability evaluations, including both cyclic durability and long-term aging, have been incorporated into the revised Supplementary Information. We believe these results provide a more comprehensive and rigorous demonstration that our eutectogels offer reliable performance for long-term bio-integrated sensing applications.

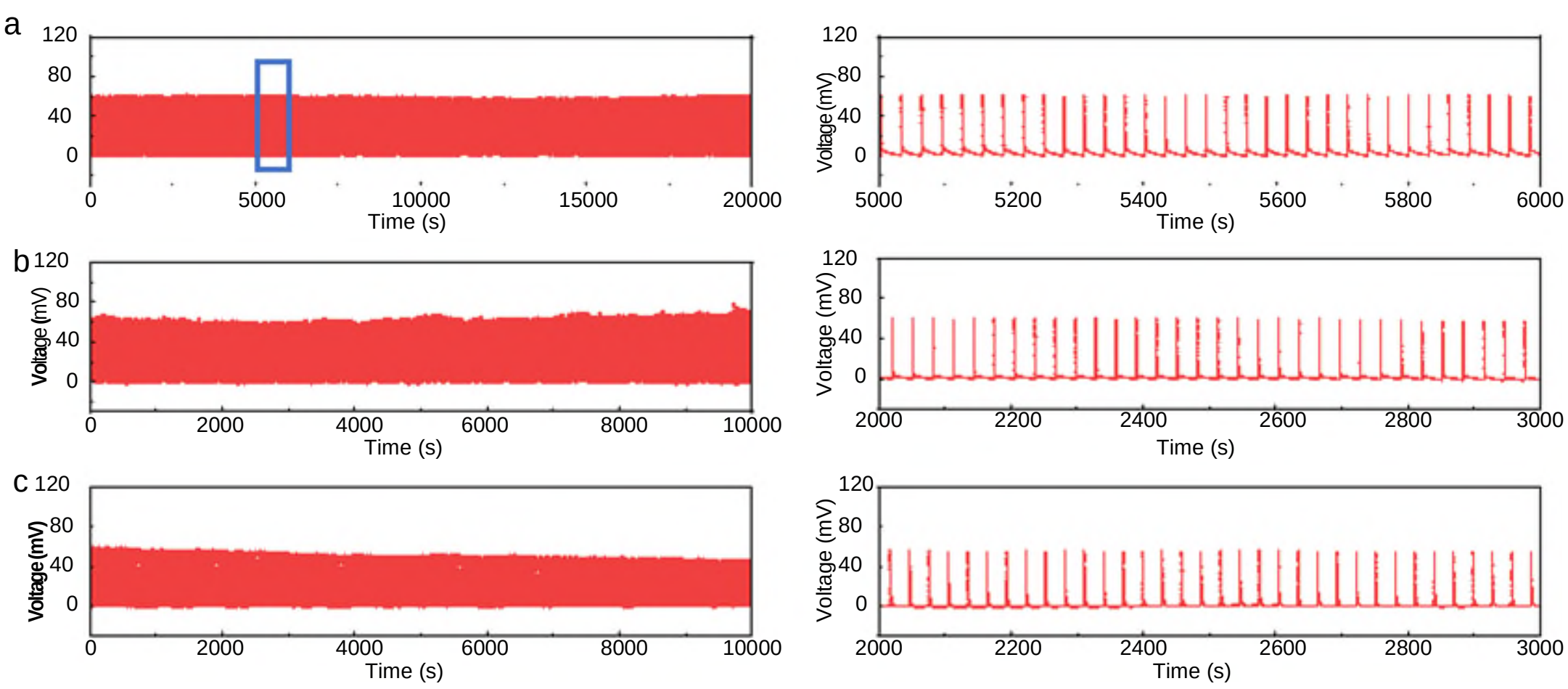

Fig. R1 Long-term voltage stability and cyclic response of the eutectogel at (a) the initial state, (b) after 3 months, and (c) after 6 months of storage, showing minimal performance and stability reduction.

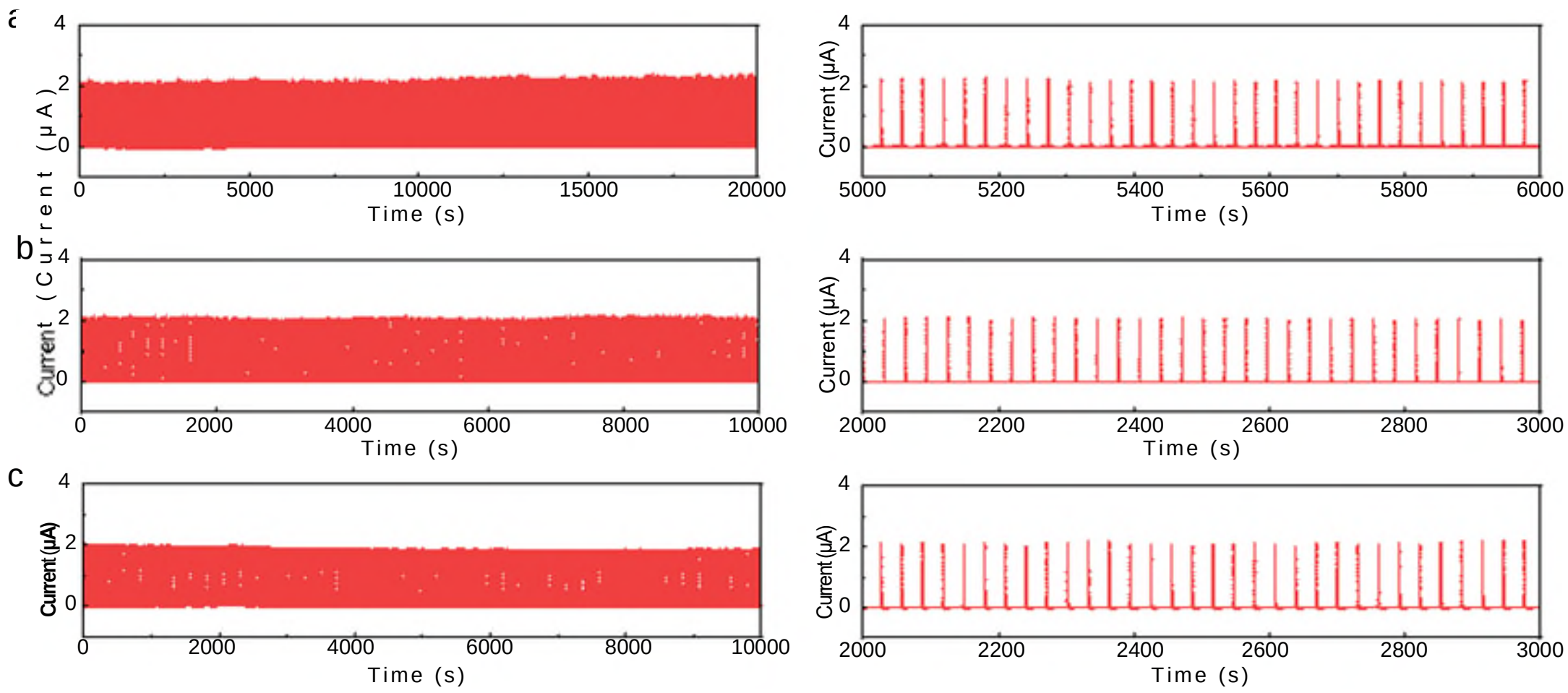

Fig. R2 Long-term current stability and cyclic response of the eutectogel at (a) the initial state, (b) after 3 months, and (c) after 6 months of storage, showing minimal performance and stability reduction.

Our revision to the manuscript:

We have revised the statement in the Introduction, and added Figs. R1 and R2 as Supplementary Figs. 14 and 15 in the revised manuscript and supplementary Information.

"Nevertheless, the high Young's modulus of most piezoelectric materials constitutes a fundamental mechanical mismatch with soft skin, which limits the realization of conformable and reliable interfaces for bio-integrated applications" (**Section**: Introduction, **Paragraph**: 1)

"Further, the piezoionic eutectogel exhibits excellent long-term stability and durability. As shown in Fig. 2m, both piezoionic voltage and current signals remain stable over 500 consecutive loading-unloading cycles, with negligible output drift, demonstrating robust durability under repeated mechanical stimulation. In addition, long-term stability tests reveal that the eutectogel maintains highly consistent electrical outputs after prolonged storage, with minimal signal attenuation observed over 6 months (Supplementary Figs. 14, 15). This long-term reliability is attributed to the non-volatile nature of the DES and the stable ionic network, which effectively preserve ion transport pathways and prevent dehydration-induced performance degradation." (**Section**: Results and Discussion, **Paragraph**: 7)

2. Figure 2c indicates pronounced creep behavior during the first tensile cycle, suggesting substantial viscoelastic relaxation. In contrast, Figure 2m reports highly stable piezoionic signals under cyclic loading. The manuscript does not reconcile this

apparent inconsistency. Without a mechanistic explanation or additional controls, the stability of the electrical response remains questionable.

Response: Thank you for the insightful comments. Upon re-evaluating the Figs. 2c and 2m, we find that the apparent inconsistency primarily arises from two fundamental differences: the **loading modes** and the **applied stress regime**. Accordingly, the results in intrinsic Fig. 2c are not directly suitable for evaluating piezoionic stability, as detailed in the explanation below.

First, the two measurements involve distinct loading modes: Tension and Compression. Fig. 2c presents the cyclic mechanical testing under tensile mode, where the polymer network undergoes significant uniaxial elongation. In this regime, the continuous dissociation and reformation of the dense hydrogen-bonding network led to pronounced viscoelastic relaxation. In contrast, the piezoionic stability shown in Fig. 2m was measured under compressive loading, which is directly relevant to pressure sensing conditions. Under compression, the deformation is more confined, allowing the hydrogen-bond network to recover more readily and resulting in a highly reversible mechanical response. To further substantiate this point, we performed additional cyclic compression tests over more than 200 cycles. The resulting stress–strain curves exhibit minimal hysteresis and nearly complete overlap (Fig. R3), providing a reliable mechanical basis for stable piezoionic output. Following your helpful comment, Fig. R3 has been used to replace Fig. 2c in the main manuscript, while the tensile cyclic data have been relocated to Supplementary Fig. 7b.

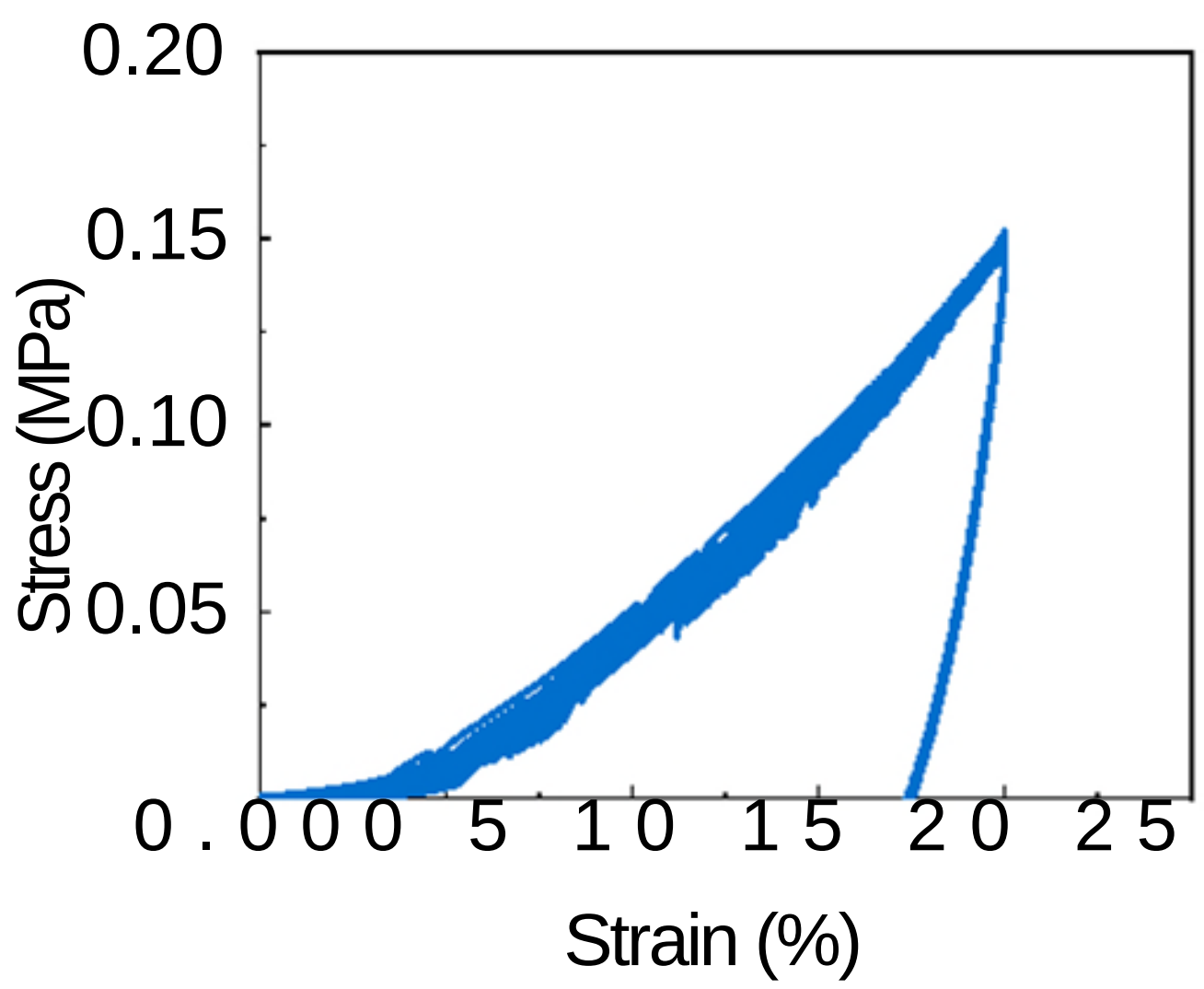

Fig. R3 Compression cyclic testing of the eutectogel under 20% compressive strain.

Second, the two measurements are conducted under significantly different

stress levels. The viscoelastic behavior in original Fig. 2c occurs under high tensile stress in the range of approximately 1–6 MPa. In contrast, the piezoionic measurements in Fig. 2m are performed under compressive pressures below 20 kPa (Fig. R4a), which are nearly two orders of magnitude lower than that of tensile stress in original Fig. 2c. Therefore, the tensile cyclic tests are intended to demonstrate mechanical robustness even under extreme stress-strain conditions, rather than to support the stable piezionic performance under low-pressure range. Under the low-pressure conditions, the eutectogel exhibits excellent mechanical recovery behavior, as clearly demonstrated by the compressive cyclic tests in Fig. R3. Upon enlarging the stress-strain curves in Fig. 2a within the 0.1 MPa range (Fig. R4b), we observe that the curves lie entirely within the elastic deformation regime. This indicates that our eutectogels can operates within a well-defined elastic region, even at stress levels higher than those applied in piezoionic measurements, thereby ensuring excellent mechanical recoverability and stable piezoionic signal generation under cyclic loading. These results indicate that the viscoelastic relaxation observed in tensile testing arises from high-stress deformation beyond the elastic regime, whereas the piezoionic measurements are conducted within the elastic regime, where mechanical stability is preserved.

In addition, the operational durability and environmental robustness in Figs. R1 and R2 further demonstrate the stability of both the mechanical and electrical responses. The additional compression data and corresponding analysis have been incorporated into the revised manuscript to clarify this point.

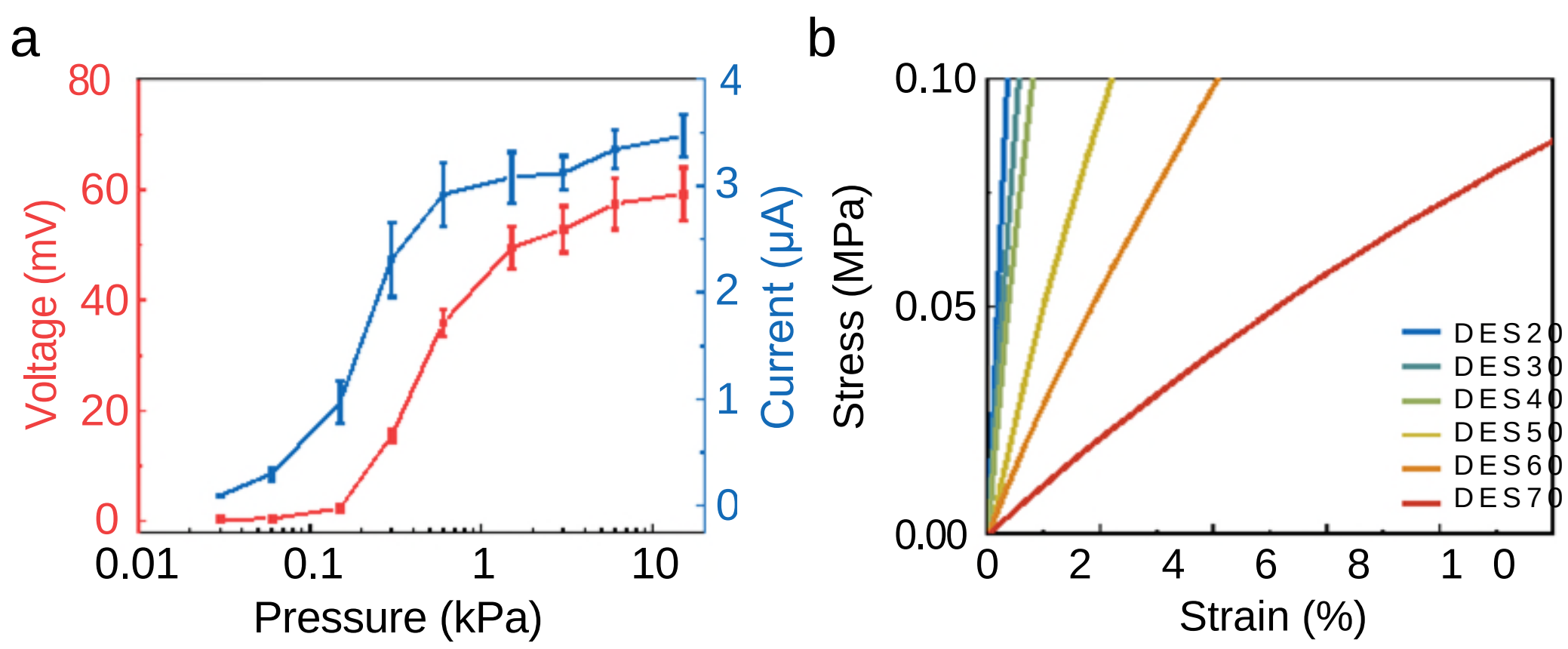

Fig. R4 (a) Piezoionic voltage and current of DES-PVA within a pressure range of 15 kPa. (b) Stress-strain responses across DES compositions.

Our revision to the manuscript:

We have added the Fig. R3 as Fig. 2c in the revised manuscript, and moved the original Fig. 2c into Supplementary Fig. 7b. The corresponding content has also added

in the revised manuscript.

"(c) Compression cyclic testing of the eutectogel under 20% compressive strain, where strain level was cycled above 200 times." (**Fig. 2c**)

"(g) Piezoionic voltage and current of the eutectogel within a pressure range of 15 kPa" (**Fig. 2g**)

"At the strain range of 10–200%, the eutectogels exhibit reproducible stress-strain responses over repeated cycles, indicating excellent recovery and mechanical stability under dynamic deformation. Furthermore, cyclic compression tests conducted under stresses below 0.15 MPa show highly stable responses with minimal hysteresis (Fig. 2c), confirming that the eutectogel maintains mechanical integrity under compressive loading. This mechanical stability contributes to the highly stable piezoionic response." (**Section**: Results and Discussion, **Paragraph**: 4)

3. Figure 3j is incorrectly cited as Figure 4j. More critically, the selection of the four characteristic wavenumbers (2998 cm^{-1}, 3278 cm^{-1}, 3291 cm^{-1}, and 3544 cm^{-1}) used in the 2DCOS analysis lacks justification. These spectral features are not clearly identifiable in Figure 3j, and the 2998 cm^{-1} band appears outside the displayed spectral window. The authors must clarify how these bands were determined and why they were chosen.

Response: Thank you for your careful review and constructive comments. We apologize for the incorrect citation to Fig. 3j, which has now been corrected in the revised manuscript. Regarding the selection of the four characteristic wavenumbers (2998, 3278, 3291, and 3544 cm^{-1}), we appreciate the opportunity to clarify the methodology by which these characteristic wavenumbers were identified. **These bands were not manually selected, but were directly identified from the two-dimensional correlation spectroscopy (2DCOS) analysis using the 2Dshige software**. Specifically, the wavenumbers correspond to the autopeak maxima in the synchronous correlation map, which represent the spectral regions most sensitive to the applied mechanical perturbation.

In our eutectogels, the O–H stretching region forms a broad and highly overlapped band due to multiple hydrogen-bonding environments involving PVA, Cl^-, Ch^+, and glycerol. However, these features are not clearly distinguishable in the conventional 1D spectra (Fig. 3i). The 2DCOS method enables extraction of correlated spectral variations and identification of responsive vibrational modes even when they are not visually resolvable in the original spectra. From the 2DCOS results, the

prominent asynchronous cross-peak were identified at (3544, 3291) and (3278, 2998), which were used to analyze the sequential response of different functional groups. The band assignments are consistent with the molecular structure of the DES-PVA network. The peaks at 3278 cm^{-1} and 3291 cm^{-1} correspond to hydrogen-bonded O-H stretching modes associated with glycerol-Cl^- and PVA-Ch+ interactions, respectively, while the band at 3544 cm^{-1} corresponds to relatively free O-H groups. The band at 2998 cm^{-1} originates from C–H stretching vibrations of the polymer backbone. The sequential evolution of these features was further determined using Noda's rule based on the signs of synchronous and asynchronous cross-peaks, providing insight into the redistribution of hydrogen-bond interactions within the system. The corresponding description has been added in Supplementary Note 5.

The limited visibility of these features in the original Fig. 3j mainly resulted from the plotting parameters, including the color scale and contour density. To address this, we have optimized the visualization by increasing the contour resolution and adjusting the color mapping (Fig. R5), which significantly improves the clarity of the spectral features. In addition, the identified wavenumbers have now been explicitly labeled in Fig. 3j to further enhance readability and facilitate direct comparison with the analysis. We also acknowledge that the 2998 cm^{-1} band was not included in the original spectral window. This has been corrected by extending the spectral range to 2950–3600 cm^{-1} in the revised 2DCOS maps, ensuring that all relevant features are fully captured and directly verifiable.

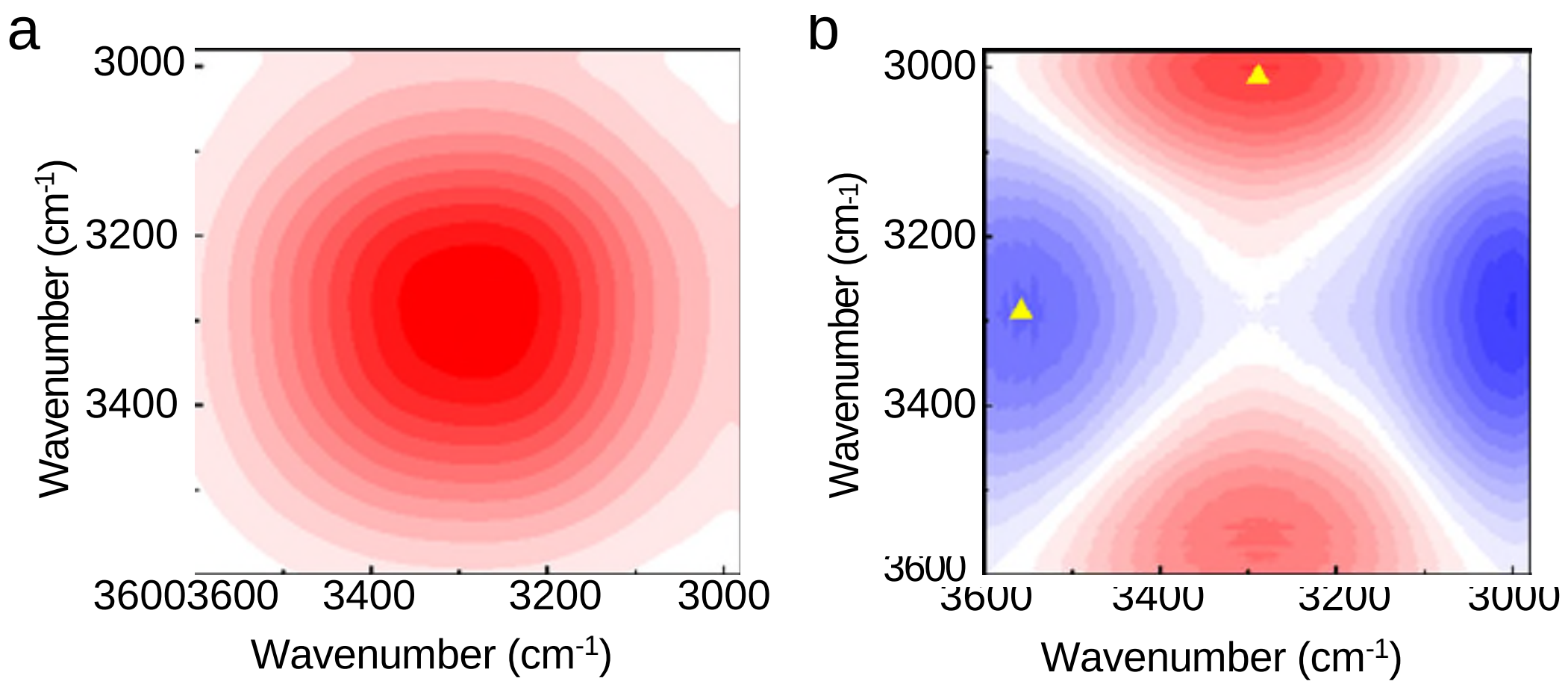

Fig. R5 2DCOS data: (a) synchronous and (b) asynchronous maps derived from pressure-dependent FTIR data, where red and blue regions represent positive and negative correlation, respectively.

Our revision to the manuscript:

"Due to the broad O-H stretching envelope comprises multiple overlapping hydroxyl sub-bands, two-dimensional correlation spectroscopy (2DCOS) was further employed to resolve the pressure-dependent responses of distinct hydrogen-bonding species (Fig. 3j and Supplementary Note 5)." (**Section**: Results and Discussion, **Paragraph**: 12)

"(j) 2D correlation spectroscopy (2DCOS) synchronous and asynchronous maps derived from pressure-dependent FTIR data, indicating the cross-peak coordinates at (3544, 3291) and (3278, 2998)." (**Fig. 3j**)

"**Supplementary Note 5. Two-dimensional correlation spectroscopy (2DCOS) analysis**

2DCOS was employed to analyze the pressure-dependent FTIR spectra of the eutectogel and to resolve overlapping O-H stretching vibrations. In our eutectogel, multiple hydrogen-bonding interactions among PVA, Cl^-, Ch^+, and glycerol molecules give rise to a broad and highly overlapped spectral envelope within the range of 2950–3600 cm^{-1}. As a result, conventional one-dimensional (1D) FTIR spectra do not enable the direct identification of individual spectral components or their responses to external perturbation. To address this limitation, 2DCOS analysis was performed using the 2Dshige software, which processes a series of dynamic spectra collected under controlled pressure variation. The method generates synchronous and asynchronous correlation maps based on the Hilbert transform of the spectral dataset. The synchronous map reflects the overall intensity variations of spectral features with respect to the external perturbation, whereas the asynchronous map provides information on the sequential order of spectral changes, enabling the resolution of overlapped bands.

In the present study, the characteristic wavenumbers were not assigned based on visual inspection of the 1D spectra, but were objectively identified from the 2DCOS results. Specifically, the diagonal autopeaks in the synchronous map were used to determine the spectral regions that exhibit the highest sensitivity to pressure. In addition, prominent cross-peaks observed in the asynchronous map were used to establish correlations between different vibrational modes. Based on these criteria, four representative wavenumbers at 2998, 3278, 3291, and 3544 cm^{-1} were extracted directly from the correlation maps. These spectral positions correspond to distinct molecular environments within the DES-PVA network. The bands at 3278 and 3291 cm^{-1} are attributed to hydrogen-bonded O-H stretching modes, corresponding to

glycerol-Cl^- and PVA-Ch^+ interactions, respectively. The band at 3544 cm^{-1} is assigned to relatively free O-H groups with weaker hydrogen bonding, while the band at 2998 cm^{-1} originates from C-H stretching vibrations of the polymer backbone. Although some of these features are not clearly distinguishable in the original 1D spectra due to band overlap, their presence is revealed through their strong correlation responses in the 2DCOS maps. The sequential response of these vibrational modes to mechanical perturbation was further analyzed using Noda's rule. When the synchronous (Φ) and asynchronous (Ψ) cross-peaks share the same sign, the change at ν1 precedes that at ν2; otherwise, the reverse sequence applies. This analysis enables identification of the relative sensitivity of different functional groups to pressure and provides insight into the evolution of hydrogen-bonding interactions within the eutectogel.

Overall, the 2DCOS analysis demonstrates that pressure induces a redistribution of hydrogen-bonding interactions within the DES-PVA network, thereby modulating the local ionic environment. This process plays a critical role in generating the asymmetric ion transport behavior responsible for the observed piezoionic response."

(**Section**: Supplementary Information, **Paragraph**: Supplementary Note 5)

4. The photograph in Figure 4c suggests that the strap used for encapsulation and assembly provides negligible back pressure. Given that the proposed piezoionic generation mechanism fundamentally relies on mechanically induced deformation, the experimental configuration appears insufficient to generate meaningful gel strain. The manuscript fails to adequately explain how effective deformation was achieved.

Response: Thank you for careful and important review. As shown in Fig. 4b (Fig. R6), the device configurations were clearly illustrated in the original submission. In this architecture, the encapsulation layer is positioned as the outermost layer, while the eutectogel is in direct contact with the skin. Therefore, the encapsulation does not interfere with the transmission of mechanical stimuli to the sensing layer. To further clarify this point, we have provided additional explanations regarding how the device configurations ensure reliable signal generation.

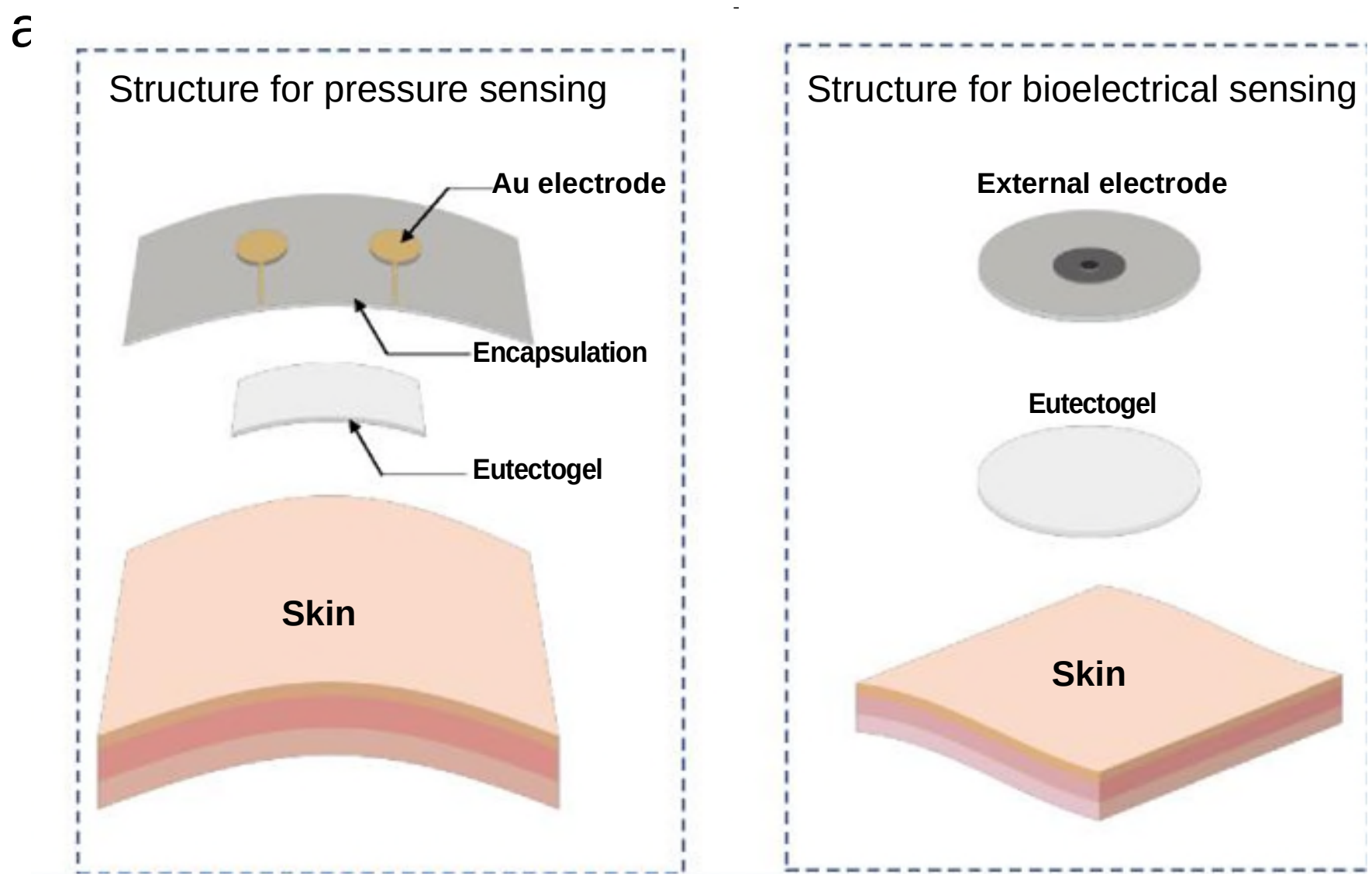

Fig. R6 Schematic illustration of the device architecture for (a) mechanical pressure sensing and (b) bioelectrical signal acquisition.

Specifically, the encapsulation layer consists of a biocompatible and single-sided adhesive tape with a thickness of approximately 48 µm. This layer serves solely as an external fixation component to maintain device positioning and ensure stable interfacial contact, rather than acting as a source of applied pressure. The eutectogel sensor is directly attached to the skin surface, with one end positioned over the vocal cord and the other on adjacent flat skin, where the two gold electrodes are located at two terminal positions to collect the resulting potential difference. The mechanical deformation required for signal generation originates from intrinsic physiological motion. During speech or swallowing, vocal cord vibration induces localized deformation of the overlying skin, generating compressive stresses on the order of 0.5–1.5 kPa. Importantly, our eutectogel demonstrates a detection threshold below 0.1 kPa (Fig. 2g), ensuring that these subtle physiological stimuli are well within the measurable range. Consequently, the deformation is sufficient to induce ion redistribution within the eutectogel and generate a detectable piezoionic signal.

In addition, we consider that the potential misunderstanding may stem from the matte nature of the adhesive tape, which can obscure the structural detaio of the epidermal sensor. To provide a more intuitive visualization, we have included additional photographs of sensors encapsulated with transparent polyurethane (PU) films (Figs. R7c and R7d), which exhibit nearly identical performance to those encapsulated with matte tape during application testing. This transparent encapsulation enables direct observation of the device–skin interface. It can be clearly seen that the

ultrathin PU film adheres stably to the skin surface, thereby allowing the encapsulated eutectogel to maintain intimate and stable contact with the skin. This configuration is therefore capable of efficiently transmitting mechanical stimuli associated with vocal cord motion and pulse.

(Redacted)

To further demonstrate that sufficient mechanical deformation can be effectively captured by our device configuration, we performed additional vocal cord vibration measurements across multiple participants. Specifically, vocal cord vibration measurements were performed using two adult male and two adult female volunteers, who were instructed to articulate the same word. As shown in Fig. R8, the recorded voltage signals exhibit highly consistent peak shapes and temporal patterns across different individuals. This high level of reproducibility indicates that the detected signals originate from intrinsic vocal cord-induced deformation rather than incidental noise or external perturbation. Importantly, although the vocal intensity differs between male and female participants, the overall waveform features remain well preserved, further confirming that the sensor reliably captures deformation-driven piezoionic responses. Moreover, the characteristic waveform associated with each phonation remains clearly identifiable across different participants, demonstrating that the deformation induced by vocal cord motion is sufficient and consistently transduced into

electrical signals. Beyond English numeric words, it was further demonstrated with English alphabet signature that the overall waveform profiles remain highly consistent across different individuals (Fig. R9). This reproducibility directly supports that the device configuration enables effective mechanical coupling between the skin and the eutectogel.

(Redacted)

These results confirm that the mechanical stimuli required for piezoionic signal generation originate from intrinsic physiological motion and are sufficient to induce reliable and measurable deformation in the eutectogel. These clarifications and additional results have been incorporated into the revised manuscript and collectively demonstrate that our device configurations enable efficient mechanical coupling and reliable physiological signal acquisition.

Our revision to the manuscript:

We have added Figs. R8 and R9 as Supplementary Figs. 23 and 24, respectively. Corresponding changes have been highlighted in yellow in the revised manuscript and supplementary materials.

"Benefiting from the intrinsic piezoionic response and favorable electrical properties of the eutectogels, two flexible device configurations were designed to enable the detection of both mechanical and bioelectrical signals in a skin-attachable platform (Fig. 4b), where ultrathin PU films are used as the outermost encapsulation layers to securely fix the electrodes and eutectogels onto the skin surface without hindering the transmission of mechanical stimuli." (**Section**: Results and Discussion, **Paragraph**: 13)

"As a representative example, the sensor was attached to the throat with the aid of a skin-adhesive PU film to monitor vocal cord vibrations during speech (Fig. 4c)." (**Section**: Results and Discussion, **Paragraph**: 14)

"As shown in Figs. 4d, 4e and Supplementary Fig. 22, each alphabet and number exhibit a characteristic waveform signature, enabling reliable subsequent classification. Furthermore, the highly similar signal waveforms obtained for the same word spoken by different individuals, including two male and two female participants, confirm the reliability of the sensor in identifying vocal cord vibration patterns (Supplementary Figs. 23, 24). These high-fidelity captures of subtle vibrational features highlight the potential of the piezoionic sensors for speech-related human-machine interfaces." (**Section**: Results and Discussion, **Paragraph**: 14)

5. The pulse waveforms presented in Figure 4g do not exhibit recognizable tidal wave characteristics, which are typically expected in physiological pulse signals. Additionally, the authors' claim that "This is the first demonstration of a piezoionic eutectogel ever reaching such high level of precision" is not supported by quantitative analysis or benchmarking.

Response: Thank you for the professional feedback. It should be noted that the T and D waves are intrinsically low-amplitude features that follow the primary percussion (P) wave and are therefore more susceptible to signal attenuation and acquisition limitations. **Benefiting from the high sensitivity of the eutectogel to low pressure, our piezoionic sensor is capable of resolving the T and D waves.** The indistinct wave characteristics in the original data primarily originate from two technical factors: the limited data acquisition rate (~25 Hz) and the point-based plotting representation. Under these conditions, discrete data points obscure fine variations in low-amplitude features, thereby making the T and D waves difficult to distinguish.

To address these limitations, we performed additional pulse measurements at an increased sampling rate (~50 Hz) and improved the data visualization by adopting a continuous line representation without point markers. This combined improvement effectively reduces the visual masking of local signal variations. As shown in the Fig. R10, the refined waveforms now clearly resolve the characteristic features of arterial pulse signals, including the P, T, and D waves, consistent with established physiological pulse profiles. We note that further increasing the sampling rate introduces additional instrumental noise, which can obscure low-amplitude features such as the T and D waves. Therefore, the selected acquisition conditions represent an optimized balance between temporal resolution and signal clarity.

We also appreciate your comment on the statement "This is the first demonstration of a piezoionic eutectogel ever reaching such high level of precision". Indeed, several eutectogels have been reported for pulse waveform monitoring (***Adv. Funct. Mater.*** 2024, 34, 2401808; ***ACS Appl. Mater. Interfaces*** 2021, 13, 20735), where most studies rely on piezoresistive sensing mechanisms for basic signal detection. In contrast, our piezoionic eutectogel sensor combines high sensitivity to low pressure with a self-powered sensing capability. To the best of our knowledge, eutectogels based on piezoionic mechanism have not yet been explored for monitoring physiological signals such as human pulse. To avoid potential ambiguity, the term "piezoionic eutectogel" has been used consistently in the original manuscript. Nevertheless, as pointed out by you, the use of terms such as "first" and "such high" may be overly definitive and could lead to potential misunderstanding. Accordingly, we have revised the sentence as follows: "These results demonstrate that piezoionic eutectogels represent a promising platform for pulse signal monitoring." The corresponding change has been highlighted in the revised manuscript.

(Redacted)

Our revision to the manuscript:

We have added Fig. R10 as Fig. 4g in the revised manuscript to include additional experimental data.

"The resulting voltage waveforms clearly resolve key features of the arterial pulse, including the percussion wave, tidal wave, and dicrotic wave (Fig. 4g). These results demonstrate that piezoionic eutectogels represent a promising platform for pulse signal monitoring." (**Section**: Results and Discussion, **Paragraph**: 14)

6. The realization of ECG acquisition in Figure 5i is insufficiently explained. According to the mechanical characterization (Figures 2a–c), the gel exhibits a Young's modulus significantly higher than that of human skin, implying poor conformability and suboptimal electrode-skin coupling. The authors do not address how reliable ECG signals were obtained despite this apparent mechanical incompatibility.

Response: Thank you for raising this important question. Indeed, modulus mismatch can impede conformability between skin and bioelectrode. In this work, our eutectogels achieve reliable ECG signals through a synergistic optimization of mechanical compatibility and ionic conductivity, as detailed below.

As shown in the mechanical characterization in Fig. 2b, the Young's modulus of the eutectogels can be effectively tuned by adjusting the DES concentration. Increasing the DES fraction reduces the effective crosslinking density of the PVA network and

softens the matrix due to the plasticizing effect of the solvent phase. As a result, the Young's modulus decreases from approximately 25 MPa to ~1 MPa (Fig. R11a). At a DES content of 70 wt% (DES70), the modulus (~1 MPa) approaches that of human skin (0.1–1 MPa), thereby enabling conformal contact and stable interfacial coupling during physiological monitoring.

More importantly, the stable and high-fidelity ECG acquisition is governed not only by mechanical matching but also by effective electrical coupling at the electrode– skin interface. Reliable biopotential recording requires minimized electrode-skin contact impedance, which is strongly dependent on the ionic conductivity of the electrode materials. In our eutectogels, the incorporation of the DES phase significantly enhances ionic mobility, leading to increased ionic conductivity with increasing DES content (Fig. R11b). Specially, DES70 exhibits a high conductivity of up to ~3 S cm^{-1}. The combination of appropriate mechanical compliance and high ionic conductivity enables low interfacial impedance and efficient signal transduction, thereby allowing precise resolution of the P–QRS–T complex in ECG signals. It should also be noted that although increasing the DES content further enhances ionic conductivity, it simultaneously leads to a pronounced deterioration in mechanical properties, which is detrimental to long-term stability. Therefore, DES70 represents an optimal balance between electrical performance and mechanical robustness, and was selected as the electrode interface in the ECG measurements.

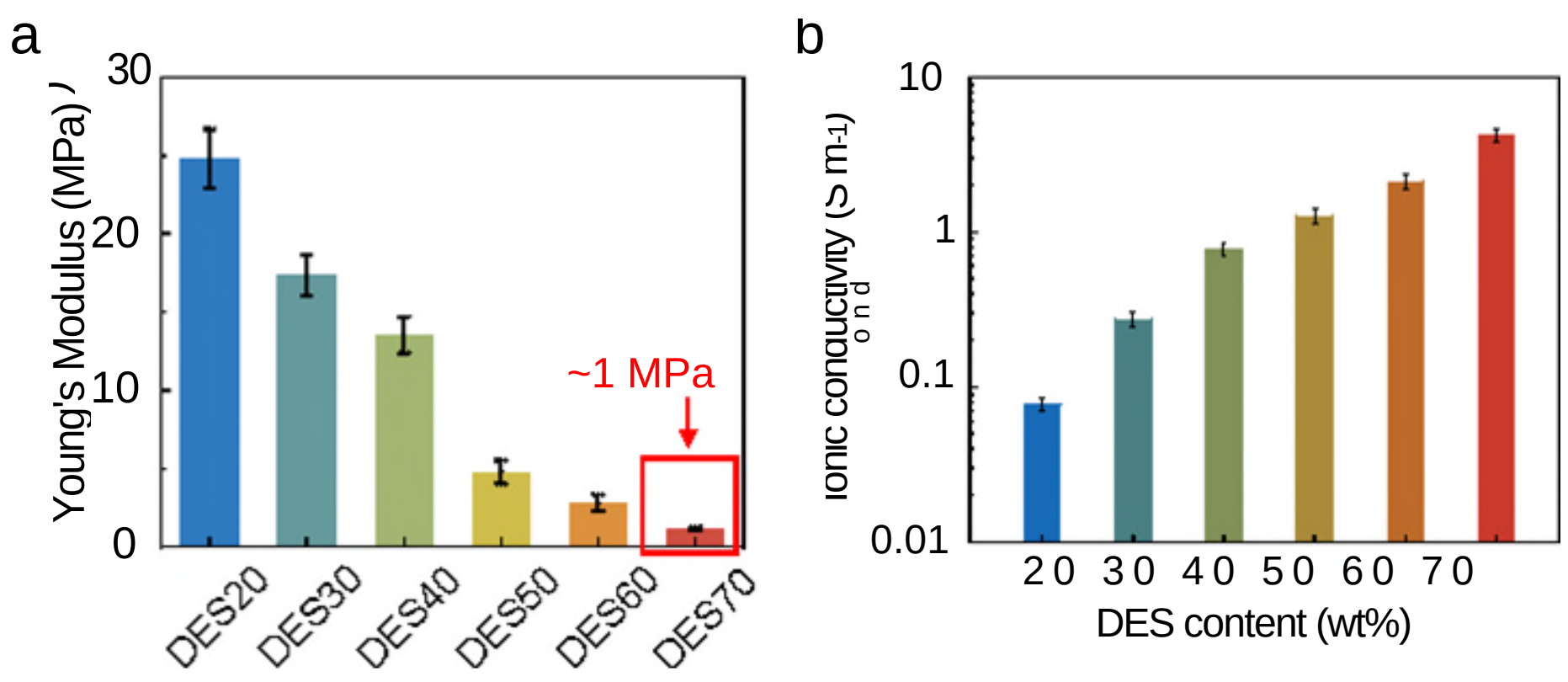

Fig. R11 (a) Young's modulus and (b) ionic conductivity of eutectogels as a function of DES content.

Our revision to the manuscript:

"(i) Comparison of ECG signals recorded using DES70 as electrodes against commercial hydrogel electrodes, showing comparable signal strength and fidelity." (**Fig. 4i**)

"Furthermore, DES70, combining mechanical compliance with high ionic conductivity, was employed as soft, skin-compatible electrodes for ECG data acquisition (Fig. 4h)." (**Section**: Results and Discussion, **Paragraph**: 15)

7. As we know, the ionic piezoelectric effects in gels usually occurs due to the relatively rapid movement of cations compared with anions. The cations have smaller sizes which the anions have larger sizes. However, in the manuscript, the authors proposed a different model.

Response: Thank you for your valuable question. We would like to emphasize that the piezoionic effect fundamentally originates from asymmetric ion transport induced by mechanical stimuli, where the difference in diffusion rates between cations and anions arises from ion size difference and polymer-ion interactions (***Nat. Commun.*** 2026, 17, 893; ***Adv. Mater.*** 2024, 36, 2313127; ***Science*** 2022, 376, 502). Evidently, the scenario noted by you, in which smaller cations migrate faster than larger anions, represents only one specific case rather than a general mechanism governing piezoionic signal generation.

To date, several studies have reported faster anion migration in piezoionic systems, driven either by larger cation size (***Adv. Funct. Mater.*** 2023, 33, 2210485) or by selective interactions with the polymer matrix (***Adv. Mater.*** 2024, 36, 2309821; ***Adv. Mater.*** 2024, 36, 2313127). In our study, the choline cation (Ch+) in choline chloride (ChCl) is a typical organic ion with an effective physical radius of approximately 3.29 Å, which is almost 1.8 times larger than that of the chloride anion (Cl^-, ~1.81 Å). This size disparity inherently favors faster migration of the smaller anions lelative to the larger cations. Beyond intrinsic size effects, our study further highlights the selective hydrogen-bond anchoring in amplifying ionic mobility asymmetry. As shown in Fig. R12a, DFT calculations reveal that Cl^- preferentially associates with glycerol due to its stronger binding energy, while Ch+ is more stably anchored to the PVA network via hydrogen bonding. This selective interaction provides a molecular-level basis for the distinct transport behaviors of the two ionic species.

Based on this interaction framework, molecular dynamics (MD) simulations were further performed to elucidate the pressure-dependent ion transport mechanism. Radial Distribution Function (RDF) analysis shows that the primary peak at approximately 4.8 Å corresponds to coulombic interaction between Ch+ and Cl^-. Upon applying pressure, the decreased peak intensity indicates weakened electrostatic attraction and progressive ion-pair dissociation. Meanwhile, the enhanced first RDF peak for Cl^--glycerol reflects

an increased coordination number, supporting the formation of a glycerol-mediated transport pathway that facilitates rapid anion migration. In contrast, the intensified first peak for Ch^+-PVA indicates strengthened hydrogen bonding, confirming enhanced cation immobilization within the polymer network. These results collectively indicate that a pronounced mobility asymmetry between Cl^- and Ch^+ is established under low pressure conditions, which is consistent with the experimentally observed rapid increase in piezoionic performance within the 0.1–1 kPa range. At higher pressure (20 kPa), the RDF peak intensities for Ch^+-Cl^-, Cl^--glycerol and Ch^+-PVA interactions remain essentially unchanged (Fig. R12), indicating that the system reaches a stabilized state. In this regime, the coordination between Cl^- and glycerol approaches saturation, resulting in a limiting ion migration rate. This behavior is in good agreement with experimental observations, where the piezoionic voltage and current show negligible increases beyond a certain pressure range.

Therefore, in our model, the faster migration of anions relative to cations originates from the synergistic effect of the larger effective size of the cations and their strong interactions with the polymer framework. This dual mechanism establishes a significant divergence in ionic mobility and results in a high piezoionic coefficient (59.8 mV kPa^{-1}). To improve clarity, the corresponding discussions have been further refined in the revised manuscript.

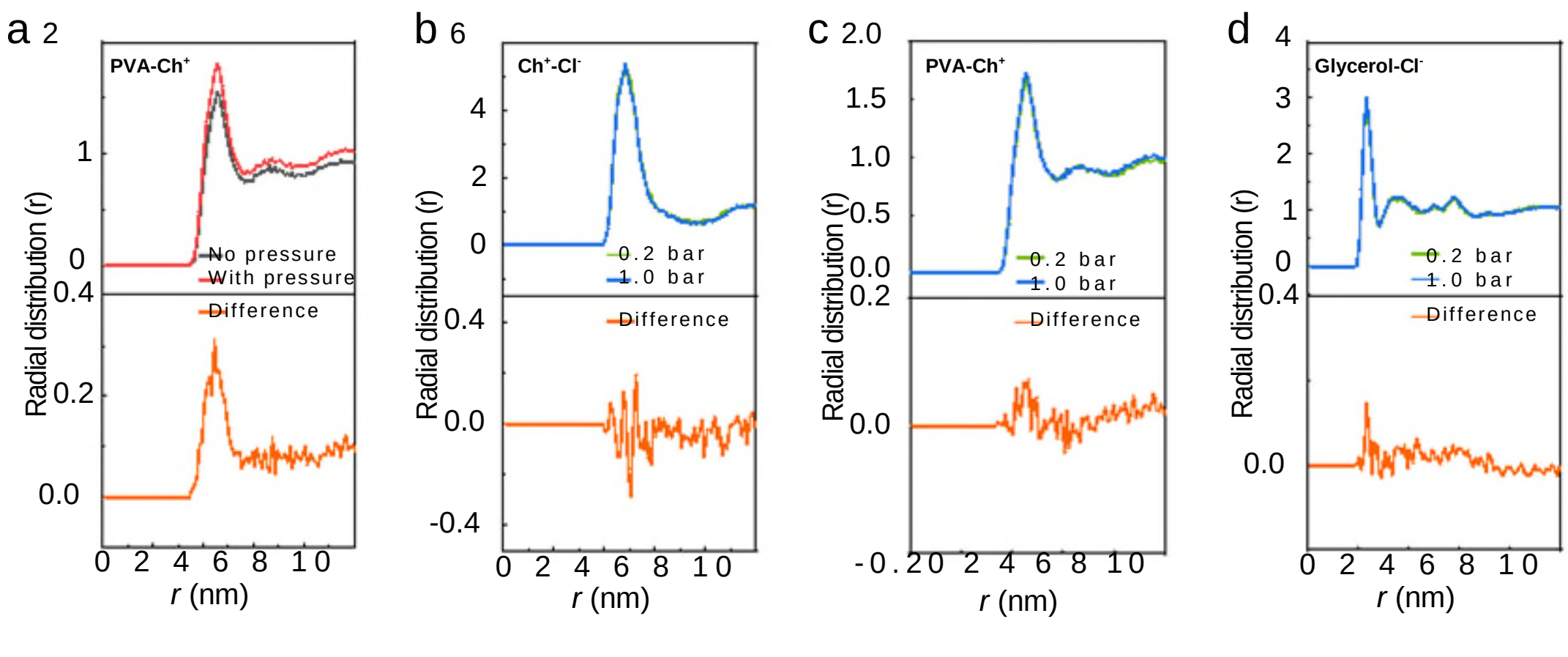

Fig. R12 (a) (a) RDF and calculated difference of PVA-Ch^+ under unloaded and pressurized conditions. (b–d) RDF and calculated difference of Ch^+-Cl^-, PVA-Ch^+ and glycerol-Cl^- pairs under two high pressure.

Our revision to the manuscript:

We have added Fig. R12 as Supplementary Fig. 17. Corresponding changes have been highlighted in yellow in the revised manuscript.

"When the applied pressure exceeds a certain threshold, the coordination number between Cl^- and glycerol reaches saturation. This equilibrium prevents further enhancement of Cl^- migration despite additional increases in pressure, thereby suppressing any further rise in the piezoionic voltage, which is consistent with the saturation of the piezoionic response beyond 10 kPa." (**Section**: Results and Discussion, **Paragraph**: 9)

"Notably, this peak becomes significantly more intense upon compression, reflecting an increased coordination of glycerol around Cl^-. This behavior indicates the formation of a continuous glycerol-mediated transport pathway that facilitates rapid Cl^- migration under pressure." (**Section**: Results and Discussion, **Paragraph**: 10)

"As shown in Supplementary Fig. 17, the increased peak intensity under compression indicates an elevated probability of Ch^+ being transiently coordinated to the PVA network, accompanying the pressure-induced weakening of Ch+-Cl^-electrostatic pairing. At the higher pressure, the RDF peaks corresponding to Ch+-Cl^-, Cl^--glycerol, and Ch+-PVA interactions remain essentially unchanged (Supplementary Figs. 17b–d), indicating that the local chemical structure becomes insensitive to further pressure increases. In particular, the coordination between Cl^- and glycerol approaches saturation, resulting in limited ionic mobility. This behavior is consistent with the experimental observations (Fig. 2g), where both the piezoionic voltage and current exhibit negligible increases beyond a certain pressure range." (**Section**: Results and Discussion, **Paragraph**: 10)

"Together, the RDF and MSD analyses clearly reveal that external pressure regulates the balance between ion-dipole coordination and hydrogen bonding within the eutectogel network. Strengthened glycerol-Cl^- coupling promotes rapid Cl^- migration within the glycerol phase, whereas the PVA-Ch+ interaction more effectively immobilize the Ch^+ within the PVA framework. The combined effects of ionic size disparity and selective hydrogen-bond immobilization form the dynamic basis for the pressure-driven piezoionic behavior of the DES-PVA eutectogels." (**Section**: Results and Discussion, **Paragraph**: 11)

8. In Fig. 2i, the gel shows a larger resistance (~9 kΩ). It is known to all that low internal resistance of gels can facilitate the ECG measurement. Why can this gel be used in ECG experiments? How did the authors think about this?

Response: Thank you very much for this insightful comment. We agree that the

low internal resistance is important for high-quality ECG recording. However, the value reported in Fig. 2i represents a specific measurement under direct current (DC) conditions for piezoionic output evaluation, which does not reflect the intrinsic ionic conductivity of eutectogel for bioelectrical signal acquisition. We have provided the following clarifications to address this concern.

The apparent discrepancy arises from the different measurement modalities employed for distinct physical processes. The resistance of approximately 9 kΩ in Fig. 2i was obtained using a two-probe DC method, where the measured value includes substantial contributions from interfacial polarization and contact resistance. These interfacial effects can be several orders of magnitude higher than the intrinsic ionic resistance determined by electrochemical impedance spectroscopy (EIS) (***Nat. Commun.*** 2025, 16, 3755; ***Adv. Funct. Mater.*** 2025, 35, e08859; ***Nat. Commun.*** 2025, 16, 3755; ***Adv. Mater.*** 2024, 36, 2405372; ***ACS Nano 2024***, 18, 4008). In contrast, ECG signal acquisition depends on effective ionic conduction at the electrode–skin interface, which is more accurately characterized by EIS under alternating current (AC) conditions. As shown in Fig. 2h, the EIS results indicate that the DES70 eutectogel exhibits a high ionic conductivity of approximately 3 S m^{-1}, confirming its suitability for efficient bioelectrical signal monitoring (***Adv. Mater.*** 2026, 38, e23019; ***Nat. Commun.*** 2025, 16, 9454; ***Adv. Mater.*** 2024, 36, 2405556; ***Nat. Commun.*** 2023, 14, 219). This high intrinsic conductivity ensures low interfacial impedance and reliable signal acquisition during ECG monitoring.

Furthermore, it is important to distinguish between the DES formulations optimized for different functions. DES50 was employed in the piezoionic output measurements (Fig. 2i) to balance the piezoionic coefficient and internal resistance. In contrast, DES70 was specifically selected for ECG measurements due to its higher ionic conductivity and lower Young's modulus. This combination provides both efficient electrical coupling and sufficient mechanical conformability for stable integration with the skin, enabling high-fidelity ECG signal acquisition.

Therefore, the reported resistance value does not represent an intrinsic limitation of the eutectogel for ECG measurement because of the measurement method and the specific composition used. To avoid misunderstanding, we have further clarified these points in the revised manuscript.

Our revision to the manuscript:

"Considering the simultaneous requirements for mechanical robustness and

efficient piezoionic transduction, DES50 was selected for piezoionic output evaluation." (**Section**: Results and Discussion, **Paragraph**: 5)

"Furthermore, DES70, combining mechanical compliance with high ionic conductivity, was employed as soft, skin-compatible electrodes for ECG data acquisition (Fig. 4h)." (**Section**: Results and Discussion, **Paragraph**: 15)

"Power output was calculated by multiplying the current and voltage across the external load, which was specifically matched to the internal resistance of the eutectogel measured under direct current." (**Section**: Methods, **Paragraph**: Characterization and measurements)

"Electrochemical impedance spectroscopy (EIS) was collected at room temperature using an electrochemical workstation (PARSTAT2273) in a 0.1–1 MHz frequency range with a 10 mV AC perturbation to evaluate the intrinsic ionic conductivity relevant to both piezoionic conduction and ECG monitoring." (**Section**: Methods, **Paragraph**: Characterization and measurements)

9. ECG tests do not require power supply at all. They merely collect the signals of the heart's beats. How did the authors interpret the concept of self-powered?

Response: Thank you for careful comment. In our previous statement, the "self-powered epidermal sensors" specially refers to the piezoionic sensing mode, where mechanical stimuli are directly converted into electricals without the need for an external power supply. As you have mentioned, ECG monitoring relies on the acquisition of intrinsic bioelectrical signals generated by cardiac activity and does not involve energy harvesting by the sensor.

In the application of bioelectrical signal monitoring, the eutectogel functions exclusively as an ionic bioelectrode, enabled by its high ionic conductivity and appropriate Young's modulus. Therefore, the term "self-powered" is not intended to describe the ECG measurement process, but is strictly associated with the piezoionic pressure-sensing functionality. To avoid ambiguity and ensure clarity, this distinction has been explicitly clarified in the revised manuscript as follows: "**In addition to the aforementioned self-powered sensing applications, the eutectogels can be further employed for electrophysiological monitoring due to their excellent ionic conductivity**." This revision clearly delineates the two operating modes: the piezoionic sensing mode, which actively converts mechanical stimuli into electrical signals, and the electrophysiological monitoring mode, in which the eutectogel serves as a passive ionic electrode for signal acquisition.

Furthermore, we have performed a thorough check of the manuscript to ensure the accurate use of the term "self-powered".

Our revision to the manuscript:

"In addition to the aforementioned self-powered sensing applications, the eutectogels can be further employed for electrophysiological monitoring due to their excellent ionic conductivity. Furthermore, DES70, combining mechanical compliance with high ionic conductivity, was employed as soft, skin-compatible electrodes for ECG acquisition (Fig. 4h)." (**Section**: Results and Discussion, **Paragraph**: 15)

"Collectively, these demonstrations verify that the DES-PVA eutectogel biosensor provide multifunctional capabilities for the accurate monitoring of diverse mechanical and bioelectrical physiological signals." (**Section**: Results and Discussion, **Paragraph**: 16)

"For demonstrating practical applications, the eutectogel sensor in the form of a high-performance epidermal device monitors both mechanical and bioelectrical human physiology, with a high signal quality" (**Section**: Conclusion)

10. In Fig. 3g, the difference between two states seems not distinct. Could the simulations support the declaimed result?

Response: Thank you for meticulous comment. The relatively small variation in the total number of hydrogen bonds observed in Fig. 3g is consistent with the nature of the system under purely mechanical deformation, where the overall hydrogen-bonding network is largely preserved in the absence of compositional changes or structural disruption.

In MD simulations, significant changes in hydrogen-bond populations are typically associated with phase transitions or alterations in chemical composition. In contrast, the eutectogel in this work undergoes mechanical compression without introducing new chemical species or inducing large-scale structural rearrangement. As a result, the total hydrogen bond count exhibits only modest variation. Despite this limited change, the simulations reveal important local structural dynamics. RDF results (Fig. 3f) reveal that applied pressure promotes increased coordination between Cl^- and glycerol, which contributes to a slight increase in hydrogen-bond population. However, this increase remains small relative to the total number of hydrogen bonds, particularly during the initial compression stage (Fig. R13), and is consistent with the subtle changes observed in the first RDF peak.

Mechanistically, this behavior reflects a pressure-induced microscale densification of the hydrogen-bond network, rather than a fundamental structural transformation. The flexible supramolecular network, dominated by hydrogen bonding, accommodates deformation without significant disruption, leading to only minor variations in the total hydrogen-bond count. This is manifested in Fig. 3g as a measurable but limited change. As the simulation progresses, the hydrogen bond population gradually returns toward equilibrium, indicating relaxation of the system following deformation. Importantly, although the overall change in hydrogen-bond number is small, the corresponding redistribution of hydrogen-bonding interactions significantly affects local chemical environments and ion mobility. It should be noted that the MD results presented here are intended to provide qualitative insight into pressure-induced molecular dynamics rather than rigorous quantitative evaluation. Following your suggestion, we have clarified these points in the revised manuscript.

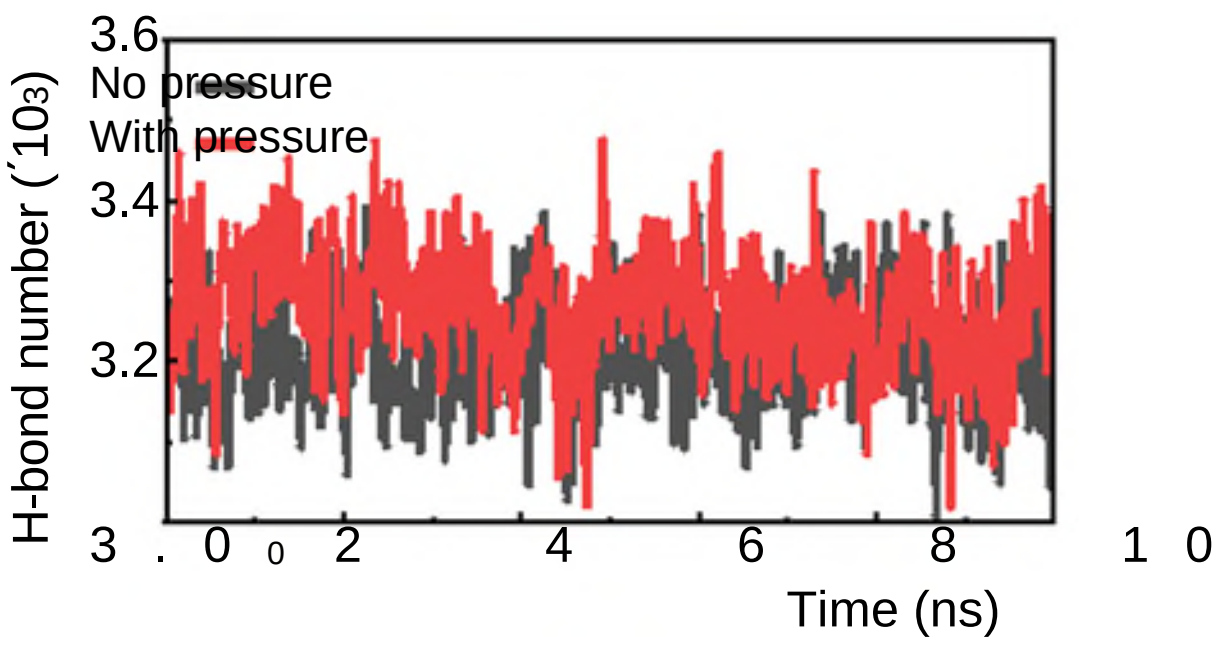

Fig. R13 Temporal evolution of the total number of hydrogen bonds in the eutectogel during compression, with an amplified y-axis in the MD simulation.

Our revision to the manuscript:

"This behavior is consistent with the experimental observations (Fig. 2g), where both the piezoionic voltage and current exhibit negligible increases beyond a certain pressure range. The increase in hydrogen bond population is consistent with the strengthened glycerol-Cl^- coordination revealed by the RDF profiles (Fig. 3g). This subtle variation suggests that the applied pressure primarily induces microscale densification of the hydrogen bond network without reaching the threshold for structural reorganization." (**Section**: Results and Discussion, **Paragraph**: 10)

11. In Fig. 3e, how did the authors obtain the result between unloading and pressurized states?

Response: Thank you for raising this question. We apologize for the unclear description in the caption of Fig. 3e. The comparison between the unloaded and

pressurized states was in fact presented in Supplementary Fig. 16, rather than in Fig. 3e. Fig. 3e is intended to serve as a representative structural model illustrating the molecular components and their initial spatial arrangement within the eutectogel framework. Our original intention was to indicate that this model would be subjected to mechanical compression in the simulations to enable comparison between unloaded and pressurized states. This has now been clarified in the revised manuscript.

The actual comparison between unloaded and pressurized conditions is derived from the MD configurations shown in Supplementary Fig. 16, where representative snapshots of the system are explicitly presented. These simulations capture the pressure-induced redistribution of ionic species within the polymer matrix. In the unloaded state, ions and solvent molecules exhibit a relatively uniform spatial distribution. Upon the application of pressure, a localized ion concentration gradient emerges in the compressed region. Specifically, the simulations reveal a pronounced decrease in the local concentration of chloride anions, whereas the distribution of choline cations shows a comparatively smaller change. This differential ion redistribution reflects the mobility asymmetry between the two ionic species and represents the fundamental origin of the piezoionic effect in our system. The distinct responses of Cl^- and Ch+ are consistent with their differences in effective size and interaction strength within the PVA matrix.

Our revision to the manuscript:

"(e) Atomistic models of the DES-PVA eutectogel for MD simulations, with a partially enlarged view of the solvation structure. Mechanical compression is applied to the simulation box to enable comparison between unloaded and pressurized states, as shown in Supplementary Fig. 16" (**Fig. 3e**)

We thank again for these insightful suggestions and the significant time in evaluating our work. Through the integration of additional experimental data and more rigorous scientific descriptions, the technical clarity and logical framework of this manuscript have been substantially strengthened. These refinements, coupled with the logical reorganization of the content, effectively address concerns regarding the underlying physical mechanisms, output performance, and piezoionic applications. We believe that the revised manuscript provides a more robust contribution to the field and now aligns with the high standards of ***Nature Communications***.

Response to Reviewer #3

General comment: In this study titled "Biocompatible Piezoionic Eutectogel Biosensor for Soft Self-Powered Human Physiological Diagnostics", the authors performed some attractive and valuable research. The developed eutectogels were characterized and applied successfully as Piezoionic biosensors. Few comments for further improvement are here;

Response: We sincerely thank the reviewer for the careful evaluation of our manuscript and for the positive comments on our work. We are particularly encouraged by your description of our study as "attractive and valuable research," which accurately recognizes the central achievement of this work, namely the successful development and application of eutectogels as piezoionic biosensors. We have carefully considered all of your constructive suggestions and have addressed them in detail in the revised manuscript. Our point-by-point responses to each comment are provided below.

1. The current advanced bioelectonic devices require a high adhesive strength, self-healing behavior, etc., the authors performed no such study.

Response: Thank you for the valuable feedback. We acknowledge that the eutectogels in this work do not exhibit intrinsic adhesion and self-healing capabilities. Nevertheless, owing to the skin-like Young's modulus and high ionic conductivity, these eutectogels remain well suited for bioelectronic applications. The following points clarify their effectiveness as bioelectronic interfaces.

First, excellent mechanical conformability, combined with an auxiliary fixation layer, ensures stable interfacial contact. Although the eutectogel itself does not possess intrinsic adhesion, its Young's modulus (~1 MPa) and high flexibility enable intimate contact with human skin, which is sufficient to minimize contact impedance for high-fidelity signal acquisition. To further enhance stability, the eutectogel is secured using a biocompatible ultrathin polyurethane (PU) adhesive layer. This layer serves solely as an external fixation component and does not interfere with bioelectrical signal transmission. As shown in the Fig. R14, this integrated configuration enables stable signal acquisition during continuous monitoring. This design strategy, which separates the functional sensing layer from the fixation layer, allows independent optimization of electrical performance and mechanical stability.

Second, the intrinsic mechanical robustness of the eutectogel minimizes the need for self-healing functionality. Self-healing is often required for materials that are prone

to mechanical failure or fatigue-induced cracking. In contrast, the eutectogel exhibits a high fracture strength (>5 MPa), which is significantly higher than the typical stress experienced by human skin (10–100 kPa). In addition, our eutectogels maintains structural integrity under repeated large-strain deformation (Fig. R15). This mechanical resilience ensures reliable operation under cyclic loading, thereby reducing the necessity for self-healing behavior compared to mechanically fragile hydrogel systems.

Finally, it is important to note that adhesion and self-healing are additional material functionalities rather than prerequisites for bioelectrical signal acquisition. In this work, reliable physiological signal monitoring is primarily enabled by mechanical conformability and efficient ionic conduction

Fig. R15 (a) Cyclic tensile loading-unloading tests at different strain levels. (b) Cyclic compression tests of the eutectogels under 20% compressed strain.

(Redacted)

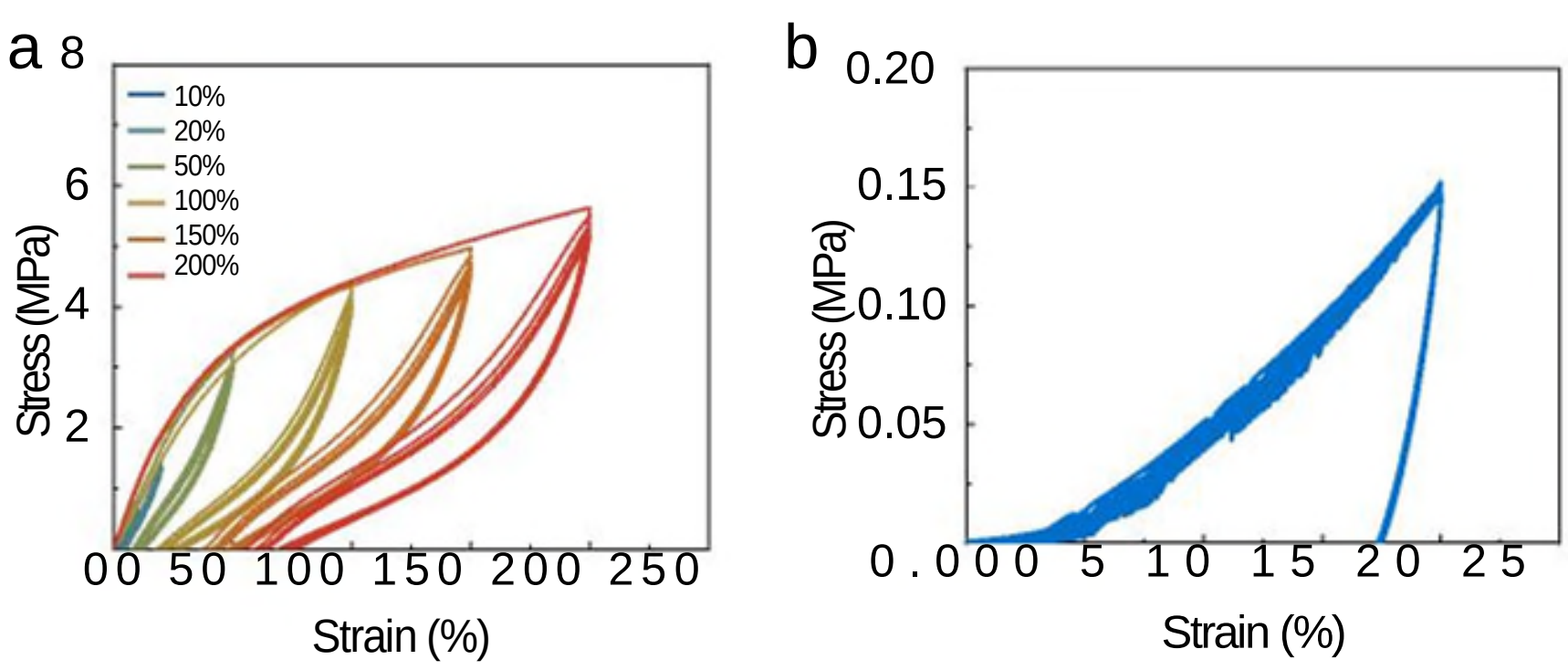

2. The vocal cord vibrational sensitivity was elaborated towards different alphabets. It will be better to check the same words articulated by different people. To confirm the selectivity of the sensor towards vibration patterns of the vocal cords exactly.

Response: Thank you for this constructive suggestion. To address this, additional experiments were performed to evaluate the vocal cord vibration signals across different individuals under identical articulation conditions. Specifically, four participants, including two males and two females, were asked to pronounce the same words, and the corresponding signals were recorded in Fig. R8.

The results show a high degree of consistency in the recorded vibration patterns across different individuals. As shown in Fig. R8, the characteristic peak shapes and temporal profiles are well preserved, indicating that the sensor reliably captures intrinsic vocal cord vibration features rather than individual-specific variations. This reproducibility confirms the robustness of the piezoionic sensing mechanism in detecting phonation-induced mechanical signals. While the overall waveform features remain consistent, differences in signal amplitude are observed between male and female participants. Specifically, the peak amplitudes recorded from female participants are generally lower than those from male participants, which is consistent with known physiological differences in vocal cord structure and vocal intensity. Importantly, these amplitude variations do not affect the preservation of the waveform patterns. Furthermore, this consistency is also observed beyond numeric words. As shown in Fig. R9, the waveform profiles obtained during English alphabet articulation remain highly similar across different individuals, further demonstrating the reproducibility of the sensor response.

These results collectively demonstrate that the eutectogel sensor enables reliable and repeatable detection of vocal cord vibrations across different subjects. The corresponding data and analysis have been added to the revised manuscript.

(Redacted)

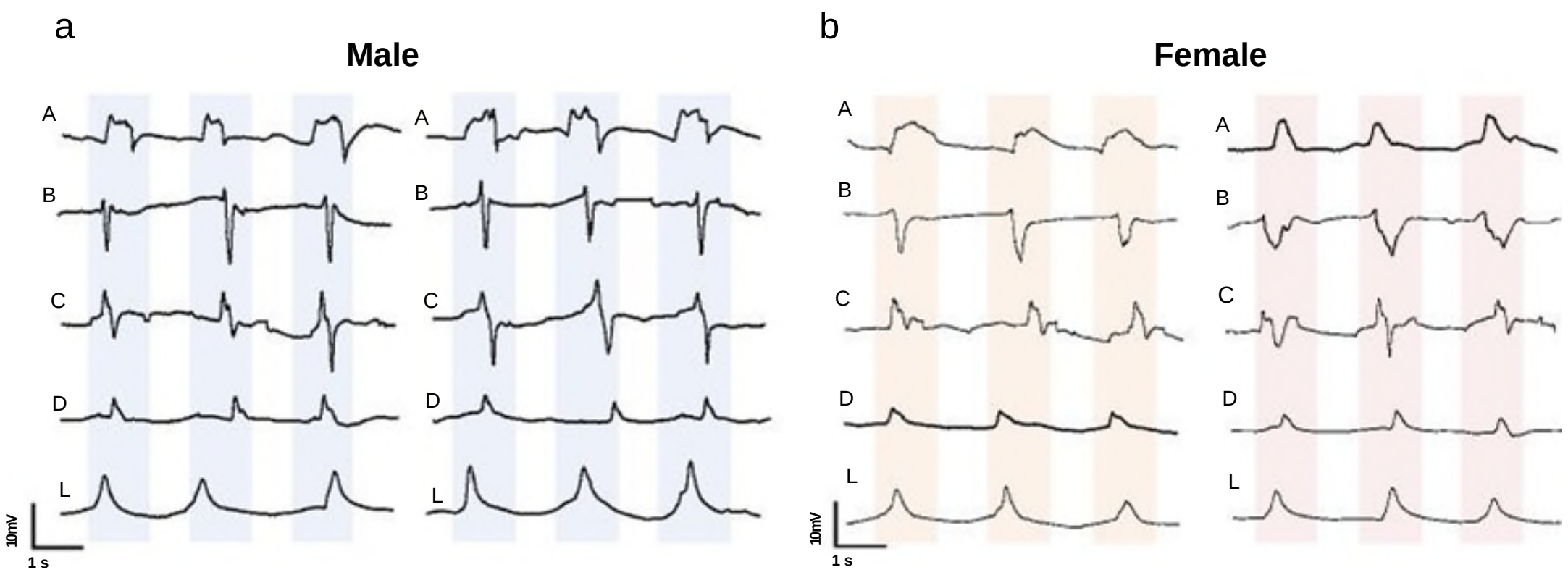

Fig. R9 (a) Piezoionic voltage signals generated during English alphabet articulation captured by the eutectogel electrodes for (a) two male and (b) two female test particpants.

Our revision to the manuscript:

We have added Figs. R8 and R9 as Supplementary Figs. 23 and 24, respectively. Corresponding changes have been highlighted in yellow in the revised manuscript and supplementary materials.

"As shown in Figs. 4d, 4e and Supplementary Fig. 22, each alphabet and number exhibit a characteristic waveform signature, enabling reliable subsequent classification. Furthermore, the highly similar signal waveforms obtained for the same word spoken by different individuals, including two male and two female test participants, confirm the reliability of the sensor in identifying vocal cord vibration patterns (Supplementary Figs. 23, 24). These high-fidelity captures of subtle vibrational features highlight the potential of the piezoionic sensors for speech-related human-machine interfaces." (**Section**: Results and Discussion, **Paragraph**: 14)

3. In supplementary Fig. 5, the notations (a, b, c,..g) are not described.

Response: We have added individual captions for each panel in Supplementary Fig. 5 and provided corresponding descriptions below the figure to improve clarity and readability.

Our revision to the manuscript:

"Supplementary Fig. 5 Raman spectra corresponding to the characteristic vibrational bands of the selected functional groups. (a) C-N groups of Ch^+. (b) C-O groups of PVA and glycerol. (c) $-CH_2$ and $-CH_3$ groups of PVA, Ch^+, and glycerol. (d) $-CH_2$ groups of PVA and Ch^+. (e) $-CH_3$ and $-CH_2$ groups of Ch^+ and glycerol. (f) $-CH_3$ groups of Ch^+. (g) -OH groups"

"As the DES content increases, the Raman spectra display systematic and correlated variations across multiple spectral regions, indicating progressive incorporation of DES components accompanied by the reorganization of the PVA network. The characteristic bands associated with choline become progressively more prominent, including the C-N vibration near 720 cm^{-1} and the N-CH3 stretching mode around 3040 cm^{-1} (Supplementary Figs. 5a and 5f). Notably, the positions of these peaks remain essentially unchanged, indicating that the incorporation of Ch+ does not perturb its local covalent configuration. In parallel, the band centered at approximately 1100 cm^{-1}, assigned to C-O vibrations, exhibits both increased intensity and spectral broadening (Supplementary Fig. 5b). This behavior is consistent with a broader distribution of hydrogen-bonding interactions and solvation environments involving PVA chains and glycerol molecules. A similar trend is observed for the deformation band near 1450 cm^{-1} (Supplementary Fig. 5c), corresponding to -CH2 and -CH3 groups, where the enhanced intensity and peak broadening suggest increased structural heterogeneity and more diverse local packing states upon DES incorporation. In the C-

H stretching region, the band at approximately 2920 cm^{-1}, associated with PVA and choline environments, gradually decreases with increasing DES content, whereas the band near 2980 cm^{-1}, attributed to -CH3 and -CH2 groups from choline and glycerol, becomes progressively stronger (Supplementary Fig. 5d and 5e). These opposite trends indicate that the increasing DES fraction not only alters the relative spectral contributions of the constituent components, but also reflects enhanced chain relaxation and segmental mobility within the eutectogel network. At higher wavenumbers, the broad -OH stretching envelope centered around 3400 cm^{-1} becomes both more intense and significantly wider (Supplementary Fig. 5d), suggesting an expanded distribution of hydrogen-bonding configurations arising from interactions among PVA hydroxyl groups, glycerol hydroxyl groups, and chloride anions. Overall, these spectral evolutions indicate that increasing DES content primarily modulates the hydrogen-bonding landscape and local microenvironments within the eutectogel, rather than inducing any alteration of the underlying covalent framework." (**Section**: Supplementary Information, **Paragraph**: Supplementary Fig. 5)

4. The authors stressed in introduction that before this study no Eutectogels are used for sensing purpose. However, such sentences should be revised. Because the literature is available on Eutectogels used for strain sensing applications.

Response: Thank you for the professional comments. We agree that eutectogels have been explored for sensing applications owing to their unique properties, particularly in strain sensing. In the original manuscript, we used terms such as "piezoionic materials" and "piezoionic eutectogel biosensor" to emphasize the relatively limited exploration of eutectogels in piezoionic physiological sensing. However, we acknowledge that this phrasing may still lead to ambiguity. Accordingly, we have revised the relevant statements in the Introduction to improve clarity and avoid potential misunderstanding.

Our revision to the manuscript:

"However, despite these appealing attributes, prior investigations of eutectogels have primarily focused on resistive strain sensing, while their potential as biocompatible and self-powered piezoionic sensors remains largely unexplored." (**Section**: Introduction, **Paragraph**: 4)

We sincerely thank you once again for the insightful comments and the time devoted to evaluating our manuscript. These suggestions have been invaluable in improving the scientific rigor and clarity of the work. By integrating multi-participant vibration data and refining our claims regarding material robustness and novelty, we have established a more logic framework that better substantiates its board impact. y incorporating multi-participant vibration data and refining our discussion of material robustness and novelty, we have established a more logical framework that better supports the significance of our findings. We believe that these revisions have substantially strengthened the manuscript and have addressed the concerns raised.

Point-by-Point response to the reviewers' comments

We sincerely thank the reviewers for their careful and thorough review, which are indeed very helpful to make the paper more solid and smooth. We have revised our manuscript very carefully in light of their suggestions and comments.

The following responses have been prepared to address all of the reviewers' comments in a point-by-point fashion. (Comments in black, response in blue, and revisions highlighted in yellow):

Response to Reviewer #1

General comment: The authors submitted a revised version of their manuscript. This version is substantially improved and the authors have made a genuine effort to respond to reviewer comments. The added long-term stability data, the clarification of tensile versus compressive behavior, and the more detailed mechanistic interpretation strengthen the paper considerably. That said, the manuscript still feels somewhat overpacked, and some claims remain more ambitious than the evidence fully supports.

Response: We thank you for the encouraging evaluation and the recognition of our progress in optimizing stability performance and mechanistic interpretations.

In response to the remaining concerns, the manuscript has been further refined to improve clarity, concision, and balance. The **Abstract** and **Results and Discussion** sections have been streamlined to reduce narrative density and remove repetitive descriptions, thereby keeping the main text focused on the primary findings. Additionally, we have adjusted the phrasing throughout the manuscript to ensure that all claims remain consistent with the scope of our experimental and simulation evidence. Detailed implementations are specified in our responses to the individual comments below.

1. The manuscript would benefit from further tightening, especially in the Results and Discussion, where the narrative remains dense and occasionally repetitive. The scientific content is strong, but the presentation still reads as a heavily annotated revision rather than a fully streamlined final article.

Response: Thank you very much for your valuable suggestion. The manuscript has been further revised to improve concision, readability, and logical coherence. Specifically, the Abstract has been reorganized to present the central message more

directly, and the Results and Discussion section has been substantially streamlined, with the word count reduced from 3654 to 2720 words. Redundant experimental descriptions and overlapping explanations have been removed or condensed. In addition, several passages have been simplified and consolidated to improve logical flow, allowing the discussion to focus more closely on the key results.

The corresponding revisions have been highlighted in the revised manuscript.

2. Several claims should be moderated slightly so they match the data more precisely, particularly claims about novelty and the extent to which long-term stability and device integration have been "solved." The data supports a strong proof-of-concept, but not a universal resolution of the field's limitations.

Response: Thank you for the professional suggestion. We have moderated the relevant claims throughout the manuscript to ensure that they are aligned with the experimental evidence. In particular, absolute terms such as "highest" and "rare" have been removed or replaced with more objective descriptions. Claims related to novelty, long-term stability, and device integration have also been revised to avoid implying that the present work provides a universal solution to broader field-wide challenges.

The corresponding revisions have been highlighted in the revised manuscript.

3. A few figure-related issues should be checked one more time before acceptance, including consistency of labels, nomenclature, and cross-references between the main text and Supplementary Information. These are minor on their own, but in aggregate they affect readability and confidence.

Response: Thank you for this careful suggestion. All figures, captions, sample labels, nomenclature, and cross-references between the main text and Supplementary Information have been carefully checked to ensure their consistency and accuracy.

The corresponding revisions have been highlighted in the revised manuscript and Supplementary Information, particularly in the figure captions and cross-references.

4. Improve sentence-level language for clarity and concision in several sections, especially where the revision currently repeats the rebuttal logic inside the manuscript.

Response: Thank you for the careful and insightful suggestion. We have further refined the language throughout the manuscript to improve clarity and concision. Several dense paragraphs have been simplified and consolidated to ensure a more direct

narrative flow. Repetitive descriptions and defensive explanations have also been removed or rewritten as direct scientific statements.

The corresponding revisions have been highlighted in the revised manuscript.

5. Ensure all descriptors for DES20, DES50, DES70, etc. are defined clearly at first use and used consistently throughout the manuscript and captions.

Response: Thank you for raising this point. The descriptors for DES20, DES50, DES70, and related eutectogel samples have been clearly defined at their first appearance and checked throughout the manuscript and figure captions to ensure consistent usage.

Our revision to the manuscript:

"Specifically, "DES20" and "DES70" denote the eutectogels containing with 20 wt% and 70 wt% DES, respectively." ((**Section**: Results and Discussion, **Paragraph**: 1))

"Considering the simultaneous requirements for mechanical robustness and efficient piezoionic transduction, DES50, defined as the eutectogel containing 50 wt% DES, was selected for piezoionic output evaluation" (**Section**: Results and Discussion, **Paragraph**: 5)

6. Consider making the abstract slightly shorter and more direct, emphasizing the central message: a biocompatible DES-PVA eutectogel with strong piezoionic response and practical wearable sensing performance.

Response: Thank you for your constructive suggestion. The Abstract has been restructured to emphasize the core message.

Our revision to the manuscript:

"Piezoionics holds promise for self-powered bioelectronics, yet existing hydrogel and elastomer systems are often limited by poor biocompatibility, environmental instability, and low output. Here, we report a biocompatible deep eutectic solvent-polyvinyl alcohol (DES-PVA) eutectogel featuring a robust piezoionic response and practical wearable sensing capabilities. By incorporating a choline chloride-glycerol DES into a PVA network, we engineer tailored hydrogen bonds that selectively constrain choline cations while enabling rapid chloride anion transport, thereby amplifying the piezoionic response to achieve a sensitivity of 60 mV kPa^{-1}. In vitro assays verify the excellent biocompatibility of the eutectogel. Furthermore, practical

application demonstrations underscore its capability for precise and continuous monitoring of diverse physiological signals, including pulse waveform and electrocardiograms (ECG), with fidelity comparable to commercial sensors. Overall, this work establishes a self-powered, bio-compliant epidermal platform for advanced iontronics and bioelectronics." (**Section**: Abstract)

Response to Reviewer #2

General comment: Although the authors have revised the manuscript to some extent, the following issues remain unresolved. Hence, i do not think this paper can reach Nat. Commun.'s scientific level, and should be transferred to other journals.

Response: We thank you for the careful assessment of our revised manuscript. We have considered each of the remaining concerns and have further revised the manuscript accordingly. In particular, we have corrected the schematic representation of the piezoionic potential, added literature support for the vibrational assignments in Fig. 3j, and clarified the vocalization-related sensing. Furthermore, we have provided additional experimental validation for the selection of DES70 and revised the performance comparison in Fig. 2n. Detailed point-by-point responses and the corresponding revisions are provided below.

1. In Fig. 1, the schematic representation of the "Piezoionic potential" is incorrect. The authors should treat this with greater rigor.

Response: Thank you for raising this important point. Based on established literature (***Adv. Funct. Mater.*** 2026, 36, e75275; ***Adv. Mater.*** 2024, 36, 2313127; ***Science*** 2022, 376, 502), we have revised the schematic representation of the piezoionic potential in Scheme 1. The corresponding piezoionic potential profile has been updated to show a rapid voltage increase upon pressure, followed by a relaxation profile after pressure release.

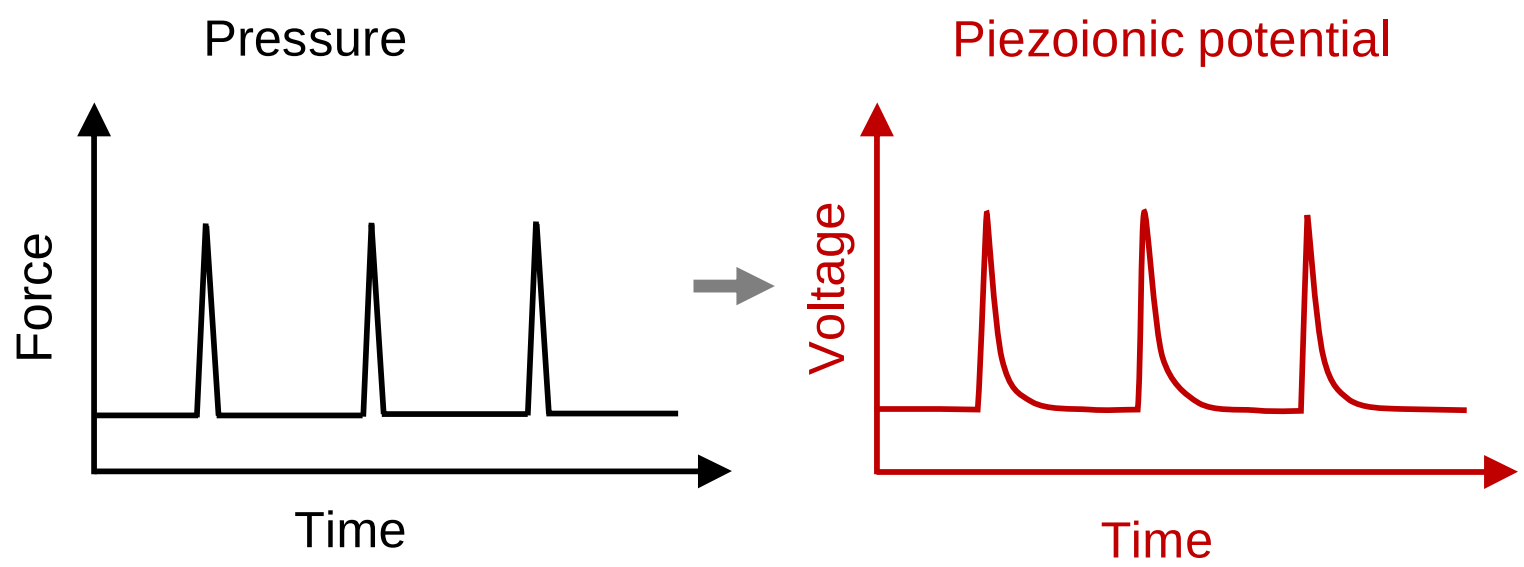

Fig. R1 Schematic representation of the applied pressure and the corresponding piezoionic potential.

Our revision to the manuscript:

The Fig. R1 has been incorporated into Scheme 1.

2. In Fig. 3j, please explicitly list relevant references for the chemical bonds indicated at different wavelength positions, particularly for "PVA-Ch^+" and "glycerol-$Cl^–$".

Response: We have summarized relevant references for the chemical bonds assigned to the characteristic bands at different wavenumbers, as shown in Table R1. For each characteristic bonds, at least three references have been listed to support the reliability of these assignments.

For the O-H stretching vibration associated with glycerol-$Cl^–$ interactions, previous studies on deep eutectic solvent (DES) systems composed of glycerol and choline chloride (ChCl) have confirmed that $Cl^–$ typically acts as a bridge between Ch^+ and glycerol through electrostatic attraction with Ch^+ and hydrogen bonding with glycerol, thereby forming a stable system (***J. Phys. Chem. Lett.*** 2024, 15, 11, 3002; ***Phys. Chem. Chem. Phys.*** 2018, 20, 22455). Gulsah Karabulut et al. explicitly reported that the O-H stretching bands at 3284 $cm^{–1}$ originate from strong hydrogen bonding within ChCl: glycerol DES systems (***Int. J. Biol. Macromol.*** 2025, 328, 147556.). Wang et al. further assigned the infrared band at 3267 $cm^{–1}$ to the O-H stretching vibration of O-H···$Cl^–$ hydrogen bonding of ChCl: glycerol DES systems (***ACS Sustainable Chem. Eng.*** 2019, 7, 8, 7760). Considering these reported band positions differences, together with differences in material sources and the pressure-induced band shifts, the assignment of the 3278 $cm^{–1}$ band in our work is well supported to the O-H stretching vibration associated with glycerol-$Cl^–$ interactions.

For the PVA-Ch^+ interaction, previous studies have confirmed the formation of hydrogen bonding between PVA and Ch^+ (***Angew. Chem. Int. Ed.***, 2025, 64, e202418223; ***Eur. Polym. J.*** 2020, 133, 15). Compared with glycerol-$Cl^–$, the hydrogen bonds between PVA and Ch^+ (PVA-Ch^+) is expected to appear at a relatively higher O-H stretching wavenumber (***Proc. Natl. Acad. Sci.*** 2028, 105, 5295; ***Phys. Chem. Chem. Phys.*** 2024, 26, 9432; ***Langmuir*** 2019, 35, 23, 7388), which is consistent with our assignment. Together with the reported effective hydrogen bonding between PVA and Ch^+ (***ACS Appl. Mater. Interfaces*** 2022, 14, 43, 49212), the PVA-DES hydrogen bonding in our system can be reasonably attributed mainly to PVA-Ch^+ interactions. Huo et al. confirmed that the infrared band at 3286 $cm^{–1}$ originates from the O-H stretching vibration of PVA-DES interactions (***Adv. Funct. Mater.*** 2025, 35, 2422464). Additional references listed in Table R1 report hydroxyl vibration bands associated with PVA-Ch^+ or PVA-DES interactions at 3281, 3290, and 3291 $cm^{–1}$, which are consistent with the 3291 $cm^{–1}$ band assigned to PVA-Ch^+-associated hydroxyl vibration in our work.

We have also provided references for the assignments of the bands at 3544 cm^{-1} and 2998 cm^{-1} observed in Figure 3j. The band at 3544 cm^{-1} can be assigned to the O-H stretching vibration of relatively free hydroxyl groups with weak hydrogen bonding, as reported in previous studies (***Adv. Energy Mater.*** 2024, 14, 2400314; ***ACS Sustainable Chem. Eng.*** 2019, 7, 8, 7760; ***J. Agric. Food Chem.*** 2013, 61, 3212). This assignment is consistent with the general principle that hydroxyl groups with weaker hydrogen bonding exhibit higher stretching frequencies. In addition, the infrared bands in the 2900–3000 cm^{-1} region have been clearly assigned to C-H stretching vibrations (***J. Spectrosc.*** 2025, DOI: 10.1155/jspe/1359636). Previous reports also support that bands near 2990 cm^{-1} originate from C-H stretching vibrations (***Polymer***, 2024, 293, 126673; ***ACS Omega***, 2020, 5, 19350), consistent with our assignment of the 2998 cm^{-1} band.

Based on the references summarized above, the characteristic bands in Fig. 3j are well supported by reported infrared band position. Accordingly, we have added the relevant references to substantiate the attribution of these bands to the corresponding functional groups.

Table R1. References of chemical bonds in our work and previous reports

**Chemical bonds**	**Bond type**	**Materials**	**Wavelength (cm^{-1})**	**Vibration mode**	**Ref.**
-OH	Hydrogen bond (Gly-Cl^-)	Gly:ChCl	3284	Stretching	1
		Gly:ChCl	3286	Stretching	2
		OH-Cl^- (Gly:ChCl)	3267	Stretching	3
		OH-Cl^-	3278	Stretching	Our work
-OH	Hydrogen bond (PVA-Ch^+)	PVA-DES (ChCl-Gly)	3286	Stretching	4
		PVA-DES (ChCl-Gly)	~3290	Stretching	5
		PVA-ChCl	3291	Stretching	6
		PVA-ChCl	3291	Stretching	Our work
-OH	Hydrogen bond (PVA-PVA)	PVA-PVA	3210	Stretching	4
		PVA-PVA	3210	Stretching	7
		PVA-PVA	3210	Stretching	9
-OH	Relatively free hydroxyl group	ChCl-AA-Gly	3506	Stretching	3
		Alcohols	3554	Stretching	9

		Gly	3550	Stretching	10
		PVA-DES	3544	Stretching	Our work
C–H	C–H group	PVA	2900–3000	Stretching	11
		PVA	2976	Stretching	12
		PVA	2947, 2980	Stretching	13
		PVA	2995	Stretching	14
		DES (ChCl-Gly)	2965	Stretching	15
		DES (ChCl-Gly)	2978	Stretching	16
		PVA/DES	2998	Stretching	Our work

[1] Karabulut, G. et al. Green extraction using natural deep eutectic solvents for the recovery of proteins from sunflower meal. *Int. J. Biol. Macromol.* **328**, 147556 (2025).

[2] Chen, H. et al. Reaction characteristics of metal-salt coordinated deep eutectic solvents during lignocellulosic pretreatment. *J. Environ. Chem. Eng.* **11**, 109531 (2023).

[3] Wang, H. et al. Insights into the Hydrogen Bond Interactions in Deep Eutectic Solvents Composed of Choline Chloride and Polyols. *ACS Sustainable Chem. Eng.* **7**, 7760–7767 (2019).

[4] Huo, X. et al. Strong and Tough Eutectogels with Broad-Range Tunable Mechanical Properties via the Hydrogen Bond Network-Specific Effect. *Adv. Funct. Mater.* **35**, 2422464 (2025).

[5] Chen, S. et al. Facile Solvent Regulation for Highly Strong and Tough Physical Eutectogels with Remarkable Strain Sensitivity. *ACS Appl. Mater. Interfaces* **15**, 44752–44762 (2023).

[6] Zheng, L. et al. PVA/ChCl Deep Eutectic Polymer Blends for Transparent Strain Sensors with Antifreeze, Flexible, and Recyclable Properties. *ACS Appl. Mater. Interfaces* **14**, 49212–49223 (2022).

[7] Guo, B. et al. Anisotropic conductive eutectogels for strain sensing and triboelectric nanogeneration in extreme environments. *J. Colloid Interface Sci.* **679**, 906–917 (2025).

[8] Tang, N. et al. Evolutionary Reinforcement of Polymer Networks: A Stepwise-Enhanced Strategy for Ultrarobust Eutectogels. *Adv. Mater.* **36**, 2309576 (2024).

[9] Tena, N. et al. Time Course Analysis of Fractionated Thermoxidized Virgin Olive Oil by FTIR Spectroscopy. *J. Agric. Food Chem.* **61**, 3212–3218 (2013).

[10] Zhang, X. et al. Regulated Hydrated Eutectic Electrolyte Enhancing Interfacial Chemical Stability for Highly Reversible Aqueous Aluminum-Ion Battery with a Wide Temperature Range of -20 to 60 °C. *Adv. Energy Mater.* **14**, 2400314 (2024).

[11] Wu, N. et al. Study of the Effect of Temperature and Microwave Action on the Structure of Polyvinyl Alcohol (PVA). *J. Spectrosc.* DOI: 10.1155/jspe/1359636 (2025).

[12] Devi, L. S. et al. Preparation of cross-linked PANI/PVA conductive hydrogels for electrochemical energy storage and sensing applications. *Polymer*, **293**, 126673 (2024).

[13] Alakanandana, A. et al. Structural and electrical conductivity studies of pure PVA and PVA doped with succinic acid polymer electrolyte system. *Mater. Today Proc.* **310**, 3680–3688 (2016).

[14] Das, M. et al. Development of room temperature ethanol sensor from polypyrrole (PPy) embedded in polyvinyl alcohol (PVA) matrix. *Polym. Bull.* **75**, 3109–3125 (2018).

[15] Banjare, R. K. et al. Micellization behavior of conventional cationic surfactants within glycerol-based deep eutectic solvent. *ACS Omega*, **5**, 19350–19362 (2020).

[16] Banjare, M. K. et al. Self-assembly of a short-chain ionic liquid within deep eutectic solvents. *RSC Advances*, **8**, 7969–7979 (2018).

Our revision to the manuscript:

The corresponding references have been added to the revised manuscript to support the assignment of the characteristic bands in Fig. 3j.

"Based on Noda's judging rule[42–44], the pressure-responsive sequence follows 3278 cm^{-1} → 3291 cm^{-1} → 3544 cm^{-1} → 2998 cm^{-1}, where the arrow denotes an earlier response, corresponding respectively to ν(-OH, glycerol-Cl^-), ν(-OH, PVA-Ch^+), ν(-OH, free), ν(-CH_2, stretching), respectively[45–49]." (**Section**: Results and Discussion, **Paragraph**: 10)

"45. Karabulut, G. et al. Green extraction using natural deep eutectic solvents for the recovery of proteins from sunflower meal. *Int. J. Biol. Macromol.* **328**, 147556 (2025).

46. Wang, H. et al. Insights into the hydrogen bond interactions in deep eutectic solvents composed of choline chloride and polyols. *ACS Sustainable Chem. Eng.* **7**, 7760–7767 (2019).

47. Huo, X. et al. Strong and tough eutectogels with broad-range tunable mechanical properties via the hydrogen bond network-specific effect. *Adv. Funct. Mater.* **35**, 2422464 (2025).

48. Zheng, L. et al. PVA/ChCl deep eutectic polymer blends for transparent strain sensors with antifreeze, flexible, and recyclable properties. *ACS Appl. Mater. Interfaces* **14**, 49212–49223 (2022).

49. Wu, N. et al. Study of the effect of temperature and microwave action on the

structure of Polyvinyl Alcohol (PVA). *J. Spectrosc*. DOI: 10.1155/jspe/1359636 (2025)." (**Section**: References)

3. The authors state, "During speech or swallowing, vocal cord vibration induces localized deformation of the overlying skin, generating compressive stresses on the order of 0.5–1.5 kPa." It seems unlikely that such high pressure can be achieved without back pressure. How was this value determined? Furthermore, the PU membrane mentioned by the authors appears to vibrate synchronously with the sensor and does not seem to provide a supporting function.

Response: Thank you for raising this question. We would like to clarify that the pressure range of 0.5–1.5 kPa was taken from previous reports (***J. Acoust. Soc. Am.*** 2016, 139, 1493; ***J. Speech Lang. Hear. Res.*** 2005, 48, 567; ***J. Speech Lang. Hear. Res.*** 2001, 44, 29), rather than being measured in our work. We further emphasize that the vocal vibration signals in our study were experimentally collected from three male volunteers and two female volunteers. The comparable piezoionic voltage profiles obtained for the same phonation features support the reliability and reproducibility of the detected signals. The back pressure mentioned here is not a necessary requirement in our work, but is related to the specific testing configuration.

As illustrated in Fig. R2a, back pressure is primarily relevant to sandwich-type configurations, rather than to the parallel-electrode configuration used in our work. We understand that the back pressure refers to an opposing force applied on the side opposite to the loading surface, which helps establish an effective deformation or stress gradient along the thickness direction. In a sandwich structure, the measured potential difference is generated along the thickness direction of the piezoionic material, and the corresponding stress gradient must therefore also be established through the thickness. Without back pressure, it is difficult to form a well-defined stress gradient along the thickness direction because the effective distance between the two electrodes is extremely short.

By contrast, in a parallel-electrode configuration, the stress gradient is established along the length direction of the piezoionic material. Such a configuration has been reported for physiological signal monitoring without obvious back pressure (***Adv. Funct. Mater.*** 2026, 36, e75275). In this geometry, the effective distance between the two electrodes can be adjusted, enabling a pronounced deformation gradient to be established along the length direction when pressure is locally applied near one end. Thus, back pressure is not required because the region far

from the loading site can serve as an effective mechanical counterpart for establishing the stress gradient. As emphasized in our previous response, our eutectogel sensor adopts a planar two-electrode configuration, in which the two electrodes are separated by a sufficient distance to establish this in-plane pressure gradient (Fig. R2b).

We agree that the PU film vibrates synchronously with the sensor. This behavior is expected for a flexible skin-mounted device and highlights its advantage in wearing comfort. The PU film mainly serves as a conformal fixation and encapsulation layer to maintain stable contact between the eutectogel and the skin. In contrast, using a hard or rigid material to provide back pressure would compromise the feasibility of wearable applications.

(Redacted)

4. The authors did not conduct any experiments for DES content greater than 70%. How was it determined that DES70 is the optimal concentration? Moreover, the authors did not use DES70 in the majority of experiments throughout the manuscript, including "biocompatibility, voltage and current under cyclic compression." Why was DES70 switched to only for the application demonstration?

Response: Thank you for raising these questions. **We would like to emphasize that DES70 was not introduced only for the ECG application demonstration, but had already been included in a series of fundamental characterizations and performance evaluations.** Specifically, the structural and physicochemical properties of DES70 were characterized by SEM, FTIR, XRD, Raman spectroscopy, and Raman mapping, as shown in Fig. 1c, Fig. 1d, Fig. 1e, Fig. 1f, and Supplementary Fig. 4. Its mechanical properties were evaluated in Fig. 2a and Fig. 2b, while its piezoionic voltage response, piezoionic current response, and ionic conductivity were examined in Fig. 2e, Fig. 2h, Supplementary Fig. 9, and Supplementary Fig. 12. Therefore, the use of DES70 in the application demonstration is supported by comprehensive structural, mechanical, electrical, and piezoionic characterizations.

For biocompatibility, DES50 already represents a relatively high DES fraction and showed cell viability comparable to that of DES10 in the cytotoxicity assay, supporting the good biocompatibility of the eutectogel system. Nevertheless, to further substantiate the biocompatibility of DES70, we performed additional in vitro cell viability assays. As shown in Fig. R3, after 24 h of co-culture, cells cultured with the DES70 exhibited

viability and adhesion comparable to those cultured with DES50, confirming the good biocompatibility of DES70.

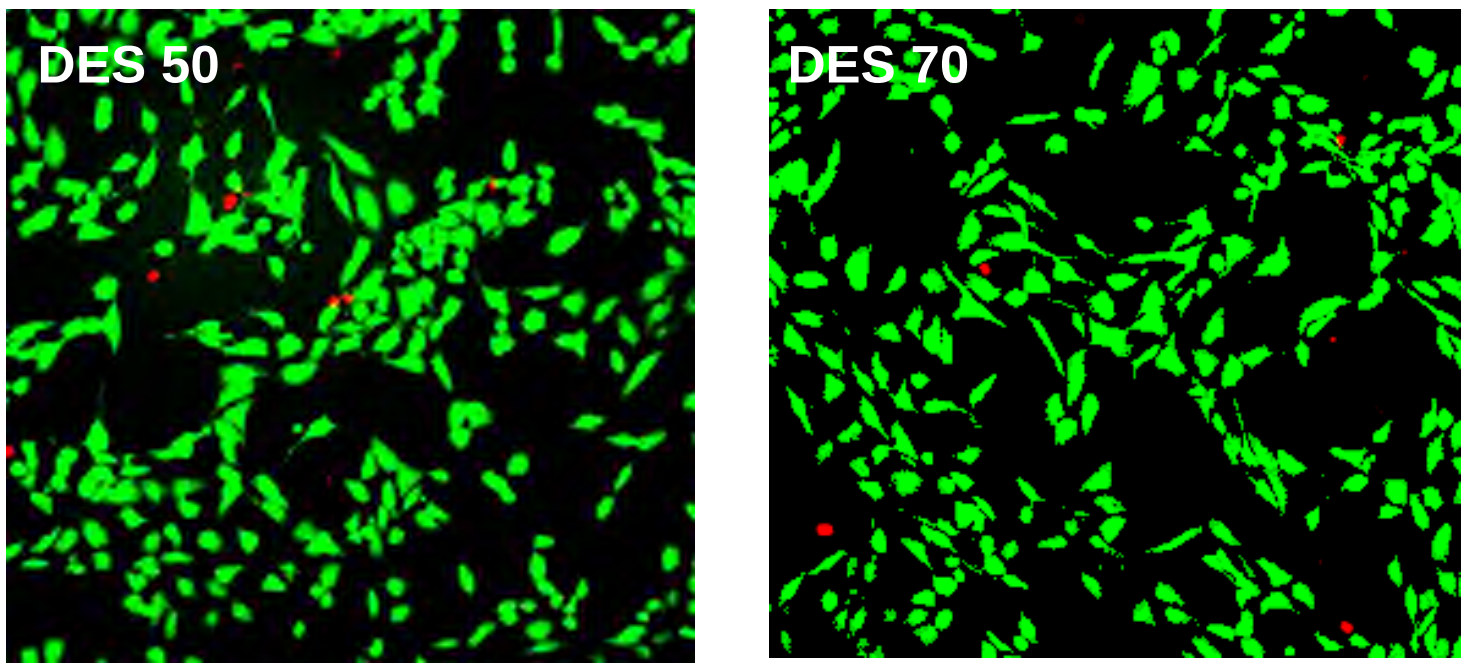

Fig. R3. Cell viability of DES50 and DES70

For voltage and current outputs under cyclic compression, DES50 was selected as the representative sample because it was used for piezoionic performance evaluation and piezoionic sensing demonstrations, where the repeated mechanical stimulation was involved in our previous manuscript. **In contrast, DES70 was mainly used for bioelectrical signal monitoring, where the device is conformally attached to the skin and does not undergo repeated mechanical compression during operation. Therefore, cyclic compression testing is not the most relevant evaluation for assessing DES70 in ECG monitoring**. In our previous response, we emphasized that DES70 was selected for ECG recording because of its high ionic conductivity and appropriate Young's modulus. To further demonstrate the long-term stability of DES70 for ECG recording, we performed additional ECG measurements using a DES70 sample that had been stored for 6 months. As shown in Fig. R4, the ECG signals remained stable and were highly consistent with those obtained in the previous measurements.

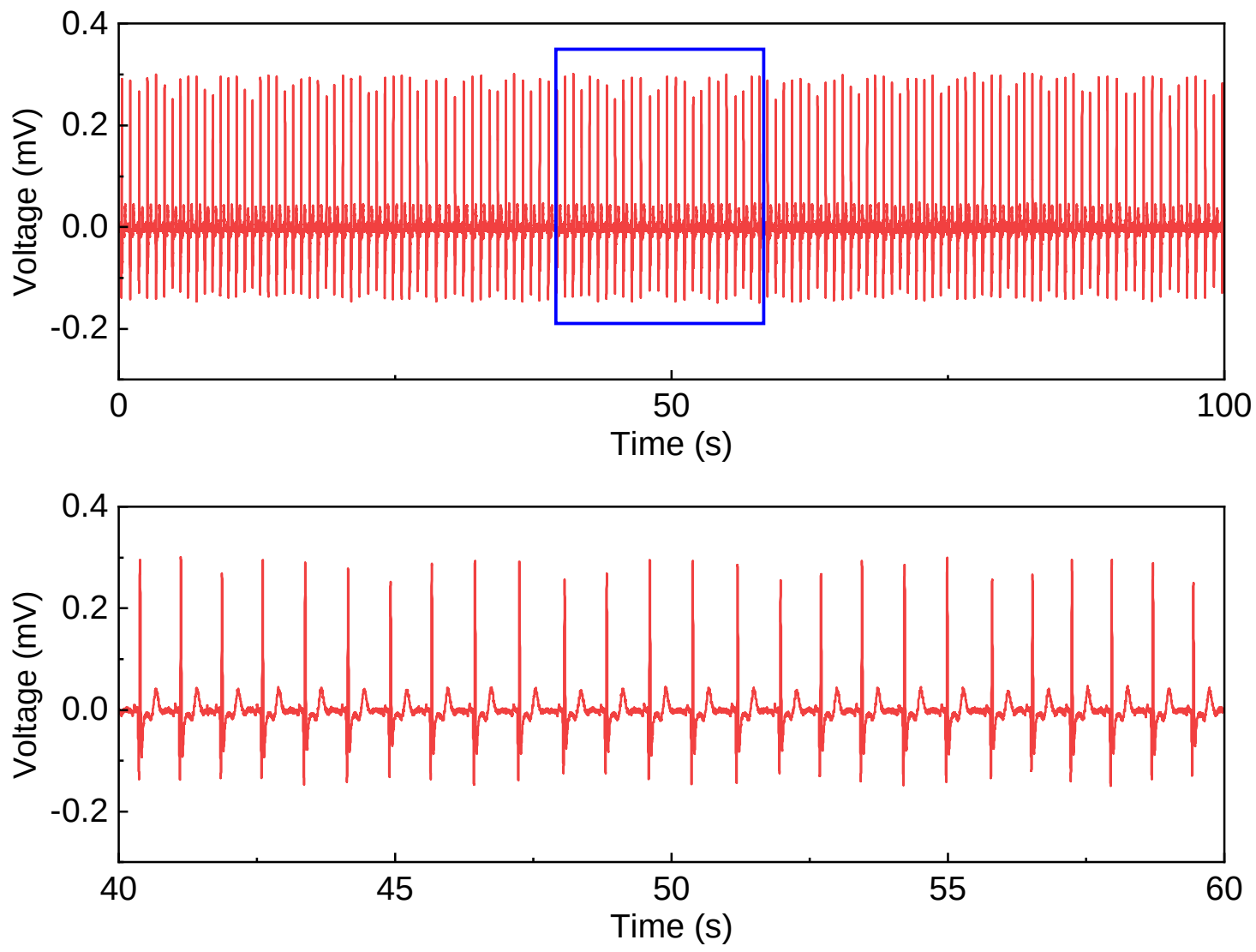

Fig. R4 ECG signals recorded using DES70 after 6 months of storage

Regarding the concern that eutectogels with DES contents higher than 70 wt% were not experimentally examined, we clarify that Supplementary Fig. 3 already showed the aqueous PVA/DES precursor solution for DES80, which exhibits pronounced microphase separation. **This observation was also discussed in the initial manuscript: "the miscibility between PVA and glycerol remains limited, restricting the maximum DES content that can be homogeneously incorporated (Supplementary Fig. 3)"**. This phase separation is accompanied by rapid gelation of the solution (Fig. R5a), which is unfavorable for the subsequent film-forming process. To obtain DES80 and DES90, the heated PVA/DES precursor solutions had to be rapidly transferred into molds before gelation occurred. Although eutectogels could still be formed, the resulting films were difficult to demold and exhibited poor structural integrity (Fig. R5b), indicating compromised mechanical robustness. **We have emphasized this point in our previous response: "It should also be noted that although increasing the DES content further enhances ionic conductivity, it simultaneously leads to a pronounced deterioration in mechanical properties"**. To further substantiate this point experimentally, we selected a relatively intact DES80 sample for mechanical testing. As shown in Fig. R6, DES80 exhibits both low fracture strength and low fracture strain, demonstrating that excessive DES loading severely compromises mechanical reliability and therefore makes these high-DES-content eutectogels unsuitable for long-term application.

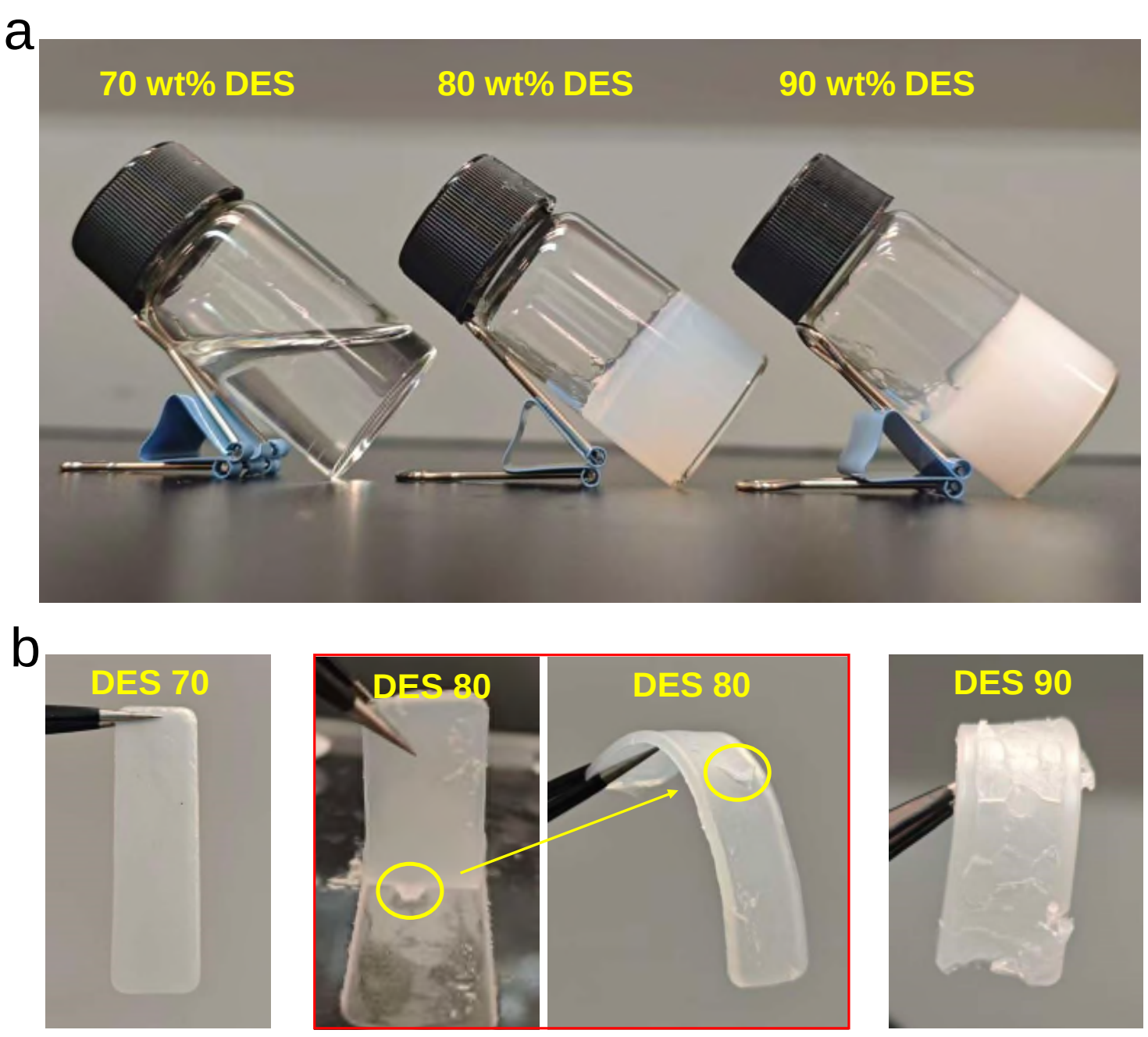

Fig. R5 (a) Aqueous PVA/DES precursor solution for DES70, DES80, and DES90. (b) Photographs of DES70, DES80, and DES90.

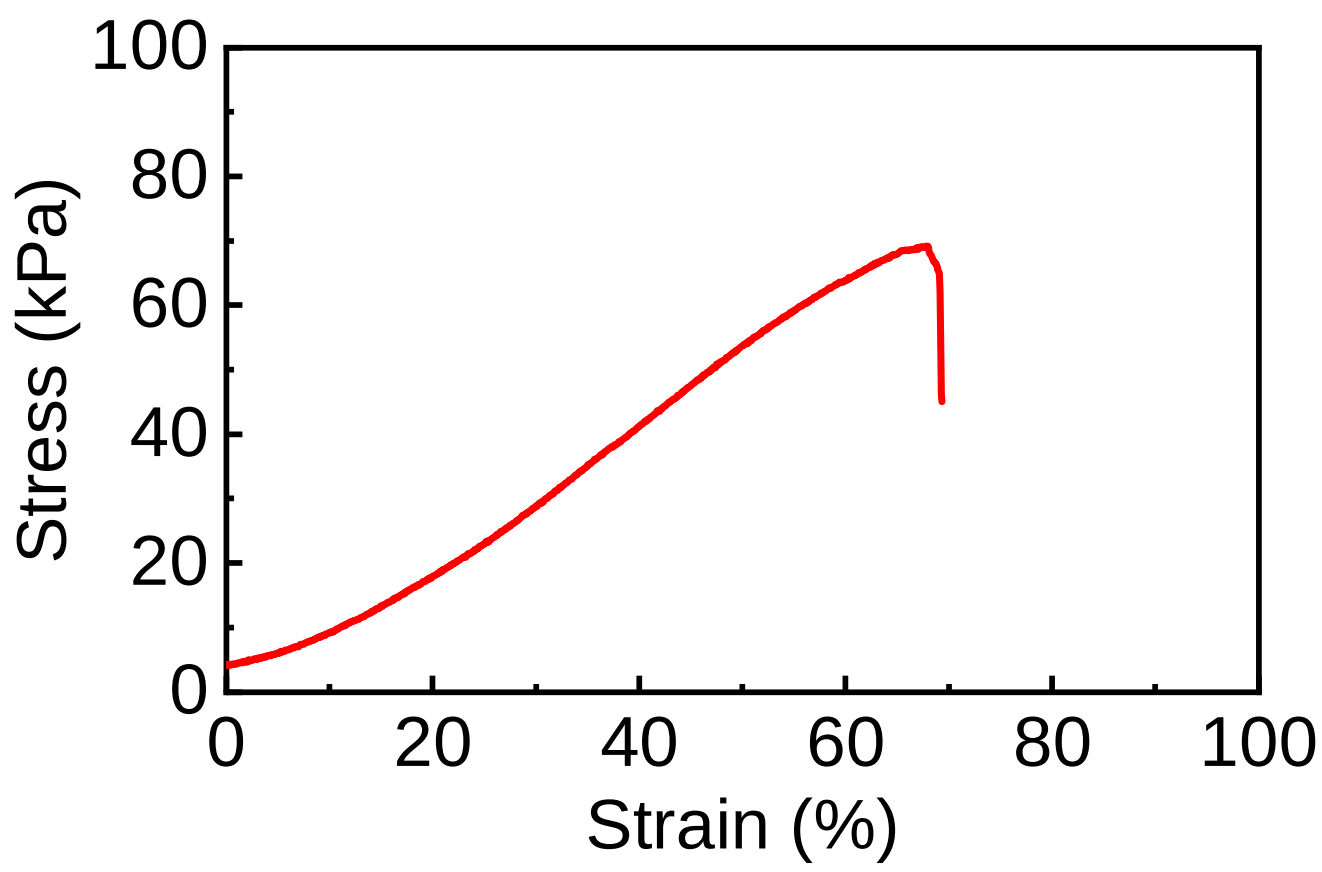

Fig. R6 Stress-strain curves of DES80

Taken together, the characterization and testing results presented in the manuscript, along with the newly added DES70 cell viability data, ECG stability results, and additional evidence from DES80 and DES90, provide a clear rationale for selecting DES70 as the optimal formulation for ECG signal monitoring.

Our revision to the manuscript:

The cell viability image of DES50 in Fig. 1h has been replaced with Fig. R3, and Fig. R4 has been added as Supplementary Fig. 26. Fig. R5a has also been integrated

into Supplementary Fig. 3. Corresponding changes have been highlighted in the revised manuscript and supplementary information.

"At a high DES content of 80 wt%, the system showed pronounced microphase separation. This phase separation is accompanied by rapid gelation of the solution, which is unfavorable for the subsequent film-forming process." (**Section**: Supplementary Information, **Paragraph**: Supplementary Fig. 3)

"In contrast, DES-PVA eutectogels supported dense and uniform cell adhesion, maintaining good cell viabilities at both 10 wt% and 70 wt% DES loadings." (**Section**: Results and Discussion, **Paragraph**: 3)

"The signal quality and signal-to-noise ratio are comparable to those obtained using commercial hydrogel electrodes (Supplementary Fig. 25), and long-term stability has also been confirmed (Supplementary Fig. 26)." (**Section**: Results and Discussion, **Paragraph**: 13)

5. In Fig. 2n, when performing the comprehensive performance comparison, the authors combined the best advantages of samples with different DES concentrations. This does not represent a single sample. For instance, how were the values for "maximum voltage 80, ionic conductivity 3, Young's modulus 24" achieved simultaneously?

Response: Thank you for your careful comment. In our work, DES50 and DES70 serve as the two main samples for different functional demonstrations, namely piezoionic sensing and ECG signal monitoring. Therefore, Fig. 2n has been revised to present the performance metrics of these two representative samples. Specifically, the maximum voltage, maximum current, piezoionic coefficient, and power density are derived from DES50, whereas the ionic conductivity and Young's modulus are derived from DES70. This revised comparison more accurately reflects the application performance of the eutectogels.

In addition, the comparative references have been updated by including recently reported piezoionic materials[1–8]. As shown in Fig. R7, the DES-PVA eutectogels still exhibit a balanced and competitive performance profile. The corresponding references have also been added to the revised manuscript.

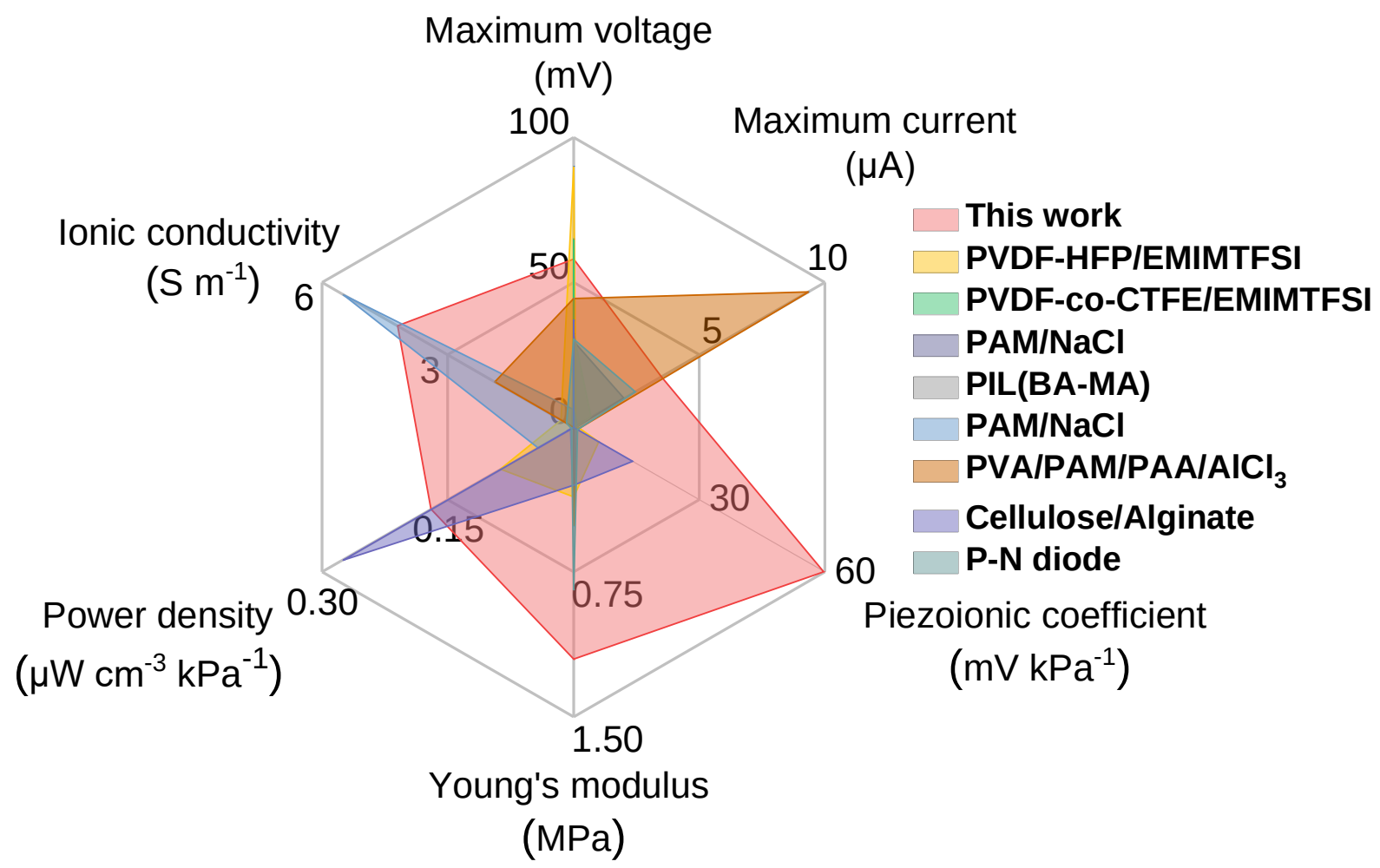

Fig. R7 Performance comparison of DES-PVA eutectogels with recently reported piezoionic materials.

[1] Guan, X. et al. Spider Webs-Inspired Aluminum Coordination Hydrogel Piezoionic Sensors for Tactile Nerve Systems. ***Adv. Funct. Mater.*** 2025, 35, 2414016.

[2] Li, J-D. et al. Phase-Separation Enhanced Piezoionic Effect in Cellulose/Alginate Hydrogels for High-Sensitivity Self-Powered Sensing. ***Adv. Funct. Mater.*** 2026, DOI: 10.1002/adfm.75275.

[3] Li, B. et al. The Piezoionic Diode: Field-Driven Amplification of Mechano-Ionic Conversion. ***Mater. Horiz.*** 2026, DOI: 10.1039/D6MH00251J.

[4] Zhu, W. et al. Piezoionic Elastomers by Phase and Interface Engineering for High-Performance Energy-Harvesting Ionotronics. ***Adv. Mater.*** 2024, 36, 2313127.

[5] LV, D. et al. Microphase-Separated Elastic and Ultrastretchable Ionogel for Reliable Ionic Skin with Multimodal Sensation. ***Adv. Mater.*** 2024, 36, 2309821.

[6] Lu, X. et al. Piezoionic High Performance Hydrogel Generator and Active Protein Absorber via Microscopic Porosity and Phase Blending. ***Adv. Mater.*** 2024, 36, 2307875.

[7] Wang, D. et al. Soft self-powered solid-state poly(ionic liquid) piezoionic elastomer sensor for extreme temperature operation. ***Chem. Eng. J.*** 2026, 530, 173003.

[8] Dobashi, Y. et al. Piezoionic mechanoreceptors: Force-induced current generation in hydrogels. ***Science*** 2022, 376, 502.

Our revision to the manuscript:

Fig. R7 has been incorporated into Fig. 2n, and the corresponding references have been updated in the revised manuscript.

"As summarized in Fig. 2n, the proposed eutectogel exhibits a balanced and high-performance profile, achieving a piezoionic voltage of approximately 60 mV, a current of 3.5 μA, a piezoionic coefficient of 59.8 mV kPa^{-1}, a Young's modulus of 1.2 MPa, an ionic conductivity of 4.2 S m^{-1}, and a normalized power density of 0.17 μW cm^{-3} kPa^{-1}." (**Section**: Results and Discussion, **Paragraph**: 6)

"34. Guan, X. et al. Spider Webs-inspired aluminum coordination hydrogel piezoionic sensors for tactile nerve systems. *Adv. Funct. Mater.* **35**, 2414016 (2025).

35. Lv, D. et al. Microphase-separated elastic and ultrastretchable ionogel for reliable ionic skin with multimodal sensation. *Adv. Mater.* **36**, 2309821 (2024).

36. Lu, X. et al. Piezoionic high performance hydrogel generator and active protein absorber via microscopic porosity and phase blending. *Adv. Mater.* **36**, 2307875 (2024).

37. Li, J-D. et al. Phase-separation enhanced piezoionic effect in cellulose/alginate hydrogels for high-sensitivity self-powered sensing. *Adv. Funct. Mater.* 2026, DOI: 10.1002/adfm.75275.

38. Li, B. et al. The piezoionic diode: Field-driven amplification of mechano-ionic conversion. *Mater. Horiz.* 2026, DOI: 10.1039/D6MH00251J.

39. Wang, D. et al. Soft self-powered solid-state poly(ionic liquid) piezoionic elastomer sensor for extreme temperature operation. *Chem. Eng. J.* **530**, 173003 (2026)." (**Section**: References)

Response to Reviewer #3

General comment: the manuscript has been revised appropriately and now can be accepted.

Response: Thank you for reviewing our revised manuscript and for your positive comment. We sincerely appreciate your valuable feedback on the previous version, which has helped us improve the quality and clarity of our work. We are pleased that our responses and revisions have addressed your concerns.
